# ALIX mediates reversible gasdermin-D pore formation via the endosomal pathway to limit pyroptosis by active membrane repair

**DOI:** 10.1038/s41419-025-07998-y

**Published:** 2025-10-06

**Authors:** Sylvia Otchere, Prafulla Shrestha, Himesh N. Parmar, Jadyn F. Perry, Brittany L. Hofmeister, Michelle Steyn, Radhey S. Kaushik, Adam D. Hoppe, Natalie W. Thiex, Ryan L. Hanson, Jaime Lopez-Mosqueda, Gergely Imre

**Affiliations:** 1 Department of Biology and Microbiology, South Dakota State University, Brookings, SD USA; 2 Department of Chemistry, Biochemistry, and Physics, South Dakota State University, Brookings, SD USA; 3 Department of Molecular Biosciences, The University of Texas at Austin, Austin, TX USA

**Keywords:** Cell death, Cell signalling

## Abstract

Pyroptosis is a form of regulatory cell death characterized by membrane rupture and release of pro-inflammatory signals. In pyroptosis, Caspase-1 activation leads to the cleavage of gasdermin-D (GSDMD). Upon cleavage, GSDMD’s N-terminal (N-GSDMD) fragments insert into the plasma membrane, oligomerize, and form pores. The molecular details that define whether GSDMD pore formation results in cell death or survival are largely unknown. In this study, we show that a shorter duration of membrane N-GSDMD pores (*t* ≤ 2 h), along with associated membrane permeability does not harm cellular viability. We demonstrate that N-GSDMD is removed, and membrane integrity is restored if the pyroptotic stimulus is washed out within 1 hour. In contrast, longer duration of N-GSDMD pore formation leads to large-scale membrane damage and cell death. Using a selective dynamin inhibitor and confocal microscopy, to co-label N-terminal GSDMD (N-GSDMD) and the early endosomal marker EEA1, we demonstrate that N-GSDMD is cleared from the plasma membrane via the endosomal pathway. Through stable ALIX knockdown and overexpression approaches, we further show that ALIX, a key accessory protein of the ESCRT machinery, regulates N-GSDMD pore dynamics by promoting its removal and facilitating membrane repair via N-GSDMD internalization. In summary, we show that the duration of N-GSDMD membrane pores is a decisive factor and ALIX-dependent mechanism facilitates N-GSDMD removal and restores membrane integrity. The identification of these factors can open the development of new therapeutic strategies in chronic inflammatory conditions by bolstering the cell’s inherent self-healing potential.

## Introduction

Pyroptosis is a form of regulatory cell death characterized by membrane rupture and release of damage associated molecular patterns. Pyroptosis is induced following tissue damage, microbial pathogens, and metabolic perturbations that activate inflammasome protein complexes [1]. In chronic inflammatory conditions, like inflammatory bowel disease [2, 3], rheumatoid arthritis [4], and type 1 diabetes [5], pyroptosis maintains a persistent inflammatory state. How pyroptosis susceptibility and resistance are regulated in macrophages, monocytes, and other pyroptosis susceptible cells, like intestinal epithelial cells, in these inflammatory diseases is largely unknown.

Pyroptosis is primarily induced following activation of the inflammatory caspase, caspase-1 by inflammasomes [6]. Caspase-1 has a critical role in processing interleukin (IL)-1β and IL-18 into their bioactive forms [7]. Caspase-1 activation also leads to the cleavage of gasdermin-D (GSDMD), a pore-forming protein that mediates pyroptosis [8]. Upon cleavage, GSDMD’s N-terminal (N-GSDMD) fragments insert into the plasma membrane, oligomerize, and form pores of an estimated diameter of 25 nm that are essential to release IL-1β and -18 into the extracellular space. GSDMS pores also lead to memebrane rupture and pyroptotic cell death [9]. However, we and others show that pyroptosis induction does not essentially lead to cell death in every cell even in a seemingly homogenous cell population, and GSDMD pore formation alone is not always lethal [10, 11].

The formation of GSDMD pores leads to K+ efflux and Ca2+ influx into the cytoplasm [12]. It was recently shown that the influx of Ca2+ during GSDMD pore formation triggers a membrane remodeling complex termed endosomal sorting complexes required for transport (ESCRT) [13]. The primary function of ESCRT is to facilitate the sorting and trafficking of membrane proteins within the cell [14, 15]. ESCRTI plays a significant role in facilitating membrane scission events leading to budding of viruses, including HIV [16]. ESCRTII facilitates packaging endocytic vesicles into multi vesicular bodies (MVBs) in the endosomal pathway [17]. In both pathways, ESCRTIII accomplishes the membrane abscission event. ALG-2-interacting protein X (ALIX or PDCD6IP) [18] plays multiple roles in ESCRT-dependent functions, including bridging ESCRTI with ESCRTIII to initiate membrane curvature and direct exosome formation, connecting ESCRTII with ESCRTIII in MVB formation, and separation of daughter cells in cytokinesis [15, 19, 20].

In this study, we show that a limited duration (*t* < 2 h) of membrane GSDMD pores with accompanying membrane permeability is not detrimental. Conversely, longer duration (21 h) of pore formation leads to large-scale membrane damage and cell death. This supports the hypothesis that the decisive factor is the duration of the membrane N-GSDMD pores and the accompanying membrane permeability. By using stable ALIX-knockdown cells and ALIX overexpression, we provide evidence that ALIX participates in N-GSDMD pore membrane dynamics and that it is a critical regulator of N-GSDMD clearance and restoration of membrane integrity.

## Materials and methods

### Cell culture

HeLa cells (ATCC, Gaithersburg, MD, USA, CCL-2) and HCT-116 (ATCC, CCL-247) cells were maintained in Dulbecco’s Modified Eagle Medium (DMEM, Gibco/Thermo Fisher Scientific, Waltham, MA) supplemented with 10% fetal bovine serum (FBS, Biowest, Bladenton, FL, USA, S1480), 100 μg/ml streptomycin and 100 units/ml penicillin (Corning, Glenndale, AZ, USA, 30-002-cl). THP-1 cells (ATCC, TIB-202) were maintained in Roswell Park Memorial Institute (RPMI) -1640 medium (Gibco), supplemented with 10% FBS, 100 μg/ml streptomycin, and 100 units/ml penicillin. The cells were incubated in T-75 cell culture flask at 37 °C with 5% CO_2_. For experiments, cells were seeded in 6- or 12-well cell culture plates.

### Reagents

α-Hemolysin from *Staphylococcus aureus* (α-toxin) was purchased from Millipore Sigma (Darmstadt, Germany, H9395) and prepared in sterile distilled water. Nigericin from *Streptomyces hygroscopicus (S6653) and* dynasore (S8047) were obtained from Selleckchem (Houston, Texas, USA) and dissolved in dimethyl sulfoxide (DMSO).

### Immunoblotting

Cell lysates in Laemmli were run in 10% SDS-PAGE. Next, the gels were transferred to nitrocellulose membrane. Membranes were probed for indicated primary antibodies overnight and corresponding fluorescent secondary antibodies for 1 h (Licor, Lincoln, Nebraska, USA). The membranes were detected by using Odyssey Fc (Licor). Following antibodies were employed: Beta-actin (Abcam, Waltham, MA, USA, ab8226) GSDMD cleaved N-terminal (Abcam, ab255983), GSDMD (Novusbio, Centennial, CO, USA, NBP2-33422), TSG101 (Abcam, ab83), ALIX (Novusbio, 90201), GAPDH (Millipore Sigma, SAB4300645).

### Membrane permeability detection by flow cytometry

0.5 × 10⁶ cells were seeded in a 12-well plate and treated as indicated. Cells were then harvested and incubated with either 1 μg/ml propidium iodide (Sigma, P4864) or 1 μg/ml 7-Amino Actinomycin D (7-AAD) (Sigma, SML1633) RT for 15 min before subjected to flow cytometry detection with Accuri C6 Plus (BD, Franklin Lakes, NJ, USA), using the FL2 channel (488 nm blue laser/ 585/40 nm band-pass filter). Cell debris (population exhibiting low FSC/FL2 intensity) was excluded from the analysis in FSC/FL2 dot-plot. To measure large-scale membrane rupture, cells were incubated with FITC conjugated anti-tubulin (Abcam, ab64503) for 1 h before detection. FITC/FL1 channel (488 nm blue laser/ 533/30 nm band-pass filter) was used for detection.

### Alamar Blue cell viability assay

0.1 × 10⁶ cells were seeded in 96-well plates and subjected to treatments and incubation conditions as described in the corresponding figure legends. 11 µl of Alamar blue 10 x solution (Fisher Scientific, Waltham MA, A50100) was added to each 100 µl cell suspension/well of a 96-well plate and incubated for 4 h. The fluorescence intensity increase was detected by using a BioTek Synergy2 microplate reader (Thermo Fisher Scientific).

### Confocal microscopy

5 × 10⁵ cells per well were seeded in a 12-well plate and treated as indicated in the figure legends. After incubation time, the cells were cytocentrifuged onto Colorfrost plus slides (Fisher Scientific, 1255020) using a Cytospin 3 centrifuge (Shandon, Abbey Ward, UK). Slides were fixed in 4% paraformaldehyde (PFA) for 10 min, followed by three washes with PBS. Cells were permeabilized with PBS with 0.1% Triton X-100 for 10 min at RT and subsequently blocked with 10% goat serum for 30 min. Next, slides were washed with PBS 3 x and incubated with primary antibody (1:100 dilution in PBS with 1% BSA) at 4 °C hour in the dark overnight. The following day, cells were washed and incubated for 1 h at room temperature with Alexa 488 conjugated goat anti-rabbit IgG (ABCAM, AB150077) and/or Alexa 555 conjugated goat anti-mouse IgG (Cell Signaling Technology, 4409S) secondary antibodies, both prepared at 1:100 dilution. Next, samples were washed in PBS 3X and incubated with Biotracker 655 cytoplasmic membrane stain (Millipore Sigma, SCT108) for 30 min. Following three PBS washes, cells were mounted with antifade mounting medium and covered by coverslips. The following dyes and primary antibodies were used: Anti-EEA1 (Cell Signaling Technology, 3288 T), Anti-CD81 (ABCAM, AB59477), Anti-N-terminal GSDMD (ABCAM, AB255983). Images were acquired using a Leica Stellaris 5 (Leica, Wetzlar, Germany) confocal microscope equipped with 405, 488, 514, 559, and 638 nm laser lines. High-resolution imaging was achieved through pinhole-based optical sectioning using the 63x oil immersion objective. Laser intensity and gain values were kept constant throughout the samples within the same experiment. The image processing was accomplished by *Leica LAS X Office* (Leica) and by *Fiji* (http://imagej.org). In case the minimum and maximum intensity thresholds were adjusted, they were kept constant throughout all the samples and were indicated at the corresponding figure legend.

### Statistical analysis

Statistical significance was calculated by Student’s t-test (two tailed) and by one-way ANOVA for multiple comparisons using *GraphPad Prism 10* software. All experiments were repeated at least three times. Error bars indicate SD of the mean.

## Results

### Nigericin without LPS and alpha toxin induces GSDMD-dependent pyroptosis

**Fig. 1:**
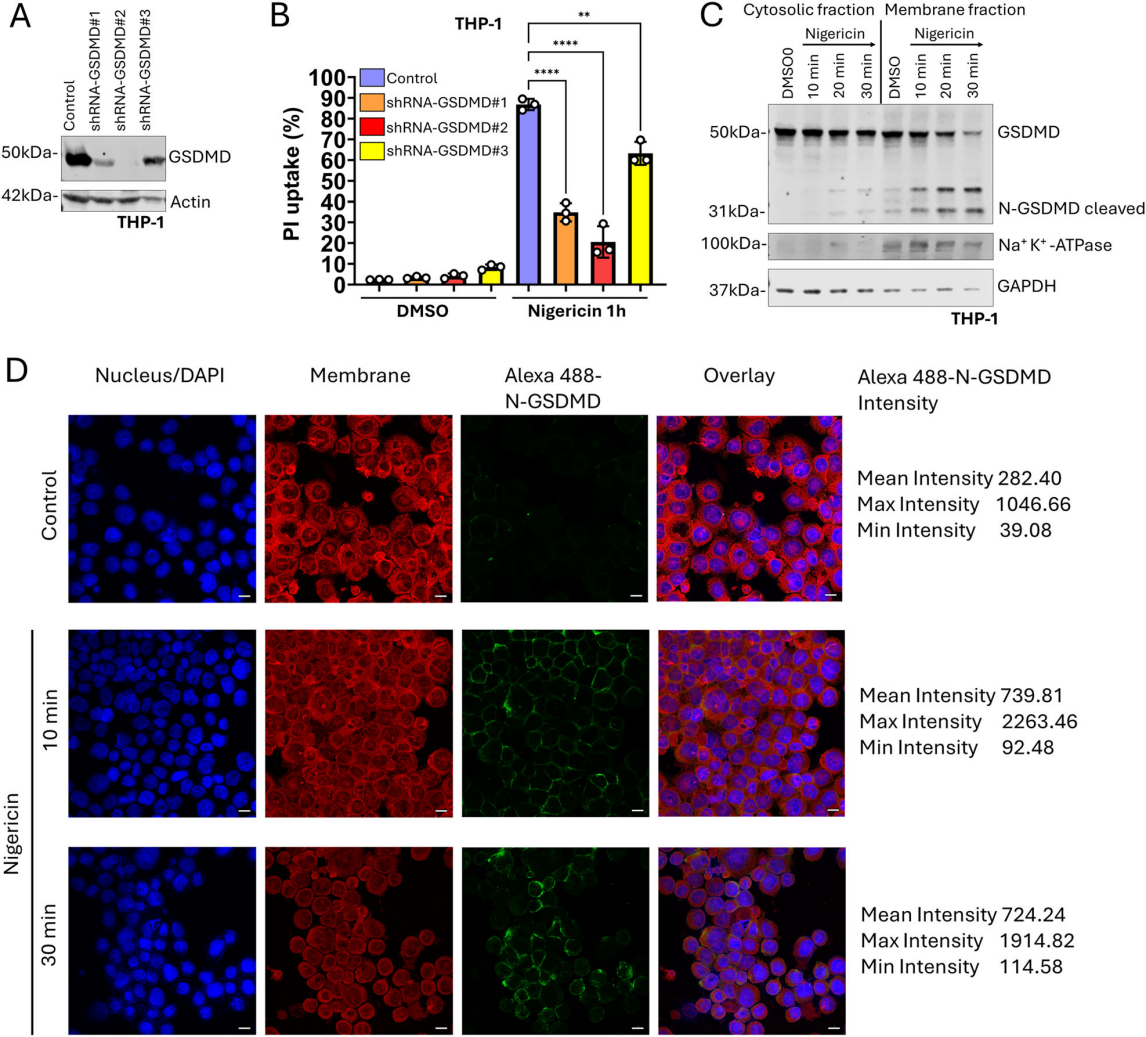
N-GSDMD is essential for nigericin-induced pyroptosis. **A** Immunoblot GSDMD of control and shRNA-GSDMD#1, 2, and 3 knockdown THP-1 cells. Actin was used as internal loading control. **B** Flow cytometry analysis of THP-1 control and shRNA-GSDMD cells at 1 h after treatment with 10 µM nigericin. The y-axis shows the percentage of cells with increased PI fluorescence intensity (PI uptake). *n* = 3. Significance was tested with one-way ANOVA, p** ≤ 0.01; p**** ≤ 0.0001. **C** Immunoblot of full length GSDMD and cleaved N-terminal GSDMD of cytoplasmic and membrane fraction of THP-1 cells after treatment with 20 µM nigericin at the indicated time points. Na + /k+ ATPase was used as marker of the membrane fraction. GAPDH was used as internal loading control. **D** Representative confocal microscopy images of THP-1 cells 10 and 30 min after 20 µM nigericin treatment. DAPI = Nucleus, Membrane = Biotracker 655 cytoplasmic membrane stain, Alexa 488 = cleaved N-GSDMD. The images show the maximal projection. Scale bar = 10 μM. Intensity values show the Alexa 488 channel.

To stimulate pyroptosis, we initiated potassium efflux, a common trigger of pyroptosis [21]. In THP-1 monocytic cell lines, we pre-treated cells with either LPS and the potassium ionophore nigericin in combination or with nigericin alone. Intriguingly, cells treated with only nigericin without LPS exhibited GSDMD cleavage into N-GSDMD in both THP-1 and HCT-116 cells (Supplementary Fig. 1A, B). In addition, we employed alpha toxin from *Staphylococcus aureus* to induce potassium efflux and pyroptosis. Alpha toxin treatment resulted in GSDMD cleavage in both THP-1 and HCT-116 cells (Supplementary Fig. 1C and Fig. 5A). Finally, substantial enrichment of N-GSDMD along the cell membrane was observed 10 to 30 min post nigericin treatment detected by confocal microscopy (Fig. 1D). To ensure that the stimulated cell death by potassium efflux is GSDMD dependent pyroptosis, we generated stable shRNA-GSDMD knockdown cell lines by using lentiviral transduction particles (Fig. 1A). The cell death reduction compared to control cells was proportional with the efficiency of the knockdown, with the most efficient knockdown showing the strongest cell death inhibition (Fig. 1B). This indicates that most of the cell death stimulated by potassium efflux was GSDMD-dependent pyroptosis.

### Gasdermin-D membrane pores induced membrane permeability is reversed in pyroptosis

**Fig. 2:**
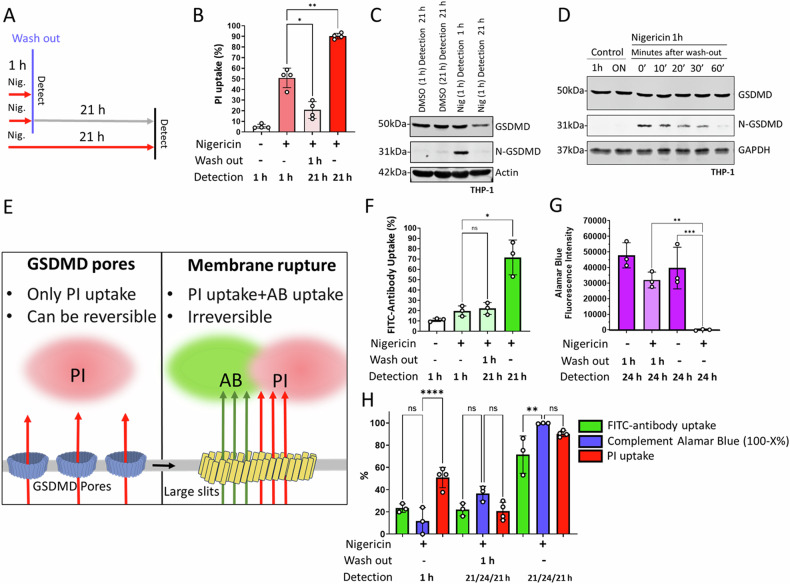
N-GSDMD dependent membrane permeability is reversible in nigericin induced pyroptosis. **A** THP-1 cells were treated with nigericin (Nig). Wash-out was performed at 1 h post nigericin treatment where indicated. Cells were washed by centrifugation and fresh media was added. The cells were placed back in the incubator until detection. Time of detection was either 1 h or 21 h post nigericin treatment. **B** PI uptake (%) by THP-1 cells was detected by flow cytometry 1 h or 21 h after treatment with 40 μM nigericin for 1 h or 21 h as shown in (**A**). Significance was determined with one-way ANOVA, ** = *p* ≤ 0.01; * =*p* ≤ 0.05, *n* = 4. **C** Immunoblot of THP-1 cells harvested 1 h or 21 h after treatment with nigericin. Cells were treated with nigericin for 1 h and then either harvested or washed out and placed back in the incubator for another 20 h. Detection of GSDMD, N-GSDMD, and Actin levels. **D** Immunoblot of THP-1 cells harvested at different time points after wash-out of nigericin treatment at 1 h. Detection of GSDMD, N-GSDMD, and GAPDH levels. **E** Membrane rupture was distinguished from membrane permeability by employing co-stain with propidium iodide (PI) and FITC-tagged antibody (AB). **F** FITC-antibody uptake (%) of THP-1 cells detected at 1 h or 21 h post treatment with 40 μM nigericin. Significance was determined with one-way ANOVA, ns=non-significant; * = *p* ≤ 0.05, *n* = 3. **G** Alamar Blue fluorescence intensity (cell viability) detection in THP-1 cells treated with 25 µM nigericin. Nigericin was washed out at 1 h post treatment where indicated. Samples were stained with Alamar Blue at 20 h post treatment, and the fluorescence intensity was detected at 24 h post treatment. Alamar blue fluorescence intensity values are shown. Cell-free media was used as negative control. The fluorescence values of cell-free media were subtracted from the values presented in cell viability diagram. Significance was determined with one-way ANOVA, ns = non-significant; *** = *p* ≤ 0.005, *n* = 3. **H** Comparison of PI uptake, FITC-Antibody uptake, and Alamar Blue. Cell-free media was used as negative control. The fluorescence values of cell-free media were subtracted from the values presented in cell viability diagram. For 1 h Alamar blue samples, nigericin was washed out at 1 h and incubated with Alamar Blue for 4 h. Data were shown as complement fluorescence intensity values (100-X%), where X represents the percentage of Alamar Blue intensity relative to the untreated control samples (100%). This value represents the percentage of non-viable fractions of the sample to enable comparison with PI uptake and FITC-antibody uptake. The PI uptake shows values from experiments in (**B**). The FITC-antibody uptake shows values from experiments in (**F**). Significance was determined with one-way ANOVA. ns = non-significant; **=*p* ≤ 0.01; ****=*p* ≤ 0.0001.

By employing subcellular fractionation from THP-1 cell lysates, nigericin treatment alone resulted in GSDMD cleavage and led to the translocation of N-GSDMD from the cytoplasm to the cell membrane within 10 min (Fig. 1C). We set out to answer the question of whether membrane N-GSDMD pore formation is irreversible and always lead to cell death, or the pores can be removed within a certain period without leading to cell death. To establish a cell model where we can investigate N-GSDMD removal, we incubated cells with nigericin for 1 h and then removed the nigericin by repeated washing of the cells and we also incubated cells with nigericin continuously for 21 h (Fig. 2A). GSDMD cleavage, propidium iodide (PI), and 7-AAD uptake, as the indirect measures of N-GSDMD pores in the membrane, were detected 1 h post-induction and were maximal after 21 h of incubation with continuous nigericin (Fig. 2B and Supplementary Figure 2). In the cells where we washed out the stimulus after 1 h, the proportion of cells exhibiting membrane permeability was significantly less at 21 h (Nig 1 h, detection 21 h) compared to the 1 h time point (Nig 1 h, detection 1 h) (Fig. 2B and Supplementary Fig. 2). Consistent with this observation, the level of N-GSDMD was reduced to control levels in the 21 h sample. The N-GSDMD level was already substantially reduced within 1 h after nigericin wash-out (Fig. 2C, D). To exclude the possibility that the reduced N-GSDMD protein level and decreasing membrane permeability are due to the significant loss of structurally intact cells over time, we showed the actin level in the samples, and it remained constant between 1 h and 21 h (Fig. 2C). These experiments provide evidence for an active membrane repair mechanism that counters the formation of N-GSDMD pores and restores membrane integrity.

### Membrane permeability is reversible, whereas large-scale membrane rupture is irreversible

To further test the hypothesis that GSDMD pores do not equate to terminal membrane rupture, we developed a flow cytometry-based quantitative double-staining method for membrane pore size discrimination. This method simultaneously uses PI to detect small-scale membrane permeability and anti-tubulin antibody staining to identify large-scale membrane rupture (Fig. 2E). PI would be taken up through GSDMD pores, but the pores would be too small to take up the antibody (Fig. 2E). When we treated THP-1 cells with nigericin, PI uptake was detected within 1 h in ~50% of the cells. In contrast, most cells did not take up the anti-tubulin antibody until after 21 h of treatment. When the nigericin was removed after 1 h, the antibody uptake remained close-to the control level until 21 h (Fig. 2F). To validate that the large-scale membrane rupture observed represents a terminal event, we employed Alamar Blue cell viability detection, which does not rely on cell permeability parameters. This data shows that early-stage membrane permeability is restored, and cells remain viable (Fig. 2G, H) up to 21 h after nigericin treatment (in 1 h washed out samples), whereas cells that take up the FITC-anti-tubulin antibody (21 h continuous nigericin) lose their viability at 21 h post-treatment (Figure G and H). This data confirms that the extent of anti-tubulin antibody uptake is in direct correlation with the loss of cell viability. Additionally, these experiments show that early-stage GSDMD pore formation and membrane permeability detected by PI (t$\le \,$ h) is not lethal, and that it can be reversed before it progresses to pyroptosis.

### A subpopulation of cells is resistant to pyroptosis within a homogeneous cell population

**Fig. 3:**
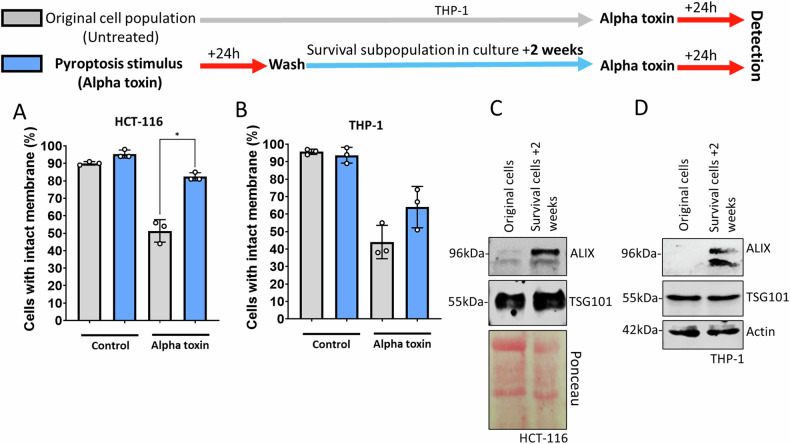
Pyroptosis resistant cells maintain their ability to withstand a second pyroptotic stimulus two weeks after the first stimulus. **A** HCT-116 original population and alpha toxin treated survival cell population were further treated with 1500 ng/ml alpha toxin 2 weeks post stimulus. The y-axis shows the percentage of the cells not taking up trypan blue. Cells were counted using the Bio-Rad TC-20 automated cell counter (BIORAD, Hercules, CA, USA, 1450102) following staining with trypan blue dye to distinguish viable from non-viable cells. A 10 µL mixture of cell suspension and 0.4% trypan blue (1:1 ratio) was loaded into a counting slide and analyzed according to the manufacturer’s instructions. **B** THP-1 original population and alpha toxin treated survival cell population were further treated with 1500 ng/ml alpha toxin 2 weeks later. The y-axis shows the percentage of the cells not taking up trypan blue detected by automated cell counter. **C** Immunoblot of ALIX level and TSG101 level in HCT-116 original and survival cells. **D** Immunoblot of ALIX level and TSG101 level in THP-1 original and survival cells.

Our data demonstrates that even in seemingly homogeneous cell populations, a substantial number (~20%) of cells survive in response to pyroptotic inducers (Figs. 1B, 2F). This result suggests that there are long term genetic or expressional differences between cells that determine sensitivity to pyroptosis. To explore this idea further, we incubated THP-1 and HCT-116 cells with alpha toxin for 1 h and, after removing the stimulus, kept the cells in culture (Fig. 3). After two weeks, the surviving cells preserved their ability to proliferate, indicating their long-term viability. When we re-exposed this surviving cell population to alpha toxin, it exhibited increased resistance to pyroptotic stimulus compared to the control unselected cell population (Fig. 3A, B). This demonstrates that the resistance to pyroptosis is maintained long term. We propose that the improved ability of these cells to survive is dependent on permanent genetic or gene expression differences.

### ALIX reverses membrane permeability and facilitates N-GSDMD removal

**Fig. 4:**
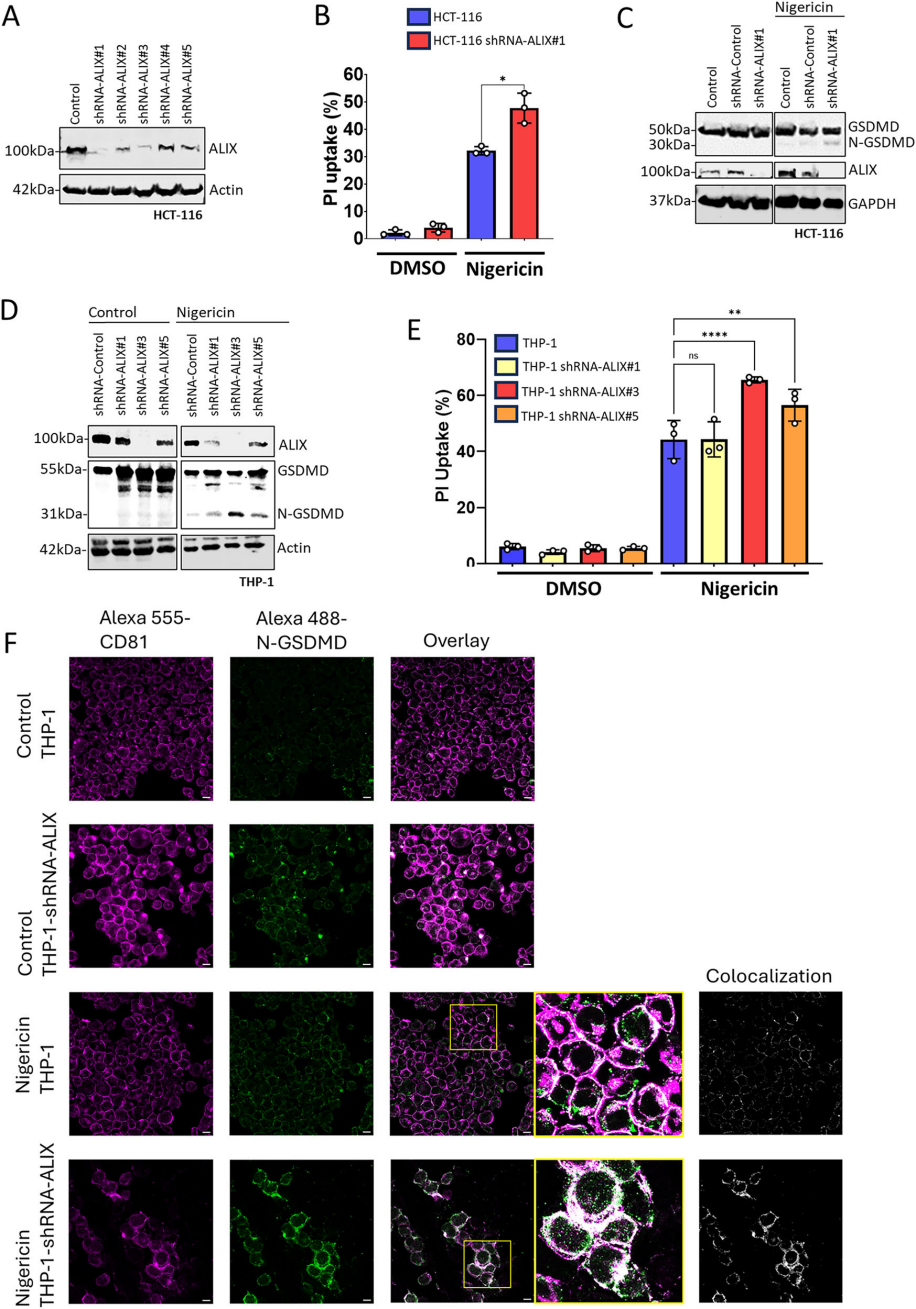
ALIX knock down increases pyroptosis susceptibility and N-GSDMD level. **A** Immunoblot of HCT-116 and HCT-116 shRNA-ALIX#1, 2, 3, 4, and 5 cells. **B** Flow cytometry detection of HCT-116 and shRNA ALIX#1 cells at 24 h after treatment with 20 µM nigericin. The y-axis shows the percentage of cells with high PI fluorescence intensity (PI uptake). *n* = 3. To determine the significance, Student’s t test was used. *=*p* ≤ 0.05. **C** Immunoblot of HCT-116 control, shRNA-control, and shRNA ALIX#1 cells 24 h. after treatment with 20 µM nigericin. **D** Immunoblot of THP-1 and THP-1 shRNA-ALIX#1, 2, and 3 cells at 1 h after treatment with 20 µM nigericin. **E** Flow cytometry detection of THP-1 and shRNA ALIX#1 cells at 24 h after treatment with 20 µM nigericin. The y-axis shows the percentage of cells with high PI fluorescence intensity (PI uptake). *n* = 3. To determine the significance, the Student’s t test was used. *p* * ≤0.05. **F** Representative confocal microscopy images showing THP-1 and THP-1 shRNA-ALIX# 3 cells 1 h after treatment with 20 µM nigericin. Anti-C81-Alexa-555 (magenta), Anti-N-GSDMD-Alexa-488 (green) are shown. White demonstrates colocalization of CD-81 and N-GSDMD. A single z-plane is presented. Scale bar = 10 µM.

**Fig. 5:**
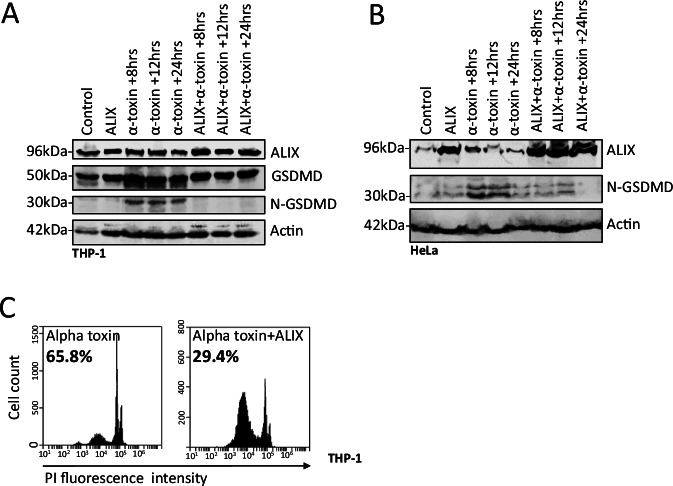
ALIX overexpression reduces N-GSDMD level and pyroptosis susceptibility. **A** Immunoblot of ALIX overexpressing THP-1 cells treated with 500 ng/ml alpha toxin at time points 8,12, and 24 h. **B** Immunoblot of ALIX overexpressing HeLa cells treated with 500 ng/ml alpha toxin at time points 8,12, and 24 h. Actin was used as internal loading control. **C** Flow cytometry detection of ALIX overexpressing THP-1 cells 1 h after 500 ng/ml alpha toxin treatment. Percentages indicate the cells showing high PI intensity (PI uptake).

To identify some of these genetic differences, we focused on the ESCRT components ALIX and TSG101, since these proteins play multiple roles in various ESCRT dependent functions [15, 16, 19, 20]. Thus, we interrogated the expression of ALIX and TSG101 in the pyroptosis resistant survival cell population compared to the original cell population (Fig. 3A, B). Immunoblot analysis revealed a strong increase in ALIX level in the pyroptosis resistant cells compared to the original cells, however, TSG101 level remained constant (Fig. 3C, D). To determine if ALIX is required for removing N-GSDMD, we generated stable ALIX knockdown THP-1 and HCT-116 cell lines (Fig. 4A, D). We used stable knockdowns instead of CRISPR/Cas9-driven gene deletion since ALIX is essential in cell division process [20] and the knockdowns already showed a substantially slower proliferation rate in our experiment (not shown). ALIX knockdown cells were significantly more susceptible to cell death induced by various pyroptosis inducers (Fig. 4B, E and Supplementary Fig. 3A), exhibited higher levels of N-GSDMD (Fig. 4C, D), and elevated N-GSDMD/membrane (CD81) [22] association (Fig. 4F) compared to control cells following nigericin treatment. Cell death initiated by apoptosis inducer staurosporine was not affected by ALIX knock down (Supplementary Fig. 3B, C). Conversely, overexpression of ALIX reduced N-GSDMD levels (Fig. 5A, B) and decreased the cell death rate in alpha toxin-treated THP-1 cells (Fig. 5C).

### TSG101, the binding partner of ALIX is not required for regulating pyroptosis resistance

**Fig. 6:**
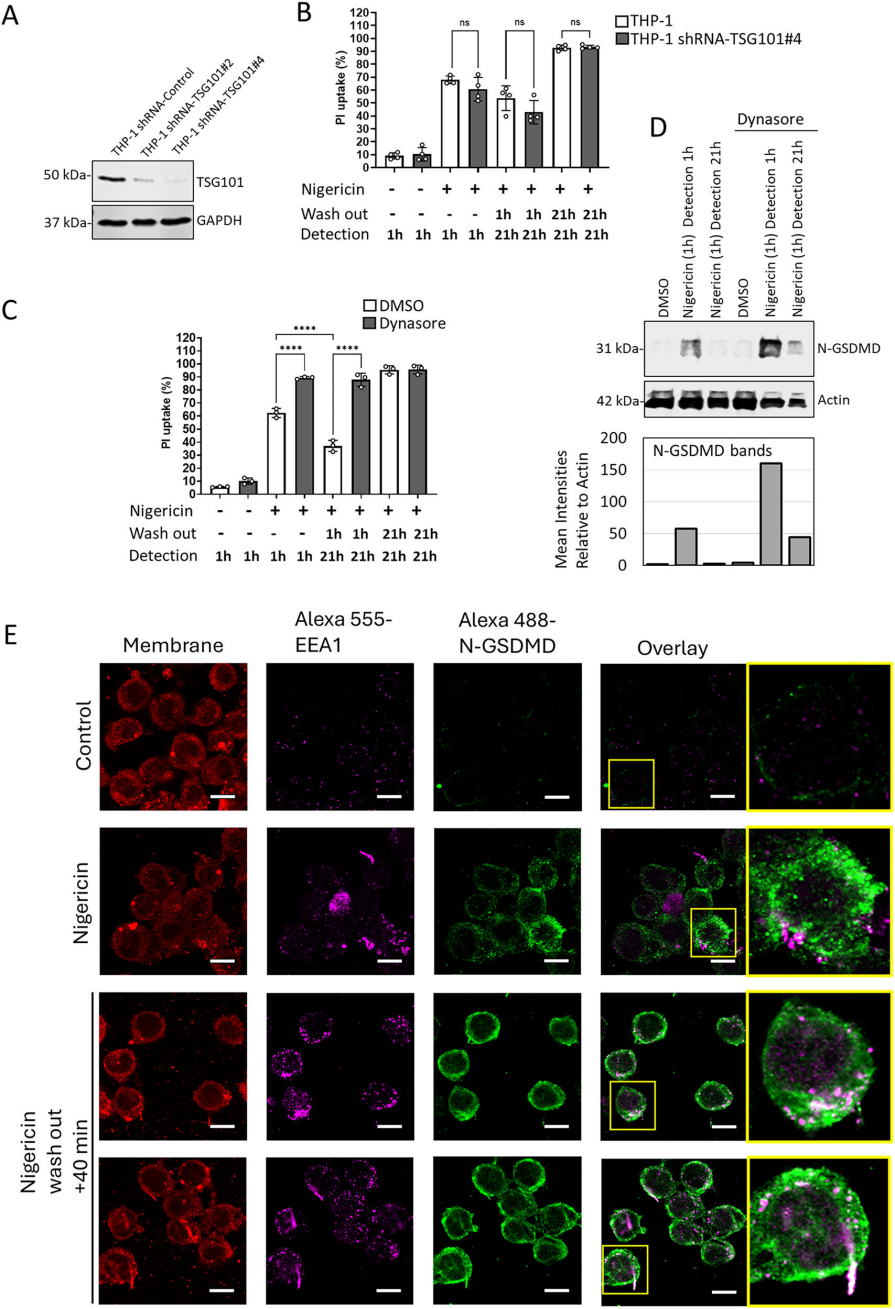
TSG-101 knock down does not affect pyroptosis susceptibility, whereas dynamin inhibition increases pyroptosis susceptibility and blocks membrane repair. **A** Immunoblot of control and shRNA-TSG101#2 and #4 knockdown THP-1 cells. **B** Flow cytometry detection of control and shRNA TSG101#4 cells at 1 h and 21 h after treatment with 40 µM nigericin with and without wash-out at 1 h. The y-axis shows the percentage of cells with high PI fluorescence intensity (PI uptake). *N* = 4. Significance was tested with one-way ANOVA, ns=non-significant; p**** ≤ 0.0001. **C** Flow cytometry detection of control and 80 µM dynasore pretreated (−30 min) cells at 1 h and 21 h after treatment with 40 µM nigericin with and without wash-out at 1 h. The y-axis shows the percentage of cells with high PI fluorescence intensity (PI uptake). *n* = 4. Significance was tested with one-way ANOVA, ns = non-significant; p**** ≤ 0.0001. **D** Immunoblot of control and 80 µM dynasore pretreated (-30 min) THP-1 cells at 1 h and 21 h post treatment with 40 µM nigericin. Nigericin was washed out at 1 h. Actin was used as internal loading control. The N-GSDMD signal intensities were quantified by using *Image J* software. The mean intensity of the bands was normalized to the mean intensity of the actin bands. The background intensity was subtracted from the values. **E** Representative confocal microscopy images showing THP-1 cells 1 h after treatment with 20 µM nigericin and 40 min after 20 µM nigericin (1 h) wash out. EEA1-Alexa-555 (magenta), N-GSDMD-Alexa-488 (green) are shown. White demonstrates colocalization of EEA1 and N-GSDMD. A single z-plane is presented. In Alexa 488 channels, minimum threshold set to 7000; in Alexa 555, minimum threshold set to 3000, maximum threshold set to 30,000. Scale bar = 10 µM.

ALIX plays a role in certain viral budding processes [19] in which TSG101 works as essential binding partner of ALIX. We first hypothesized that the removal of N-GSDMD from the membrane acts analogously to a viral budding process, where the viral envelope protein is released with the corresponding host membrane section. To test this hypothesis, we generated TSG101 stable knockdown cells (Fig. 6A). Unlike ALIX knockdowns, TSG101 knockdowns did not have a significant effect on membrane permeability and did not inhibit the membrane integrity restoration, similarly to the control cells (Fig. 6B). This data shows that ALIX acts independent of TSG101 in promoting N-GSDMD removal and membrane repair.

### Endosomal route dependent N-GSDMD internalization plays a role in N-GSDMD removal

**Fig. 7:**
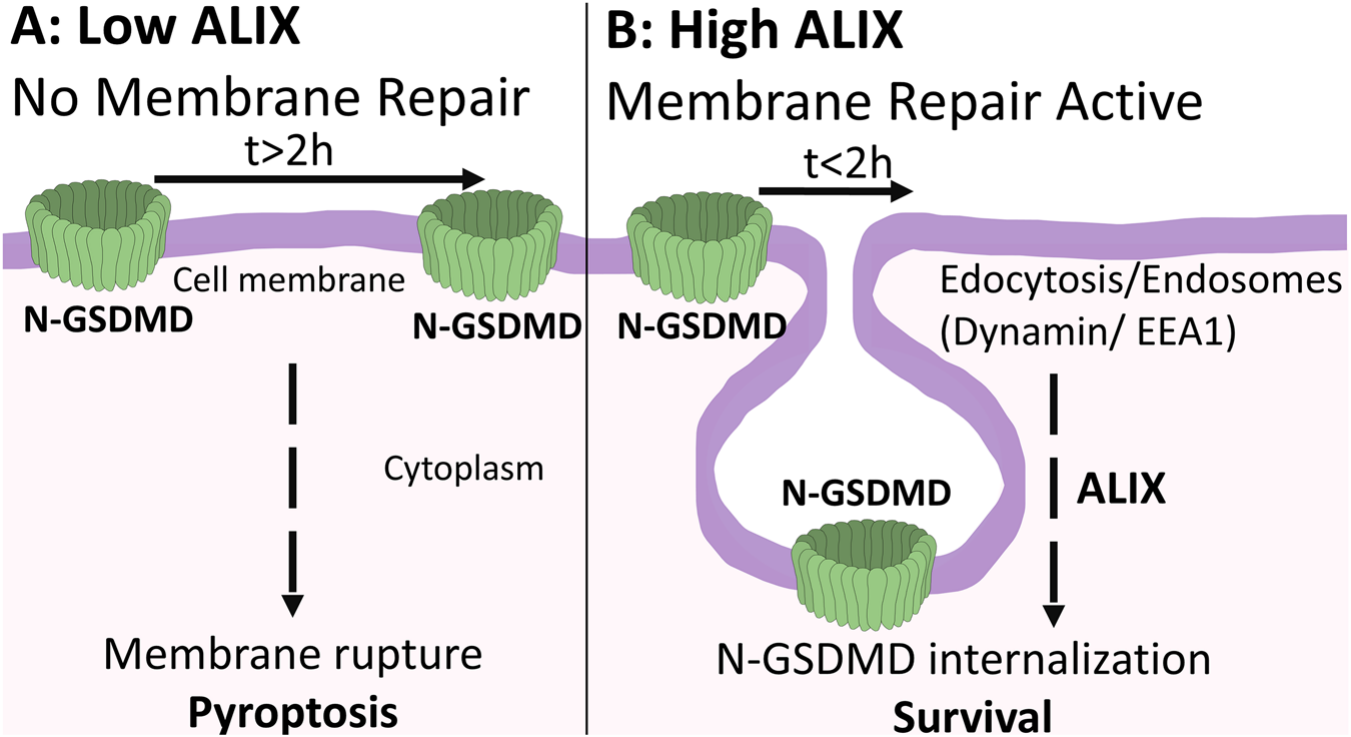
N-GSDMD internalization protects from pyroptosis in an endosomal and ALIX dependent way. **A** Low ALIX level facilitates the increase of N-GSDMD level and long-term membrane permeability leading to membrane rupture and pyroptosis. **B** High ALIX level promotes removal of N-GSDMD in endosomal pathway- and ALIX-dependent internalization leading to restoring membrane integrity and survival.

To explore the possibility of internalization and endocytosis as a mechanism of membrane N-GSDMD removal, we used dynasore, a potent inhibitor of dynamin [23], which plays an essential role in the first step of endocytic vesicle formation and the endosomal pathway [24]. Dynasore significantly increased the membrane permeability rate 1 h after nigericin treatment and entirely blocked the membrane permeability restoration 21 h after nigericin wash-out as compared to control cells (Fig. 6C). Dynasore treatment resulted in higher endogenous level of N-GSDMD (Fig. 6D). Additionally, nigericin-treated cells showed increased levels of the early endosomal marker EEA1 [25], with enhanced colocalization between EEA1 and N-GSDMD following nigericin wash out (Fig. 6E). These findings support the conclusion that N-GSDMD clearance and membrane integrity restoration are predominantly mediated through the endocytic/endosomal pathway. (Fig. 7).

## Discussion

In this study, we show that the initial membrane permeability is reversible in pyroptosis and is likely dependent on removal of N-GSDMD via the endosomal pathway (Fig. 7).

Initial studies suggested that IL-1β release exclusively occurs in dying cells showing terminal necrosis [26]. Since then, it was shown that IL-1β can be released from living macrophages [10, 11]. It is now evident that the formation of GSDMD pores alone on the cytoplasmic membrane does not always lead to cell death. However, the link between GSDMD pore formation and large-scale membrane rupture and cell death remains unclear [27]. In this study, we provide evidence that membrane permeability detected by PI staining is not equal to cell death and can be reversed if the pyroptosis stimulus is removed. In addition, we show that the level of ALIX significantly affects membrane permeability, cell membrane N-GSDMD level, and cell viability but does not influence IL-1β release (Supplementary Fig. 4A–D).

It was previously shown [13] that N-GSDMD is removed in the form of exosomes in an ESCRTIII-dependent process. It was proposed that the executioner part of ESCRT complex, ESCRTIII, including charged multivesicular body protein (CHMP)-4 and -3, translocate to the plasma membrane during pyroptosis to promote the removal of GSDMD-containing membranes in the form of exosomes [13]. This study used ectopically expressed CHMPs to show the membrane translocation of the ESCRTIII in mouse BMDM, HeLa, and HEK293T cells. This suggested that ESCRT machinery is a potential mechanism to remove GSDMD pores. However, the existence of such a membrane GSDMD pore repair mechanism in human inflammatory diseases and their target cells, like intestinal epithelial cells, monocytes, and macrophages is yet to be determined. In addition, the N-GSDMD dependent mechanism that initiates the ESCRTIII complex has not yet been investigated. To follow up this finding, our study shows strong evidence that the N-GSDMD is likely removed via the endosomal pathway and ALIX dependent process in human THP-1 and HCT-116 cells. In our experimental model, TSG101 [16] did not impact N-GSDMD clearance, and no detectable association was found between extracellular CD81, a marker of exosomes [22], and N-GSDMD. These findings suggest that N-GSDMD internalization or its direct shedding into exosomes may be dependent on cell type or specific cellular context.

Dynamin is an essential component of the first step of endosomal pathway by executing the pinching off the vesicles from the cytoplasmic membrane [24]. Our dynamin inhibitor experiments along with EEA1 colocalization data suggest that N-GSDMD is internalized by utilizing the endosomal pathway. ALIX plays a role in MVB formation, the mechanism of endocytic vesicle packaging into endosomes [28, 29]. The vesicles in MVB can continue their route in two directions, either fuse with the plasma membrane and be released as exosomes [30] or fuse with lysosomes and lead to the degradation of the cargo [31]. Given the lack of substantial colocalization between N-GSDMD and exosomal marker in our imaging analysis, degradation via the endosomal pathway appears to be the most likely mechanism of N-GSDMD clearance; however, further investigation is needed to confirm this.

We showed that ALIX level is a critical factor in controlling pyroptosis susceptibility. Along with this, in clinical studies, higher ALIX levels in intestinal mucosa have been associated with better prognosis and non-relapsing disease in patients with ulcerative colitis (UC) [32]. This indicates that higher ALIX level in UC patients facilitates N-GSDMD removal and reduce pyroptosis susceptibility. Elevating or protecting ALIX function and the associated components in pyroptosis can lead to the development of novel therapeutic interventions to improve tissue functions in chronic inflammatory diseases.

## Supplementary information

Supplemental Methods

Supplementary Figure 1

Supplementary Figure 2

Supplementary Figure 3

Supplementary figure 4

Full Western Blots

Flow Cytometry Histograms

## Data Availability

The datasets generated and/or analyzed during the current study are available from the corresponding author on reasonable request.
