# Supplementary material for: ALIX mediates reversible gasdermin-D pore formation via the endosomal pathway to limit pyroptosis by active membrane repair
Source: Cell Death Dis. 2025 Oct 6;16(1):681. doi: 10.1038/s41419-025-07998-y (PMC12501301; doi:10.1038/s41419-025-07998-y)
Supplement: Supplementary file 1 — Supplemental Methods [file 41419_2025_7998_MOESM1_ESM.docx]

**Supplemental Materials and Methods**

**Reagents**

lipopolysaccharide (LPS) (S7850) and staurosporine (S1421) were obtained from Selleckchem (Houston, Texas, USA) and prepared by dissolving in dimethyl sulfoxide (DMSO).

**Subcellular fractionation**

*ProteoExtract* subcellular proteome extraction kit was employed as per instructions (Calbiochem, 539790). Briefly, 3x10^6^ cells were treated with nigericin (20 μM) for indicated time and harvested by centrifugation at 1 100 rpm, 4 min. Cell pellets were washed with an ice-cold wash buffer and mixed with extraction buffers (I-II) (provided in the kit) to obtain protein fractions (cytosolic, and membrane). Each fraction was resuspended in Laemmli 2 x concentrate, boiled for 5 min at 99^o^C, and subjected to SDS-PAGE.

**Plasmid transfection**

Cells were cultured in 6- or 12-well plates until 70-80 % confluent or reached. The media was replaced with DMEM. 100 μl/ml DMEM was mixed with GeneJuice transfection reagent (ratio: 1 μg DNA: 3 μl GeneJuice) (Millipore Sigma, 70967) and incubated at room temperature for 5 min. Then, 1 μg/ml plasmid was added, mixed, and incubated at room temperature for 15 min before adding the freshly prepared solution dropwise to the cells. The cells were incubated in 5 % CO_2_ at 37^o^C for at least 4 h before replacing the media with a complete growth media. After overnight incubation, cells were treated for indicated time before harvesting them for further experiments. The following plasmid was employed: pcDNA3-ALIX-Flag (797220) (Addgene, Watertown, MA, USA)

**Generation of stable knockdown cell lines by lentiviral transduction**

To knock down the cells with shRNA lentiviral particles, 400 000 cells/1 ml were seeded in a 12-well plate. Ready-to-use viral particles (Millipore Sigma) (MOI=5.0) and 8 μg/ml polybrene transfection reagent (Millipore Sigma, TR-1003-G) were added to the cells the following day (HCT-116) or 1 h after seeding (THP-1). At 24 h post transfection, the media was replaced with growth media containing puromycin (Millipore Sigma, P4512) for the selection. When cells reached 90-100 % confluency (HCT-116) or 1.5 million cells/ml cell density (THP-1), they were transferred to a 6-well plate. Cells were cultured further in the presence of puromycin to achieve positive selection. To validate the knockdown efficiency, immunoblot was used.

The following shRNA transduction particles were used (Millipore Sigma): ShControl (SHC001VN): non-target shRNA sequence

● GSDMD #1: TRCN0000179394 CDS

Target sequence: GTGTGTCAACCTGTCTATCAA

● GSDMD #2: TRCN0000180013 3UTR

Target sequence: CCTTCTCTTCCCGGATAAGAA

● GSDMD #3: TRCN0000179101 CDS

Target sequence: CAGCACCTCAATGAATGTGTA

● ALIX (PDCD61P) #1: TRCN0000034359326

Target sequence: GCAGAACAGAACCTGGATAAT

● ALIX (PDCD61P) #3: TRCN00000343595 CDS

Target sequence: CCTGAATTACTGCAACGAAAT

● ALIX (PDCD61P) #5: TRCN00000382397 3UTR

Target sequence: GCATAATCAAGGCACTGTAAA

**SubG1 detection by flow cytometry**

Cells (0.5 × 10⁶) were harvested into microcentrifuge tubes, including floating cells from the culture medium to ensure collection of apoptotic populations. After centrifugation at 300 × g for 2 min, the supernatant was discarded, and the cell pellet was resuspended in 1 ml of ice-cold 70 % ethanol and incubated at room temperature for 25 min. Fixed cells were stored at −20 °C for a minimum overnight and up to two weeks before analysis. Prior to staining, samples were centrifuged at 300 × g for 3 min and washed thoroughly. The pellet was then resuspended in 500 μL extraction buffer (200 mM Na₂HPO₄, pH adjusted to 7.8 with 1 M citrate) supplemented with RNase A (10 μg/mL), followed by incubation at room temperature for 15 min. Subsequently, PI was added to a final concentration of 5 μg/mL, and samples were incubated in the dark for at least 15 min before flow cytometric analysis. PI fluorescence was analyzed using a BD Accuri C6 Plus flow cytometer (BD), using the FL2 channel (488 nm blue laser/ 585/40 nm band‐pass filter). Cell debris (population exhibiting low FSC/FL2 intensity) were excluded from the analysis in FSC/FL2 dot-plot. Cells with low (Sub-G1) DNA content were quantified as apoptotic.

**Human IL-1β ELISA**

Cells were subjected to treatments and incubation conditions as described in the corresponding figure legends. After treatment, IL-1β levels in cell culture supernatants were quantified using a commercial ELISA kit (Millipore Sigma, RAB0273) following the manufacturer’s instructions. Standards and samples were added in duplicate (100 µl per well) to pre-labeled 96-well strip plates and incubated for 2.5 h at room temperature with gentle shaking. Wells were then washed four times with 1 x wash buffer, ensuring complete removal of liquid between steps. Subsequently, 100 μl of 1x biotinylated detection antibody was added to each well and incubated for 1 h at room temperature with gentle shaking, followed by another wash step. Streptavidin–HRP solution (100 μl) was then added and incubated for 45 min under the same conditions, followed by a final series of washes. Signal development was achieved by adding 100 µl of TMB substrate to each well and incubating for 30 min in the dark. The reaction was stopped by adding 50 µl of Stop Solution, and absorbance was measured immediately at 450 nm using a microplate reader. Mean absorbance values were calculated for all duplicate standards, controls, and samples after subtracting the average zero standard reading. A standard curve was generated by plotting absorbance against concentration on a log-log scale using *GraphPad Prism 10* software (Boston, MA, USA), and a best-fit line was applied to determine sample concentrations.
