# Supplementary figures and images for: ALIX mediates reversible gasdermin-D pore formation via the endosomal pathway to limit pyroptosis by active membrane repair
Source: Cell Death Dis. 2025 Oct 6;16(1):681. doi: 10.1038/s41419-025-07998-y (PMC12501301; doi:10.1038/s41419-025-07998-y)

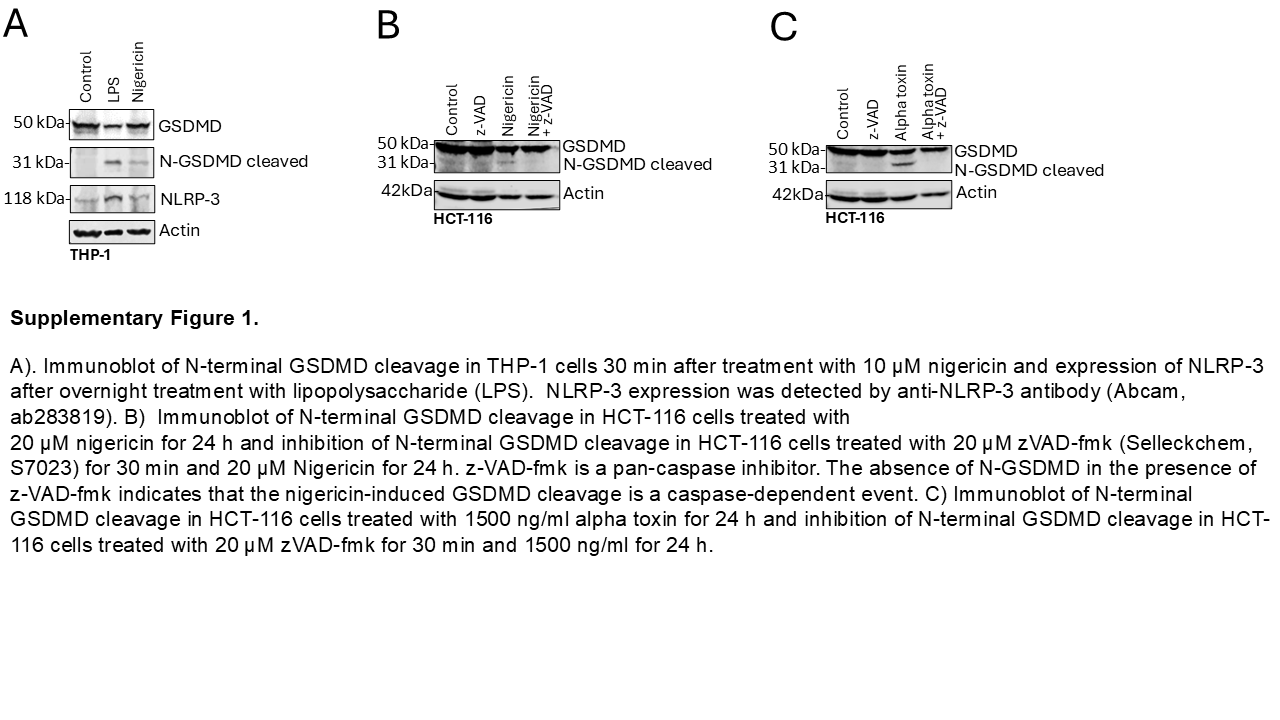

Supplement: Supplementary file 2 — Supplementary Figure 1 [file 41419_2025_7998_MOESM2_ESM.tif]

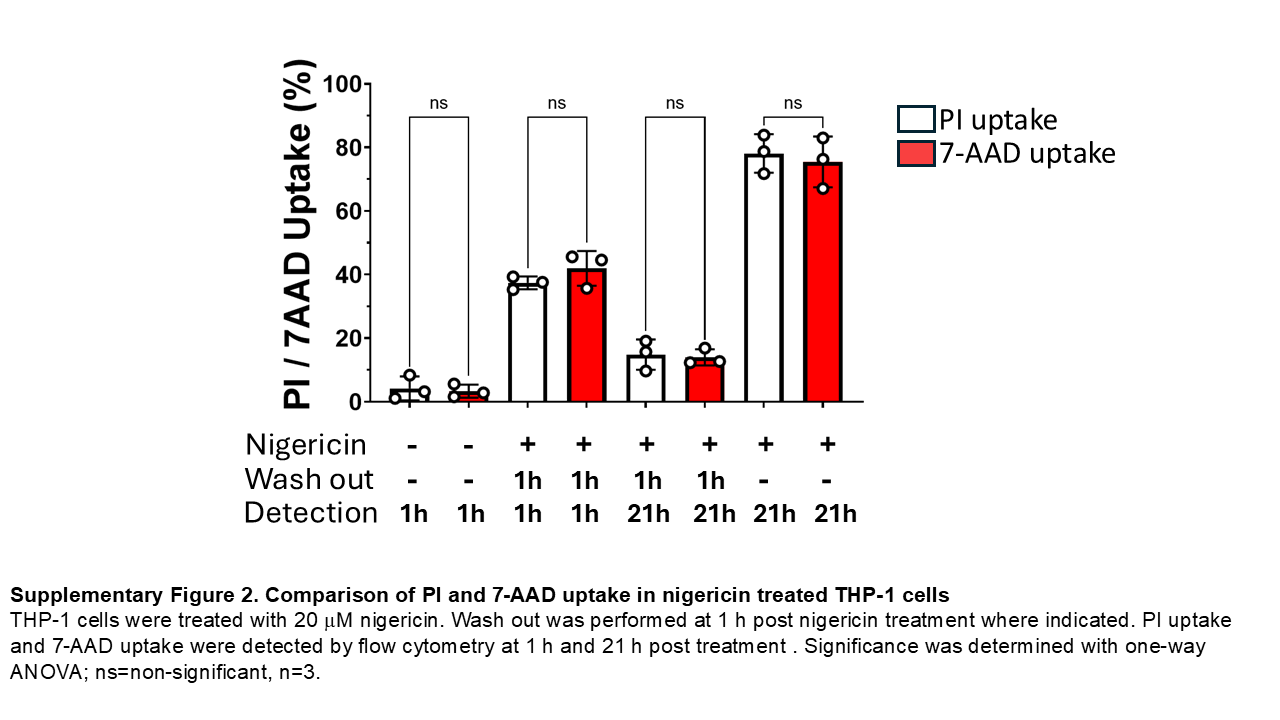

Supplement: Supplementary file 3 — Supplementary Figure 2 [file 41419_2025_7998_MOESM3_ESM.tif]

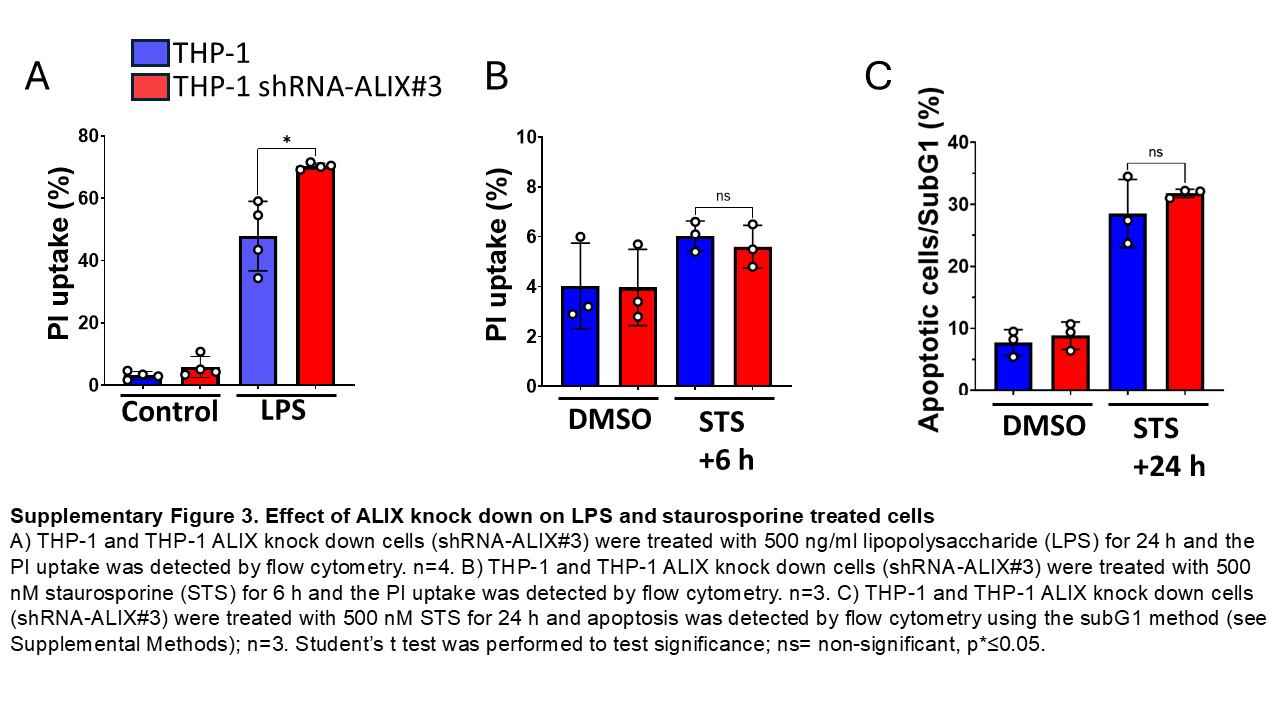

Supplement: Supplementary file 4 — Supplementary Figure 3 [file 41419_2025_7998_MOESM4_ESM.tif]

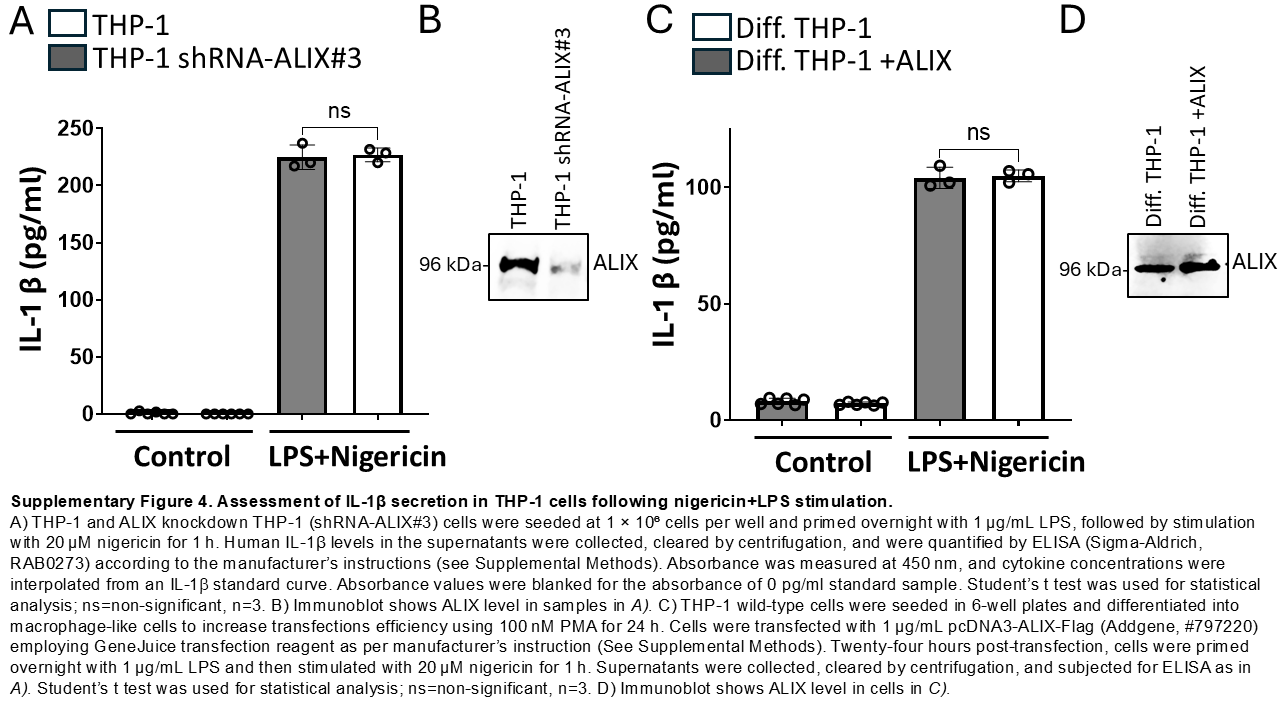

Supplement: Supplementary file 5 — Supplementary figure 4 [file 41419_2025_7998_MOESM5_ESM.tif]
