# Supplementary material for: ALIX mediates reversible gasdermin-D pore formation via the endosomal pathway to limit pyroptosis by active membrane repair
Source: Cell Death Dis. 2025 Oct 6;16(1):681. doi: 10.1038/s41419-025-07998-y (PMC12501301; doi:10.1038/s41419-025-07998-y)

Figure 1A

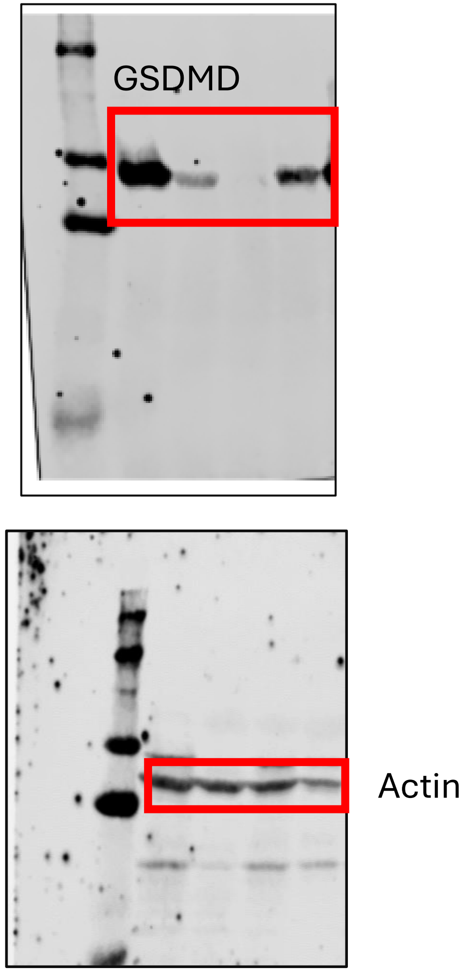

Figure 1C

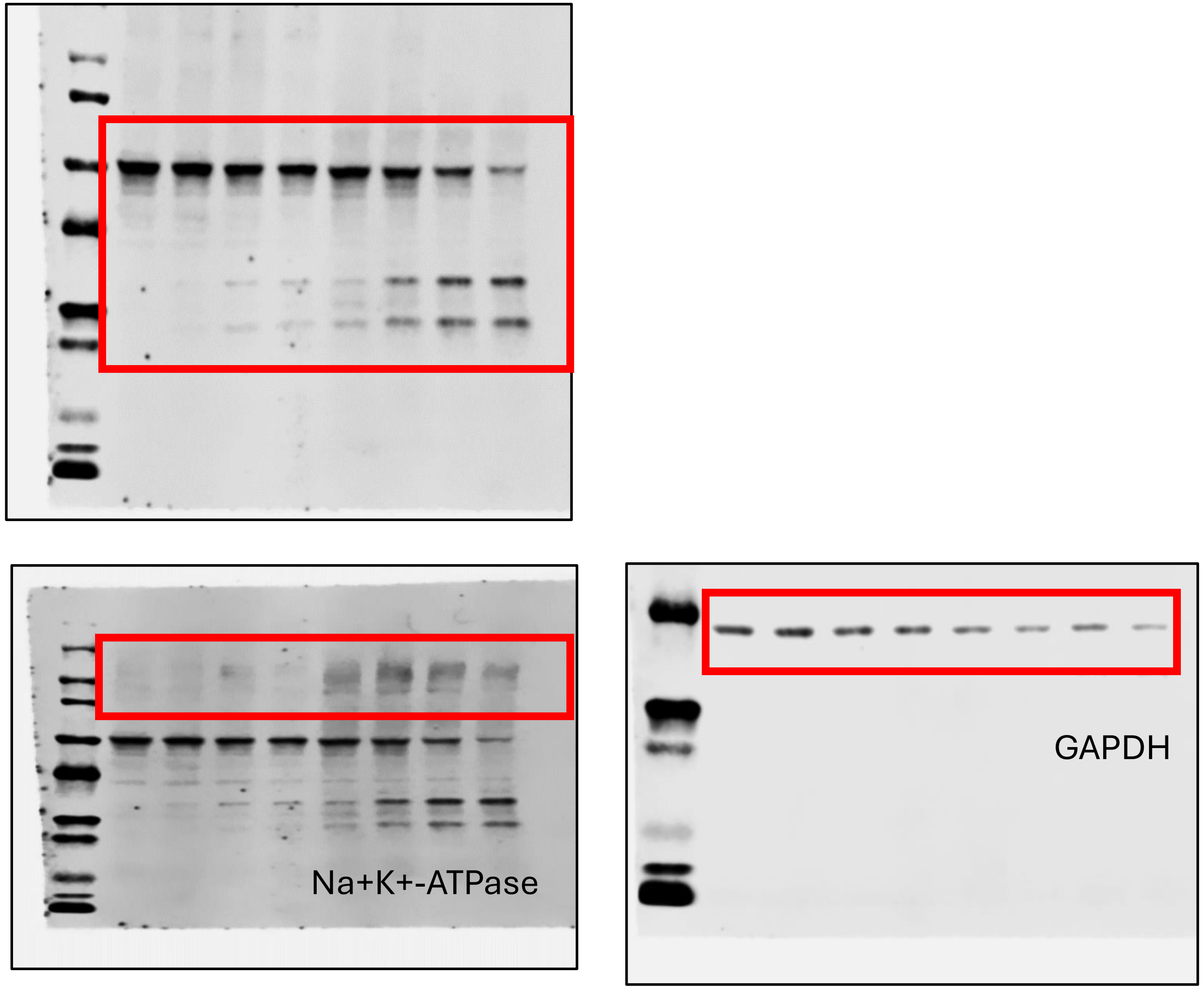

Figure 2C

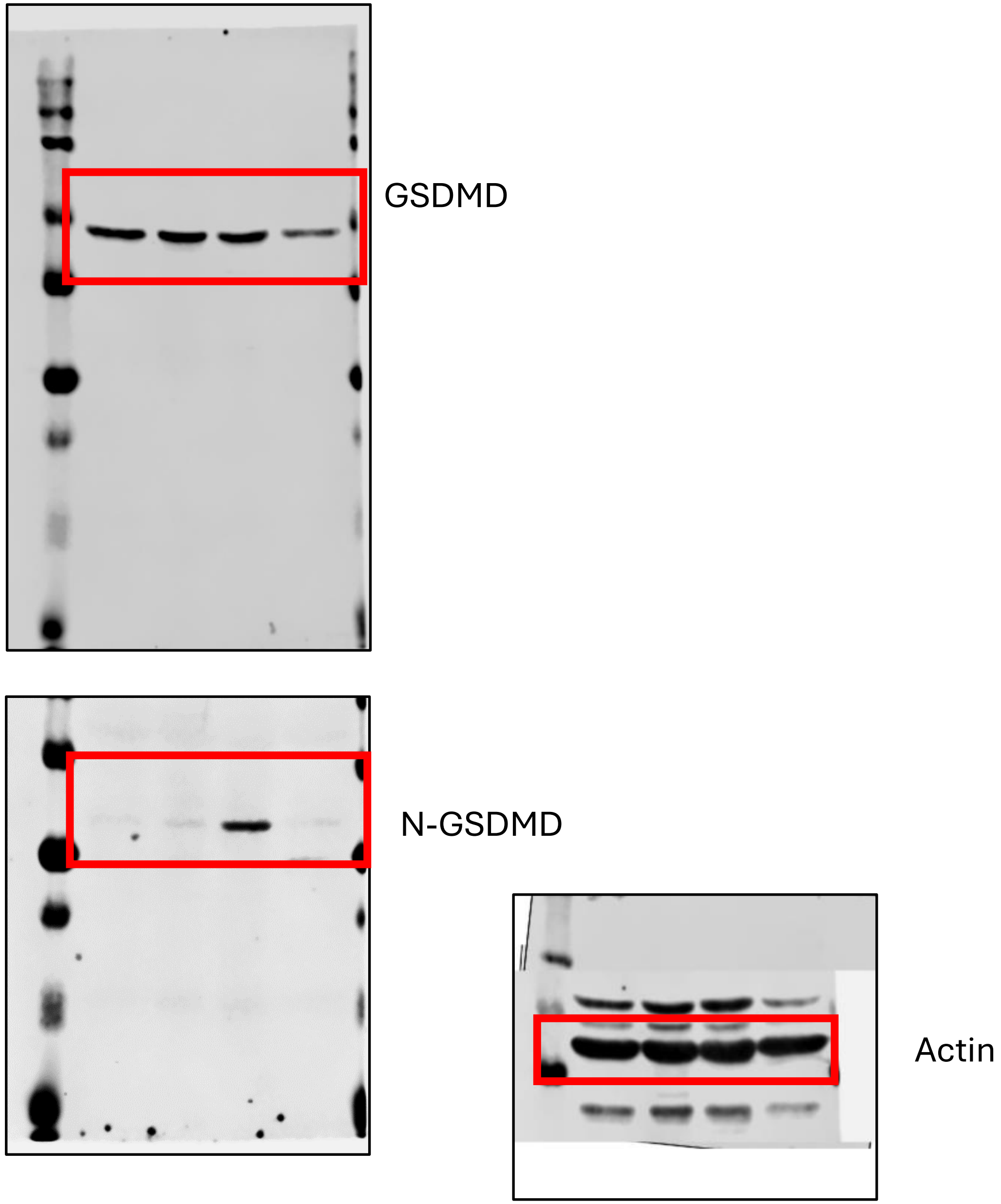

Figure 2D

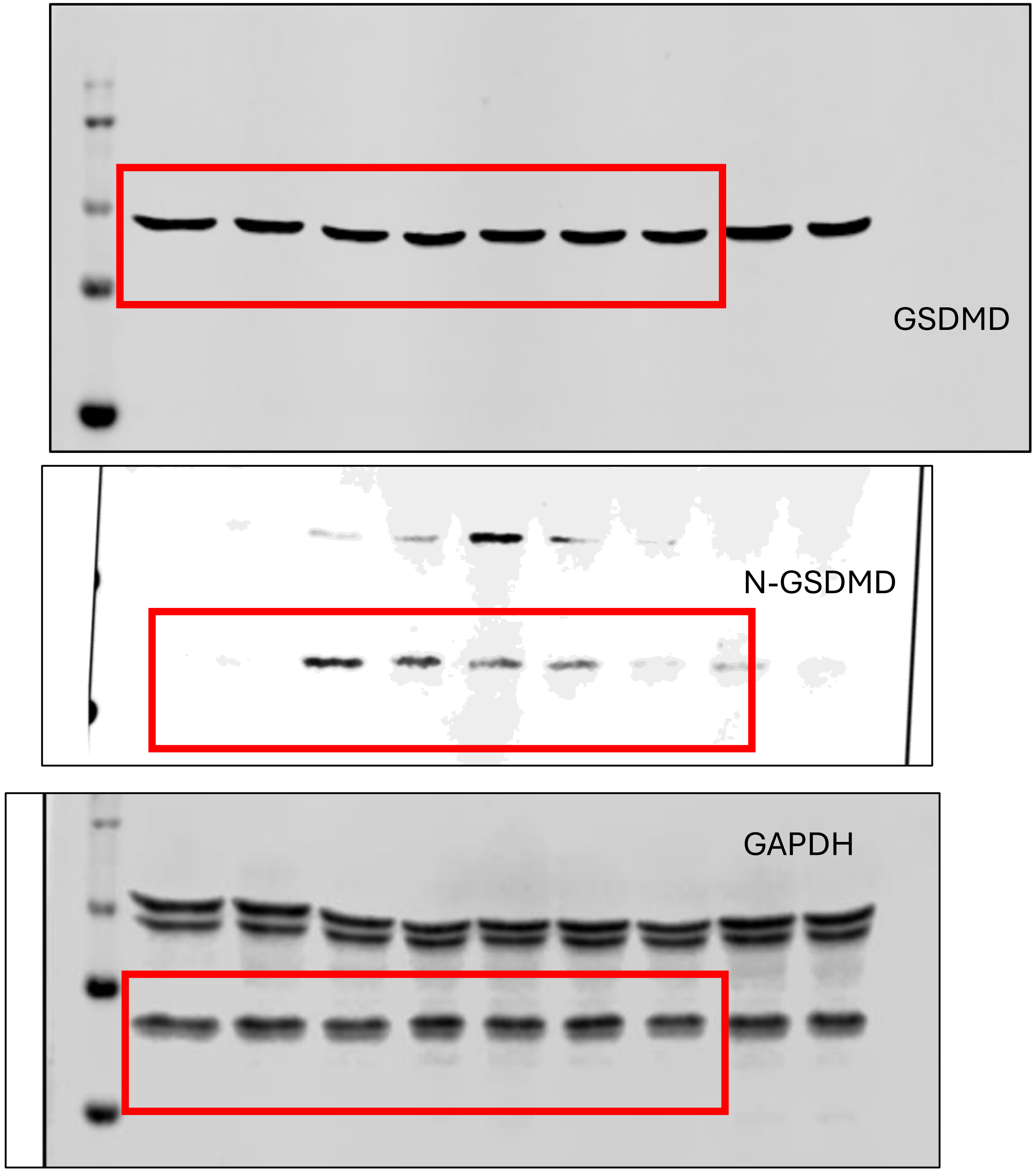

Figure 3C

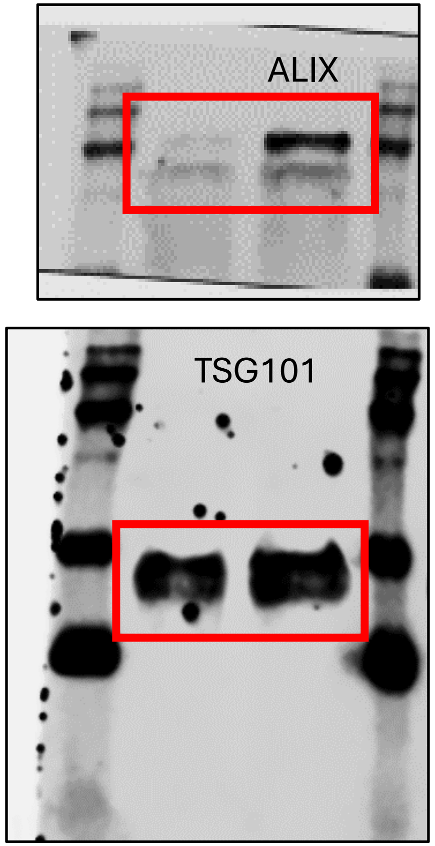

Figure 3D

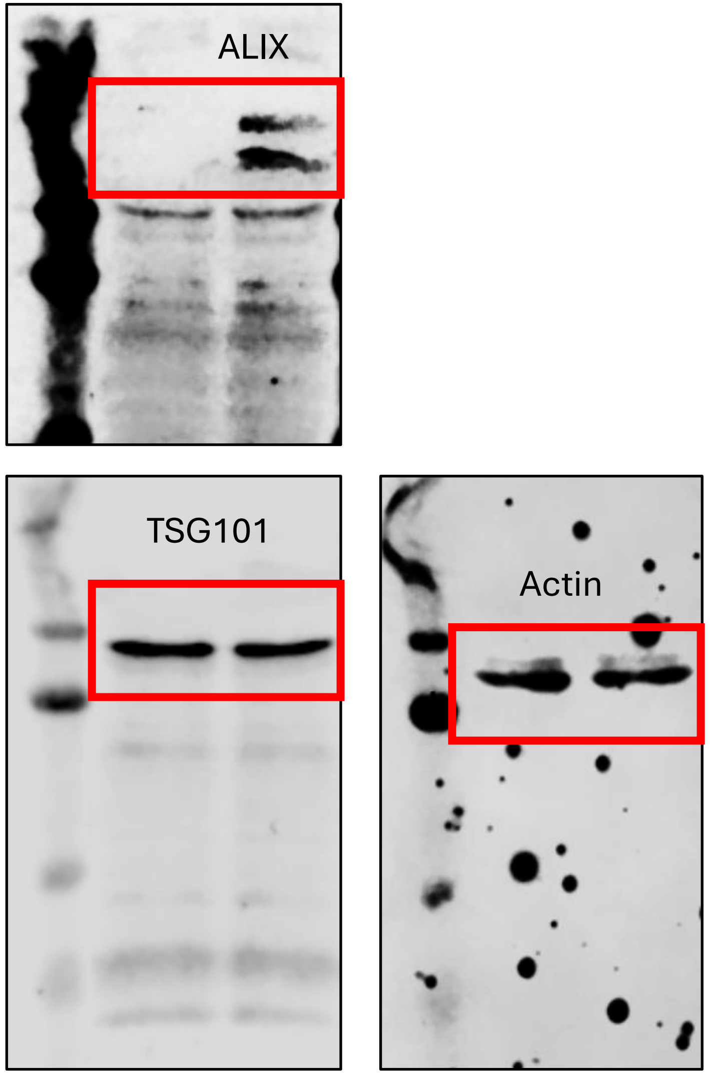

Figure 4A

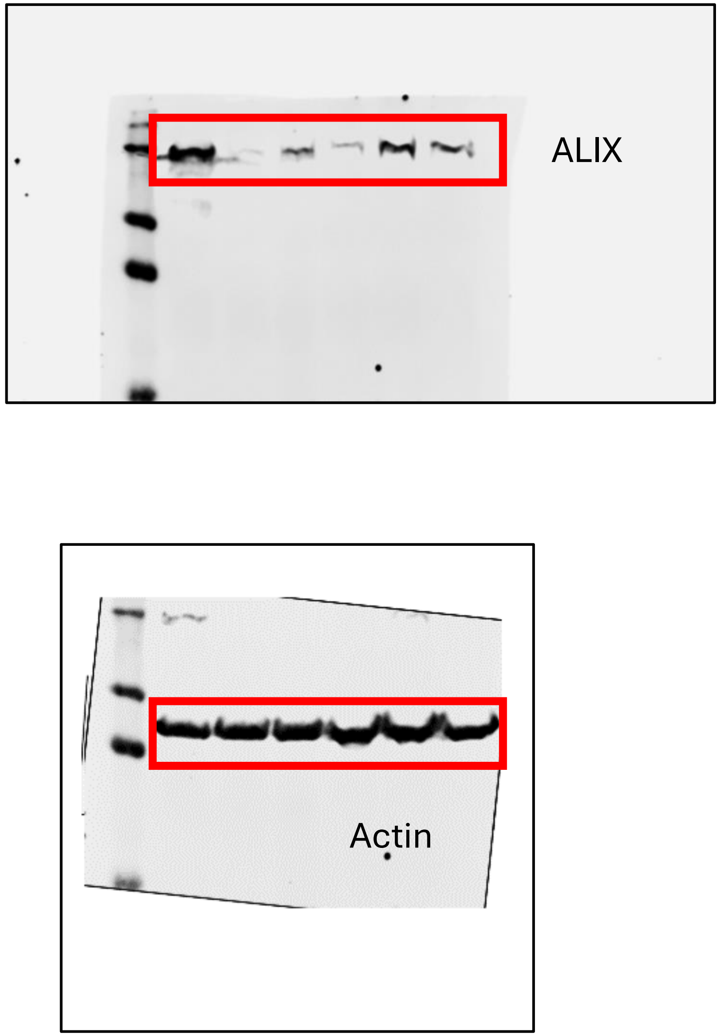

Figure 4C

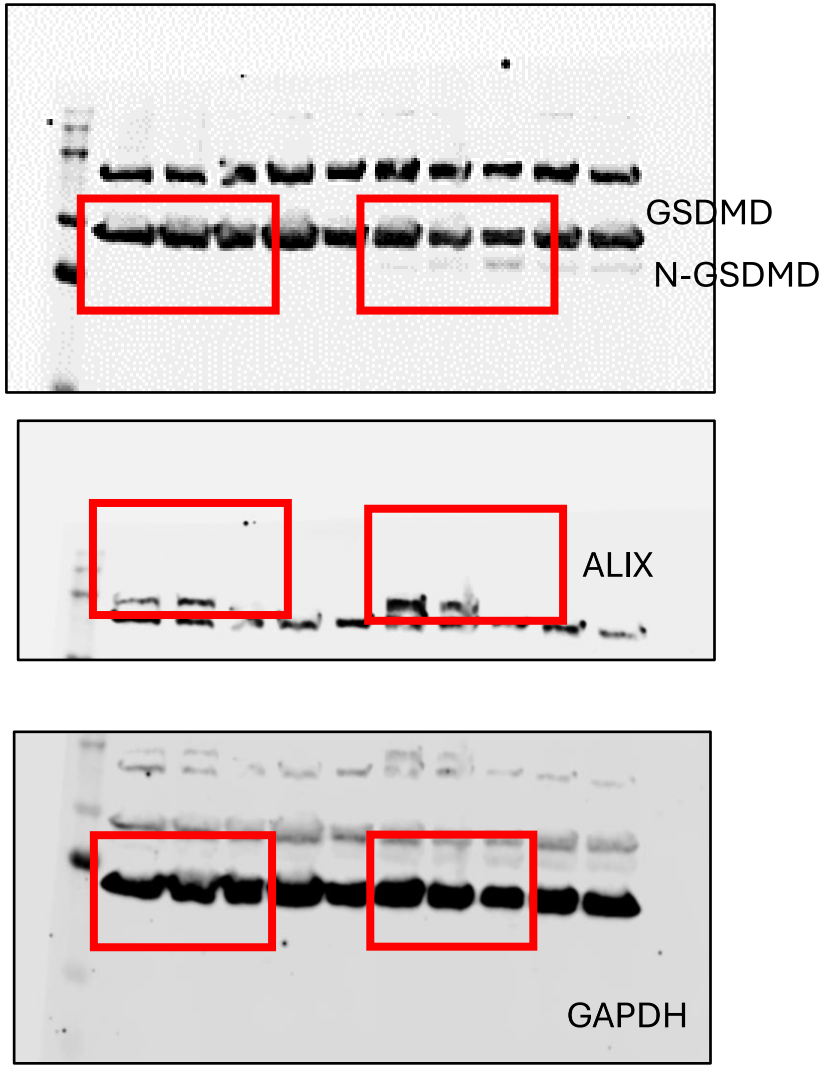

Figure 4D

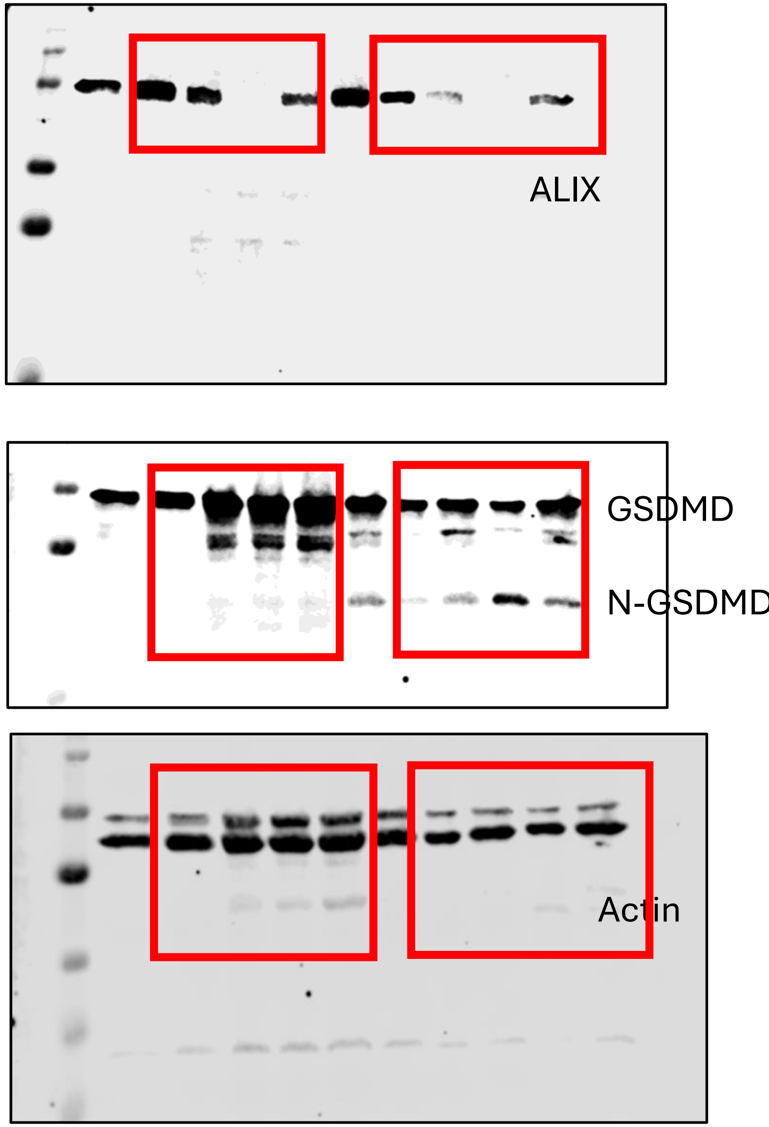

Figure 5A

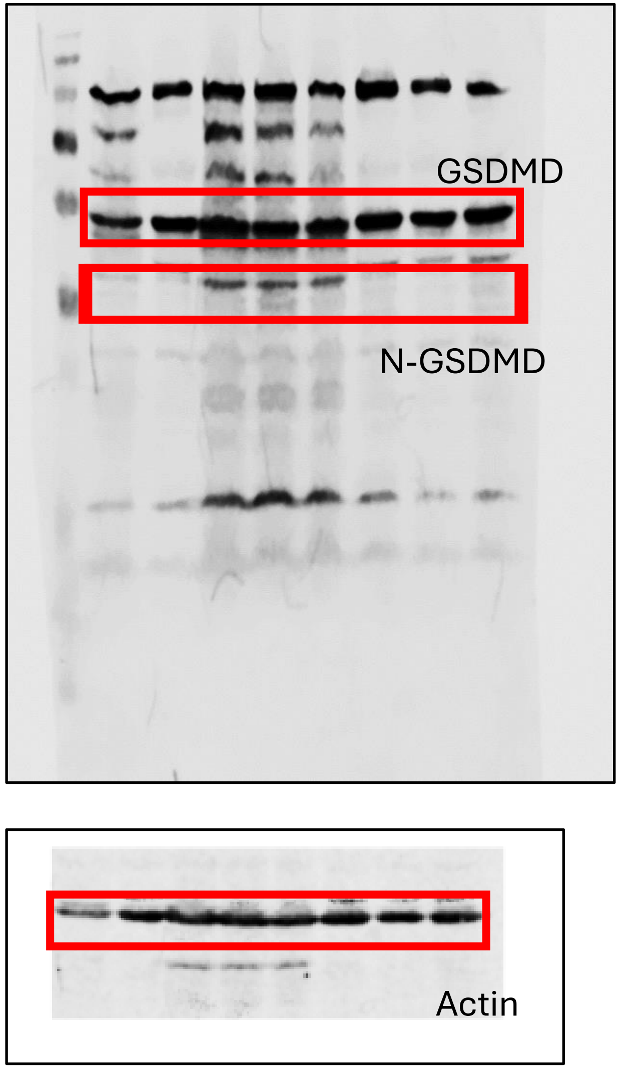

Figure 5B

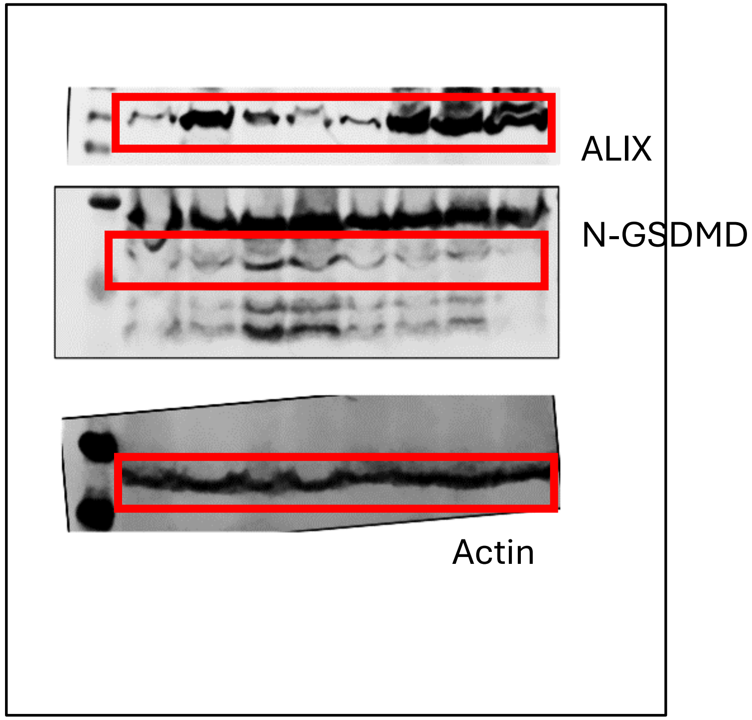

Figure 6A

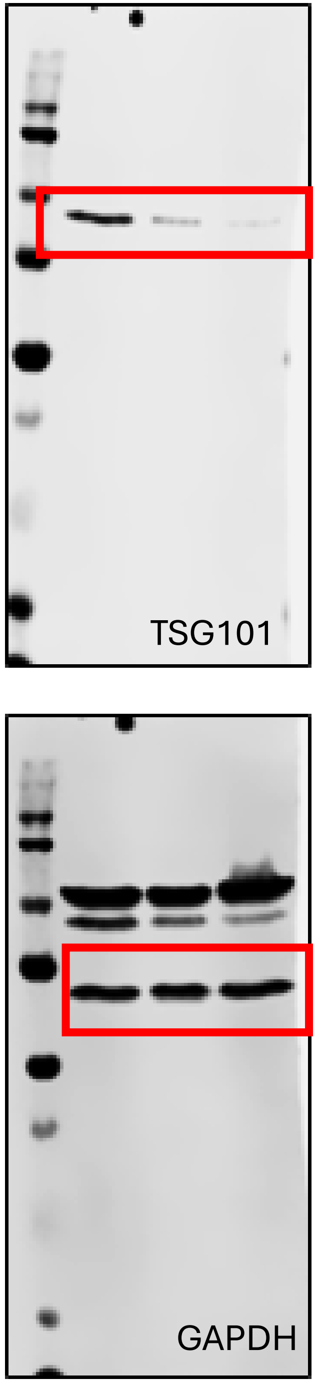

Figure 6D

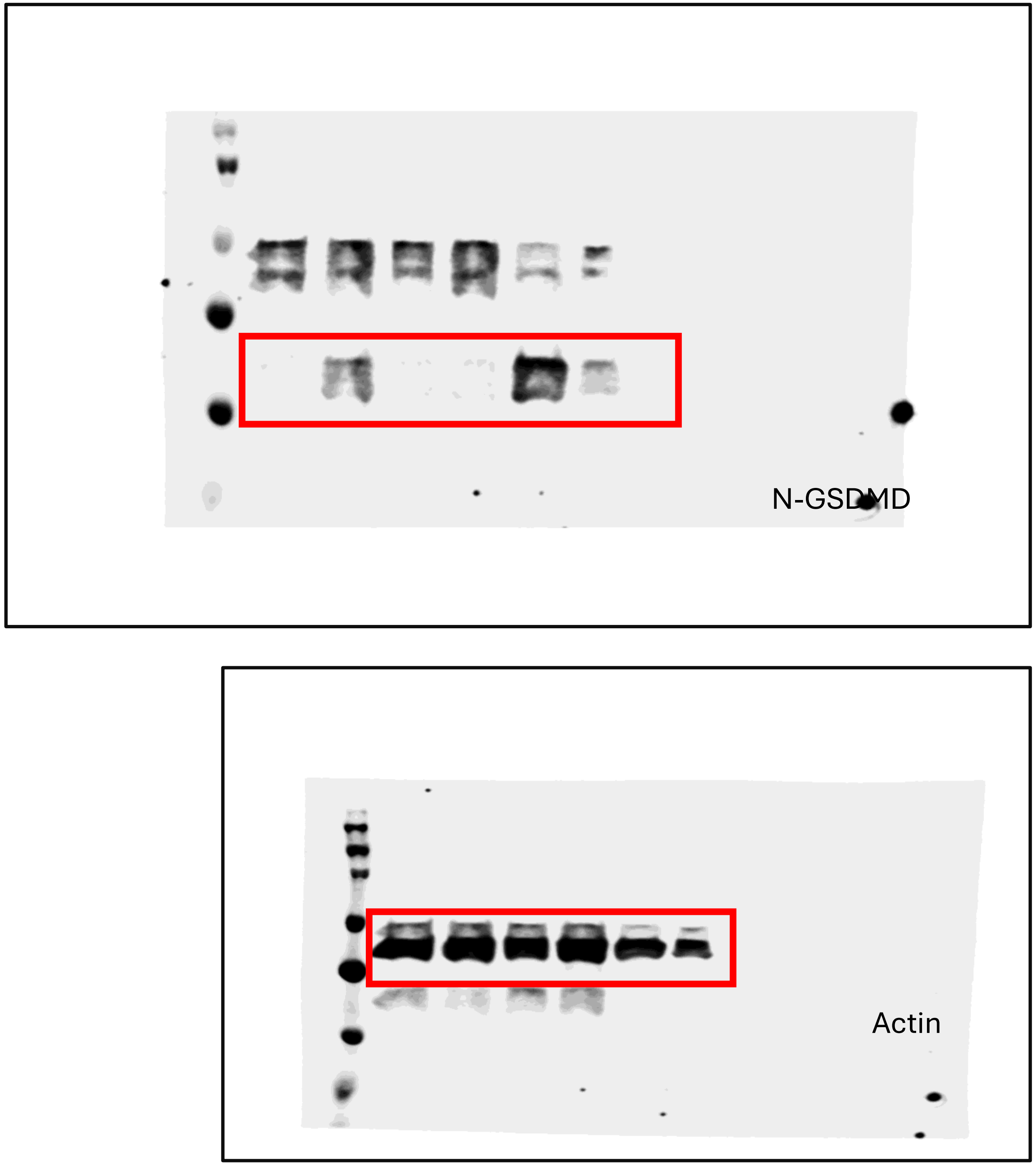

Supplement: Supplementary file 6 — Full Western Blots [file 41419_2025_7998_MOESM6_ESM.pdf]
