# Supplementary material for: ALIX mediates reversible gasdermin-D pore formation via the endosomal pathway to limit pyroptosis by active membrane repair
Source: Cell Death Dis. 2025 Oct 6;16(1):681. doi: 10.1038/s41419-025-07998-y (PMC12501301; doi:10.1038/s41419-025-07998-y)

Flow Cytometry Data Main Figures

Figure 1B Experiment 1

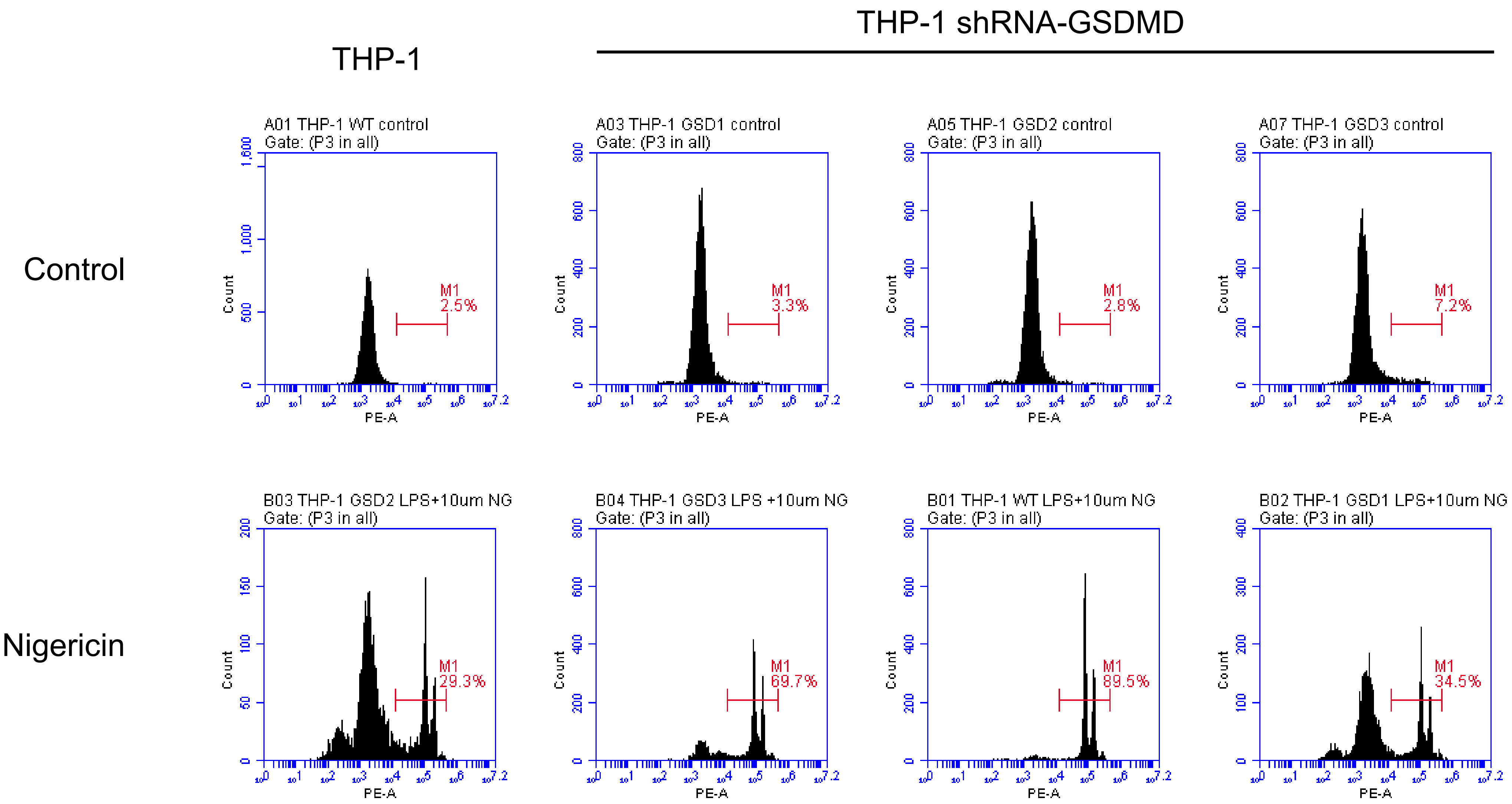

Figure 1B Experiment 2

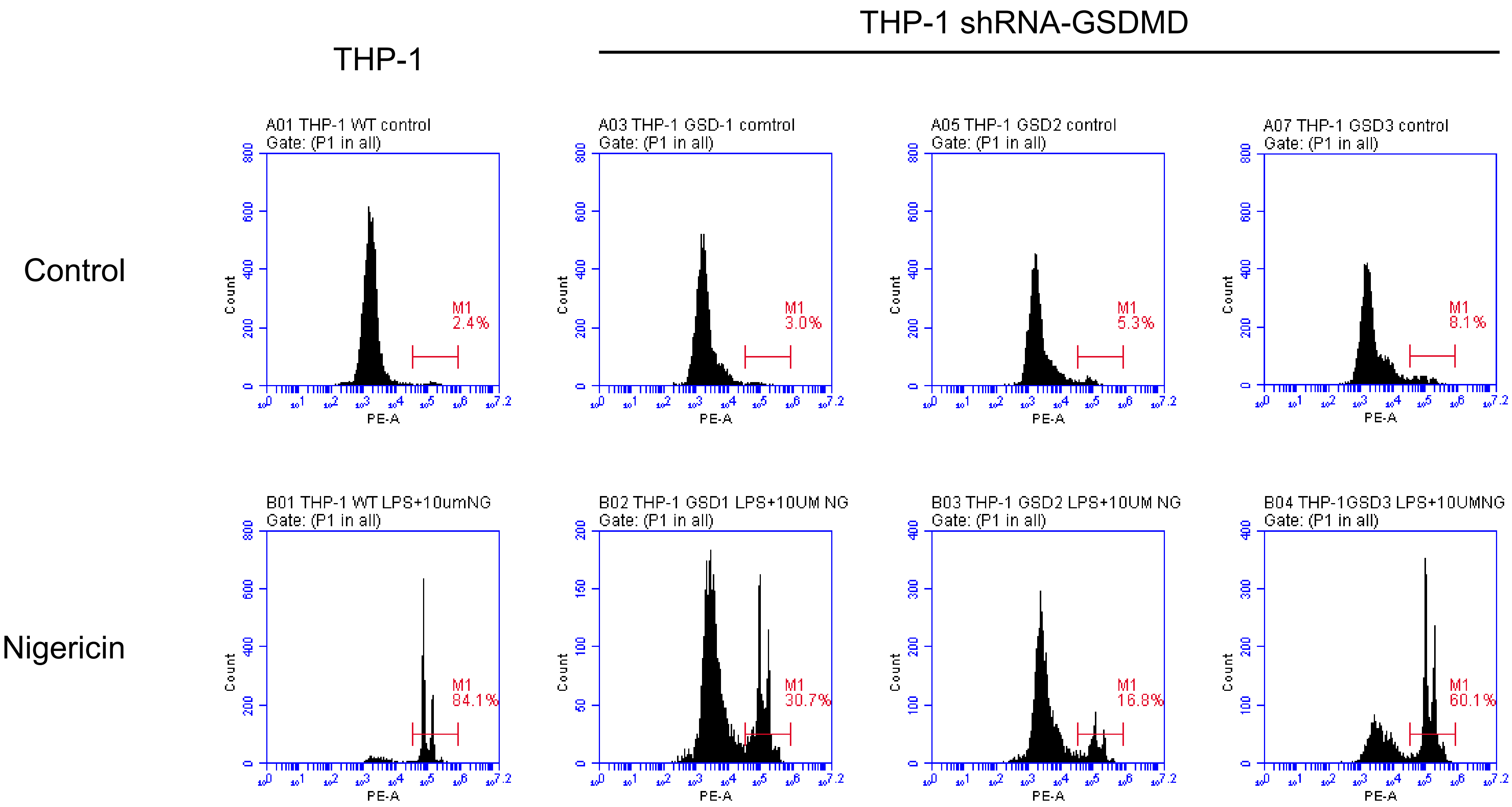

Figure 1B Experiment 3

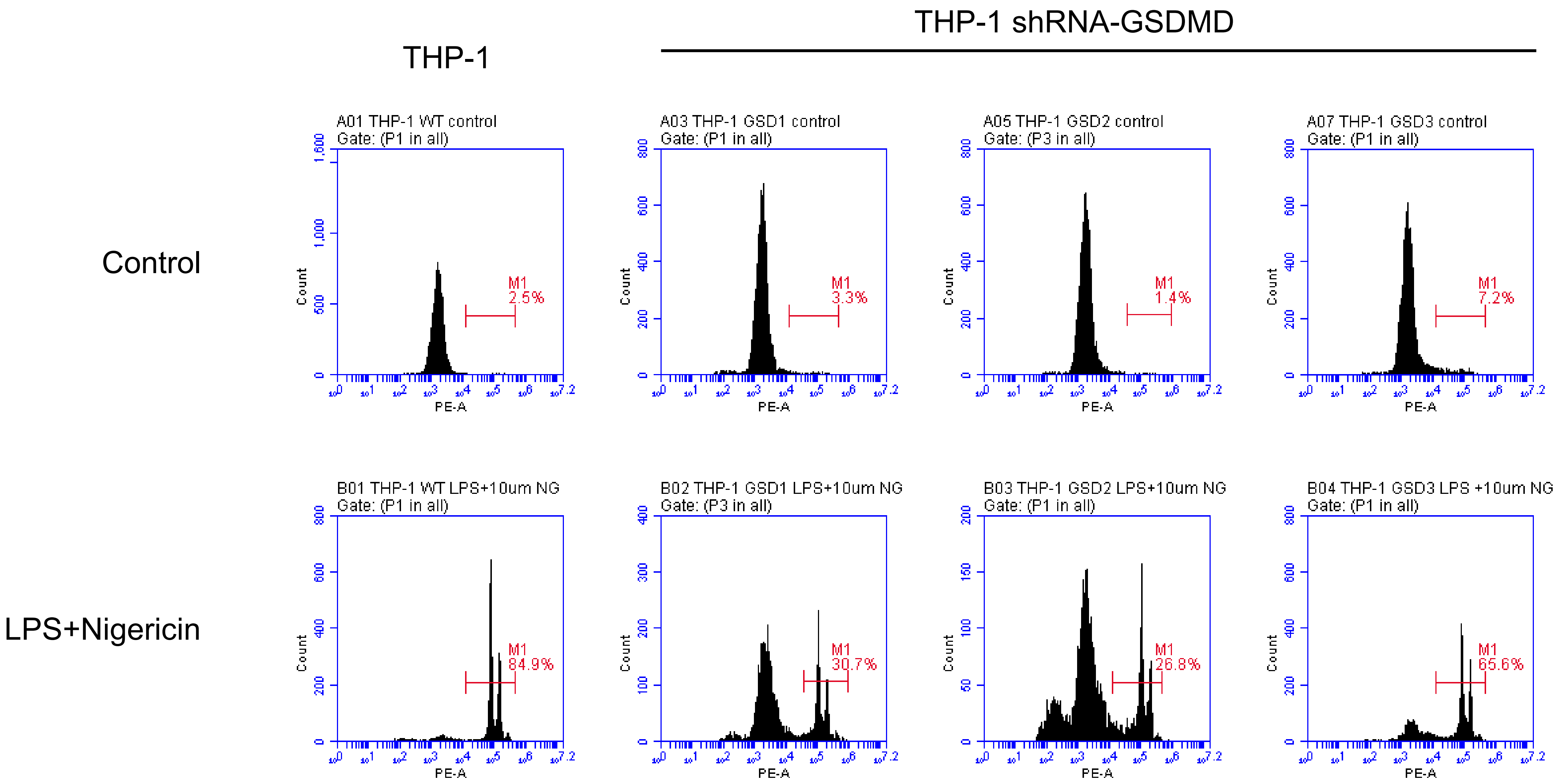

Figure 2B Experiment 1

Control

Nigericin/
Detect at 1h

Nigericin/
Wash out at
1h Detect at
21h

Nigericin/No
wash/Detect
at 21h

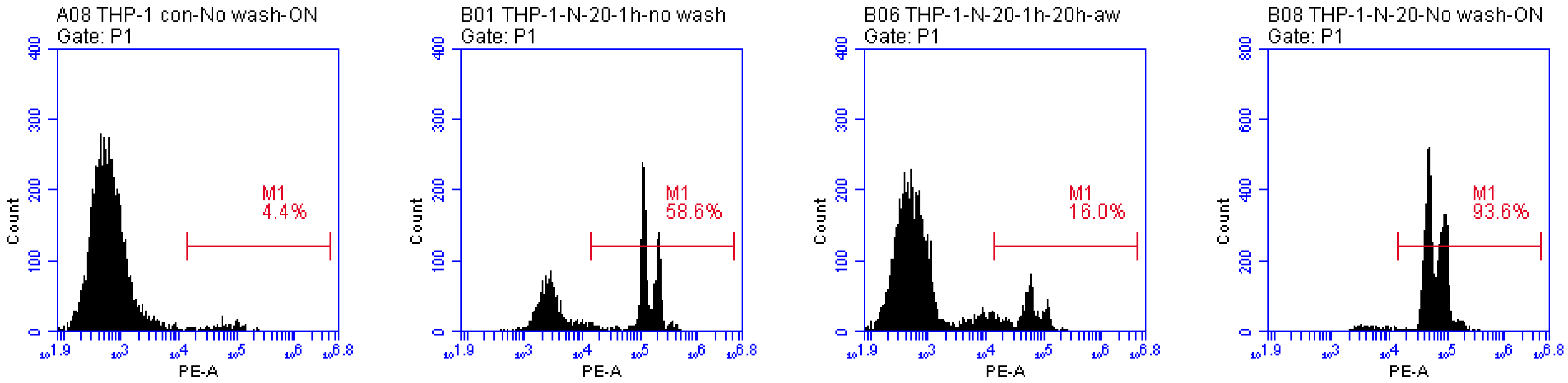

Figure 2B Experiment 2

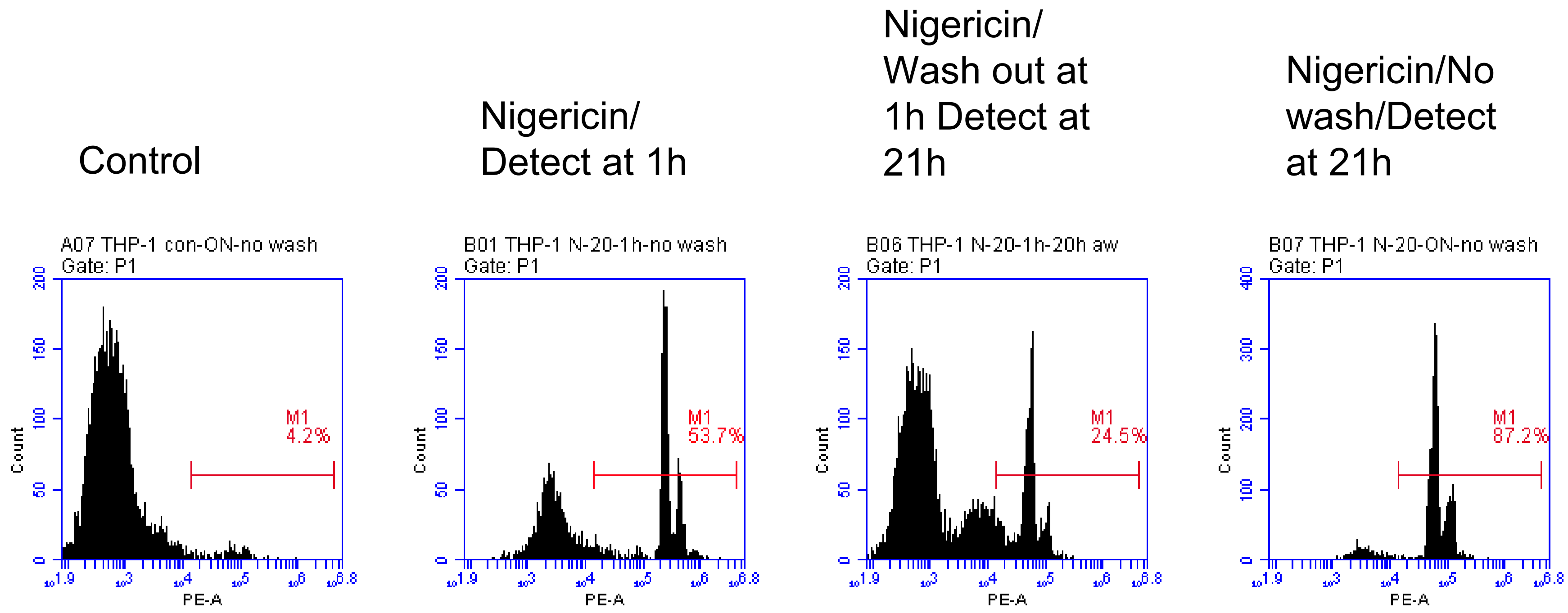

Figure 2B Experiment 3

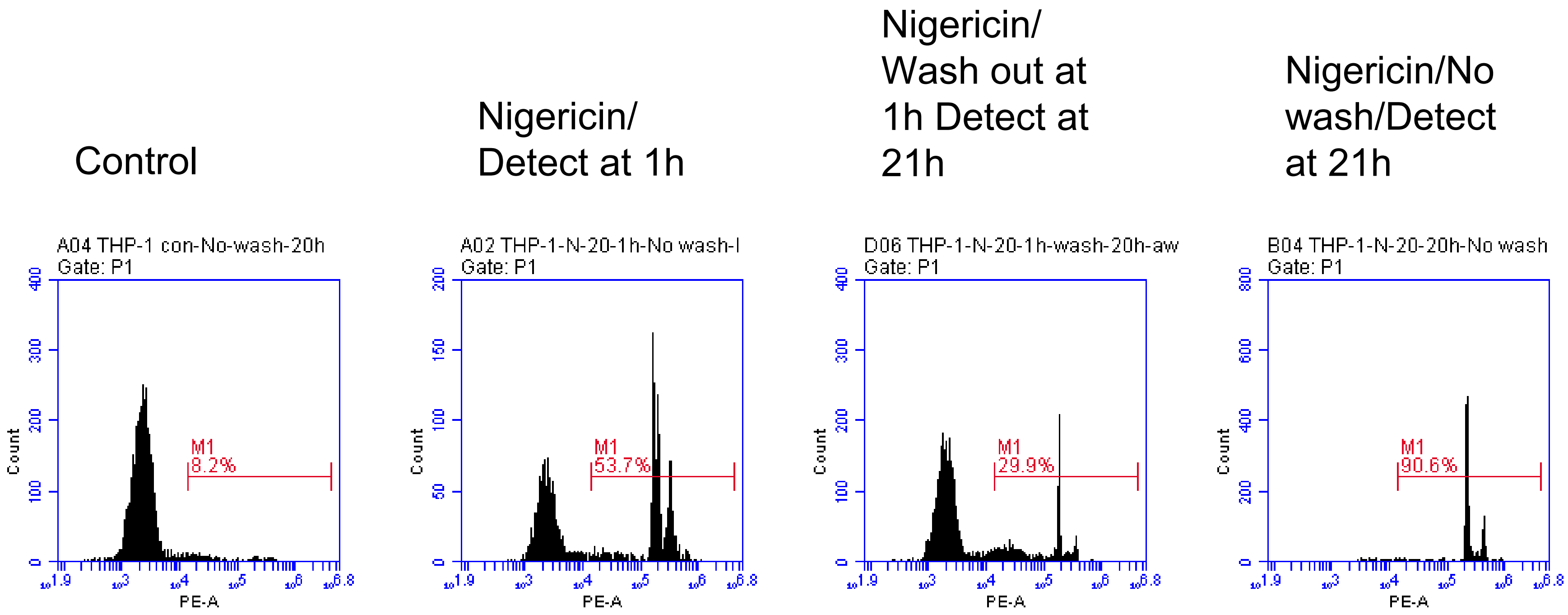

Figure 2B Experiment 4

Control

Nigericin/
Detect at 1h

Nigericin/
Wash out at
1h Detect at
21h

Nigericin/No
wash/Detect
at 21h

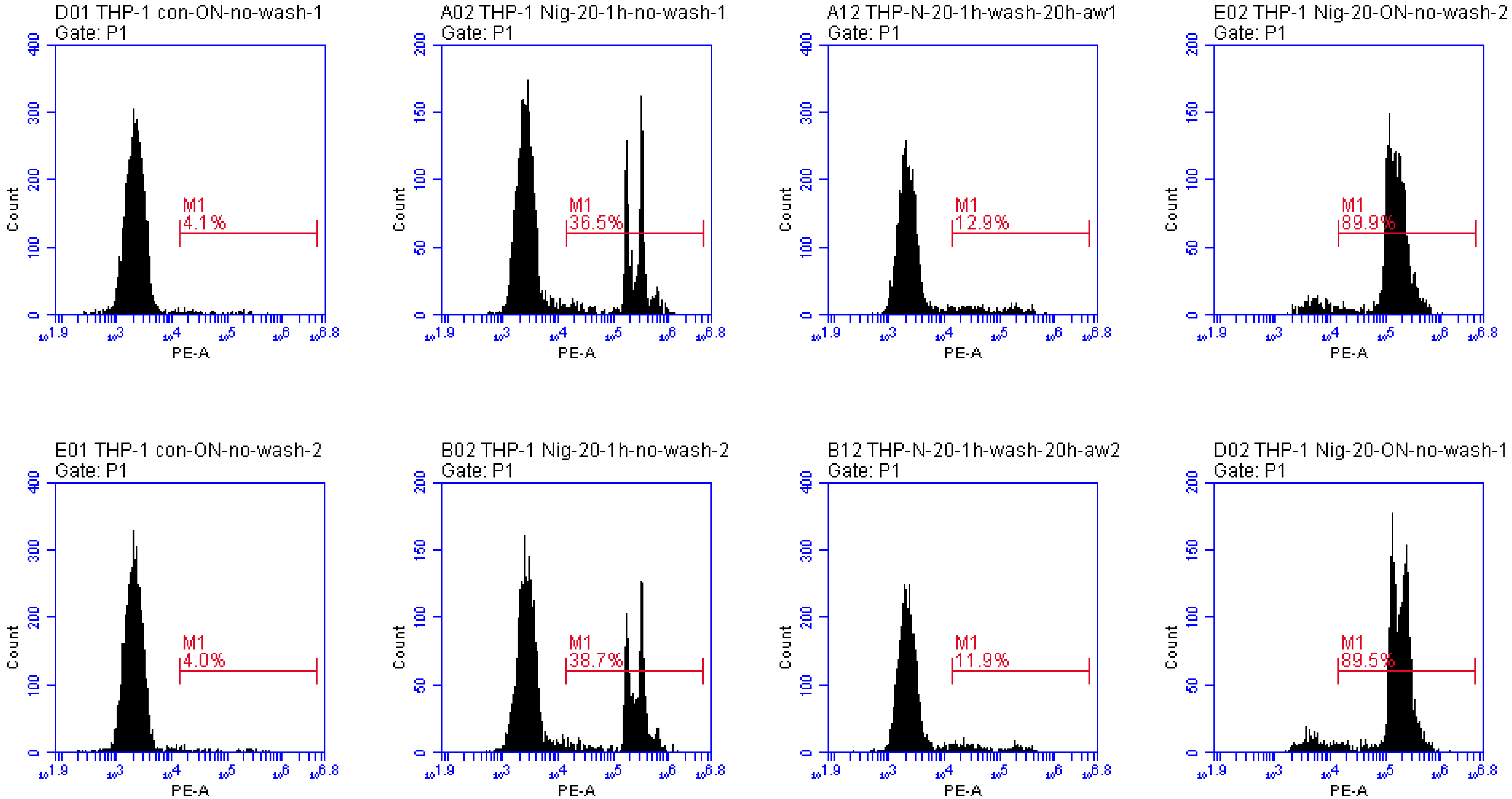

Figure 2F Experiment 1

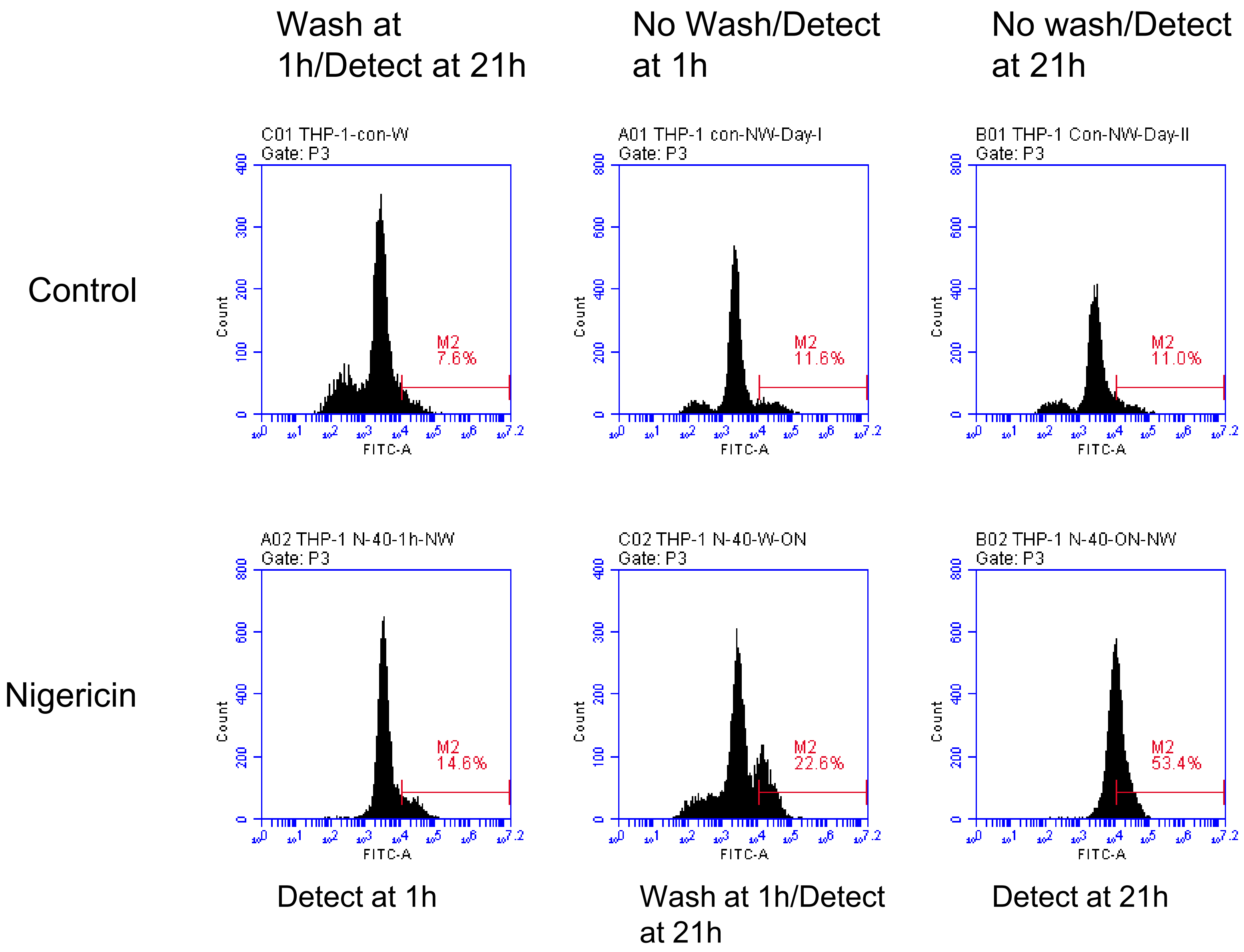

Figure 2F Experiment 2

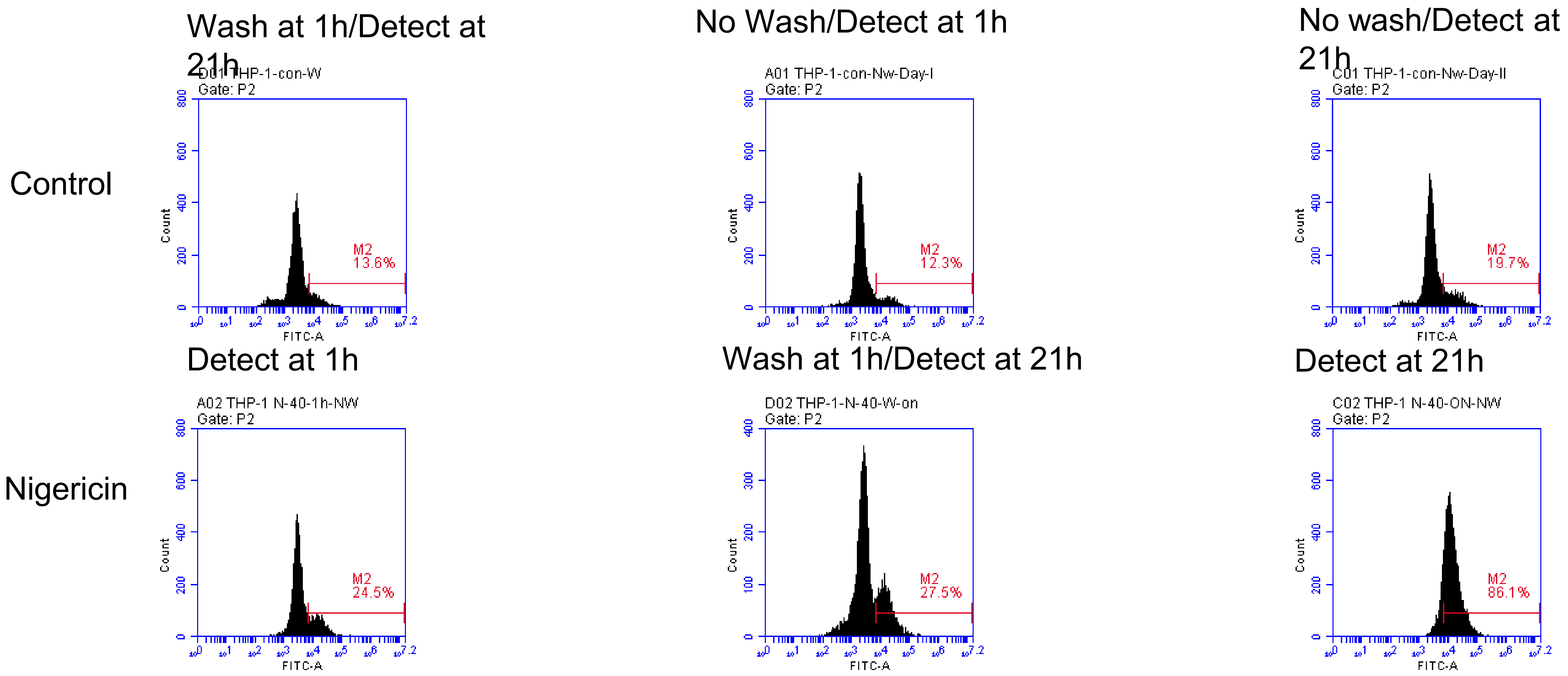

Figure 2F Experiment 3

Wash at 1h/Detect at 21h

No Wash/Detect at 1h

No wash/Detect at 2[illegible]

Control

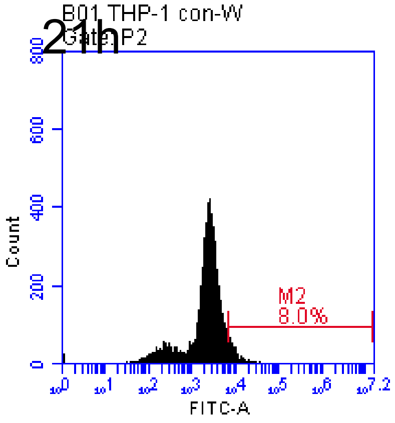

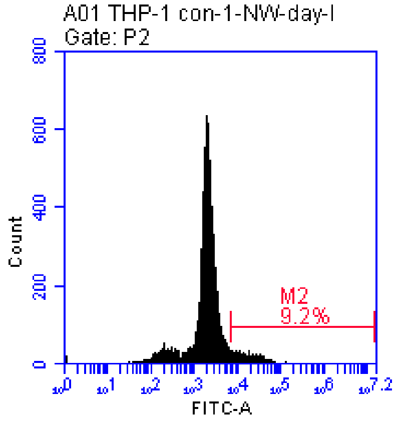

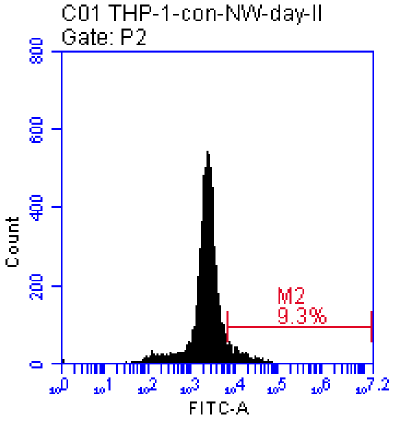

Detect at 1h

Wash at 1h/Detect at 21h

Detect at 21h

Nigericin

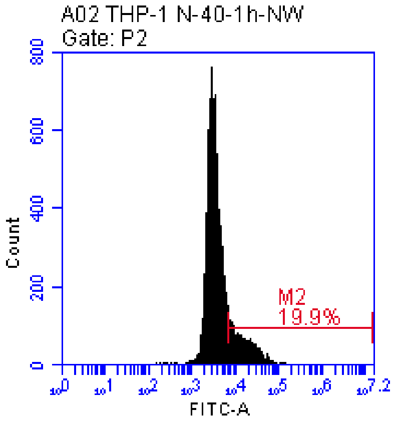

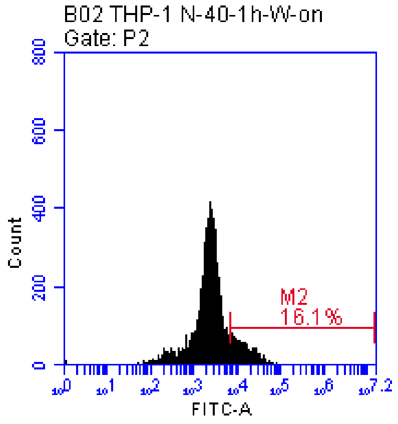

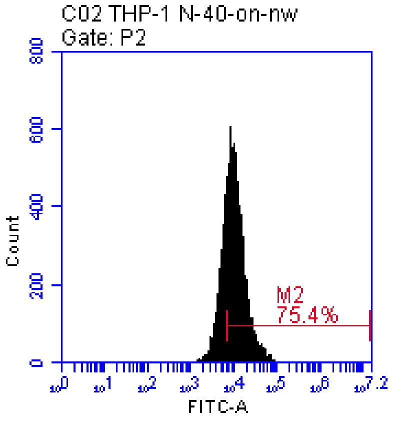

Figure 4B Experiment 1

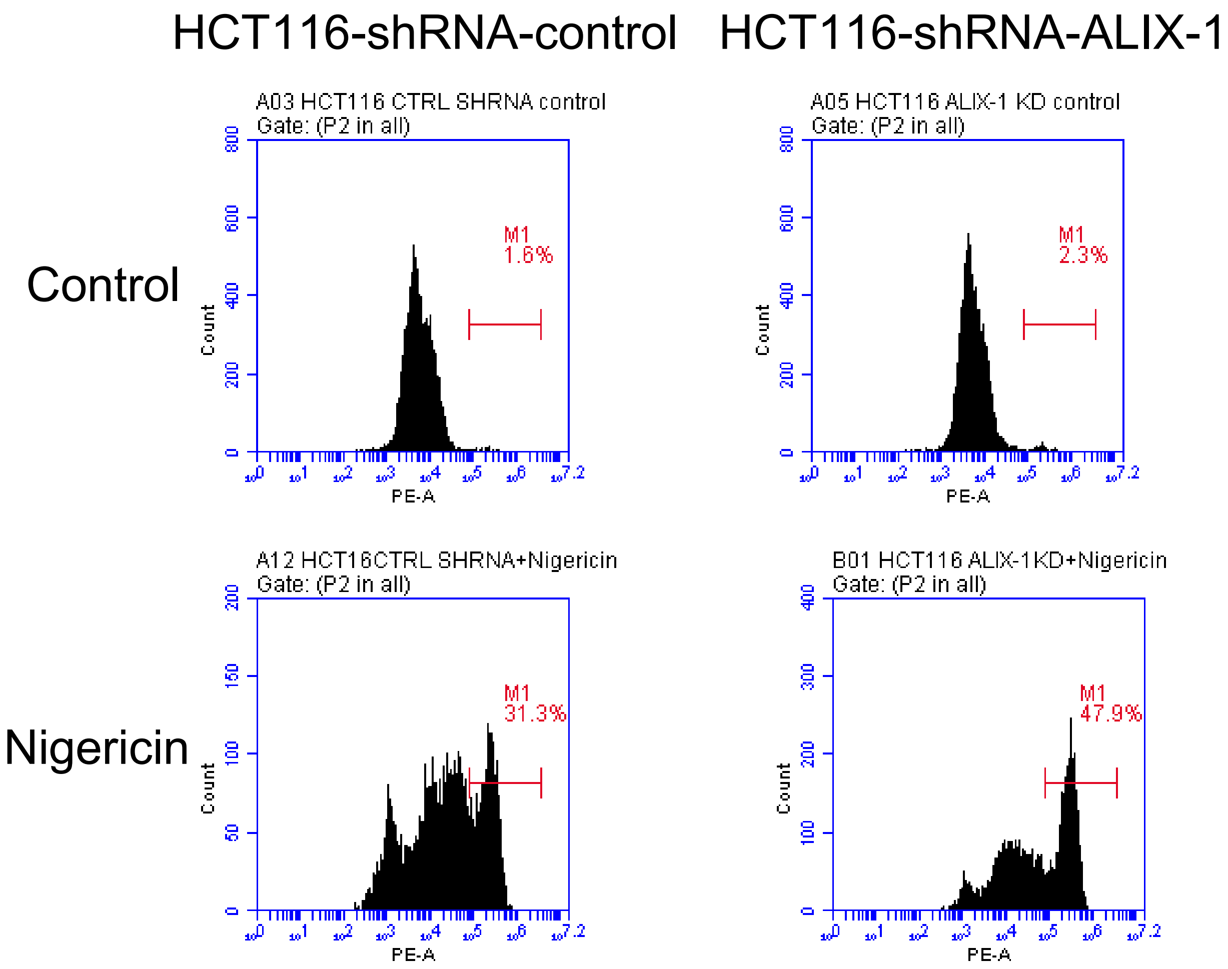

Figure 4B Experiment 2

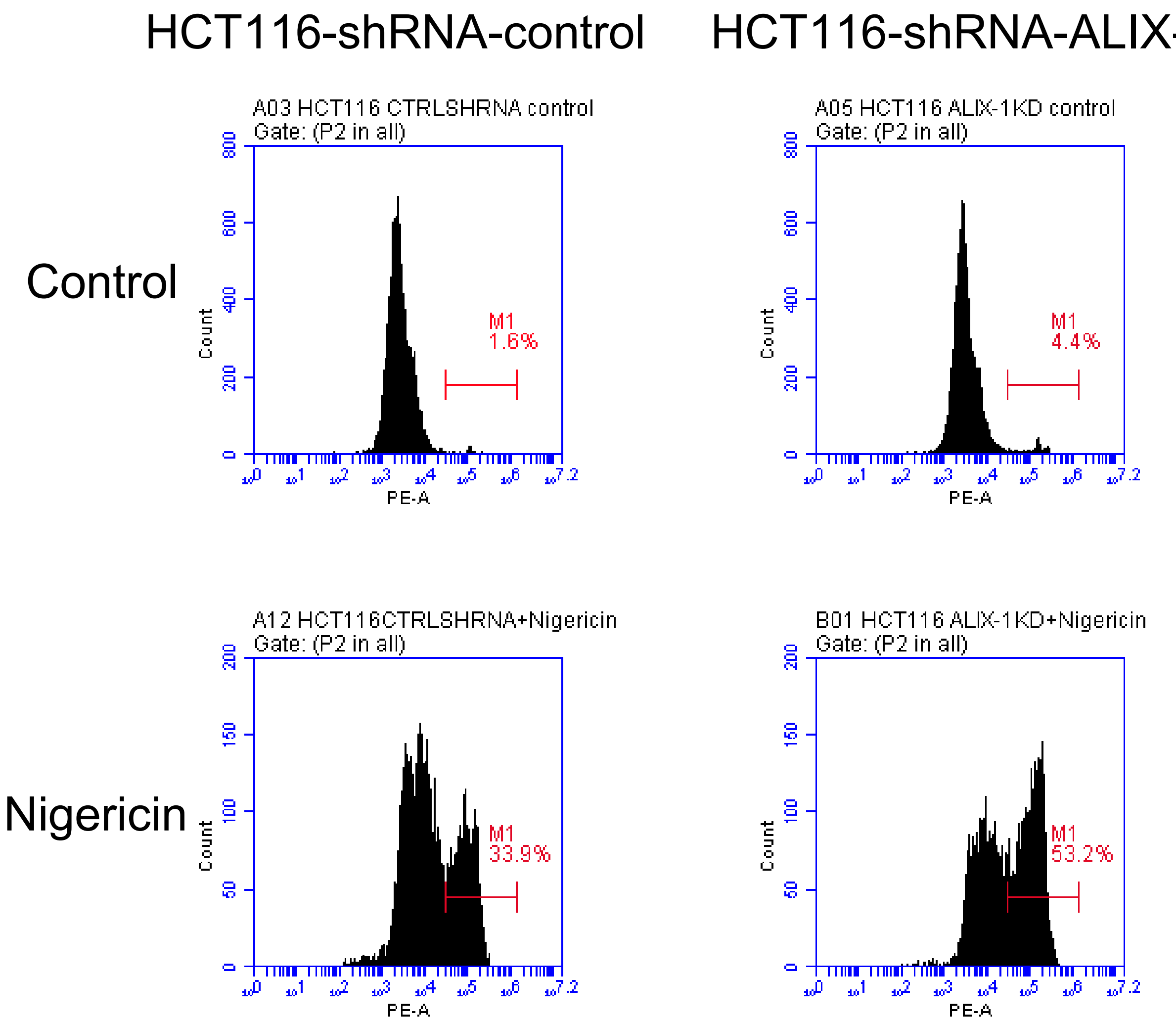

Figure 4B Experiment 3

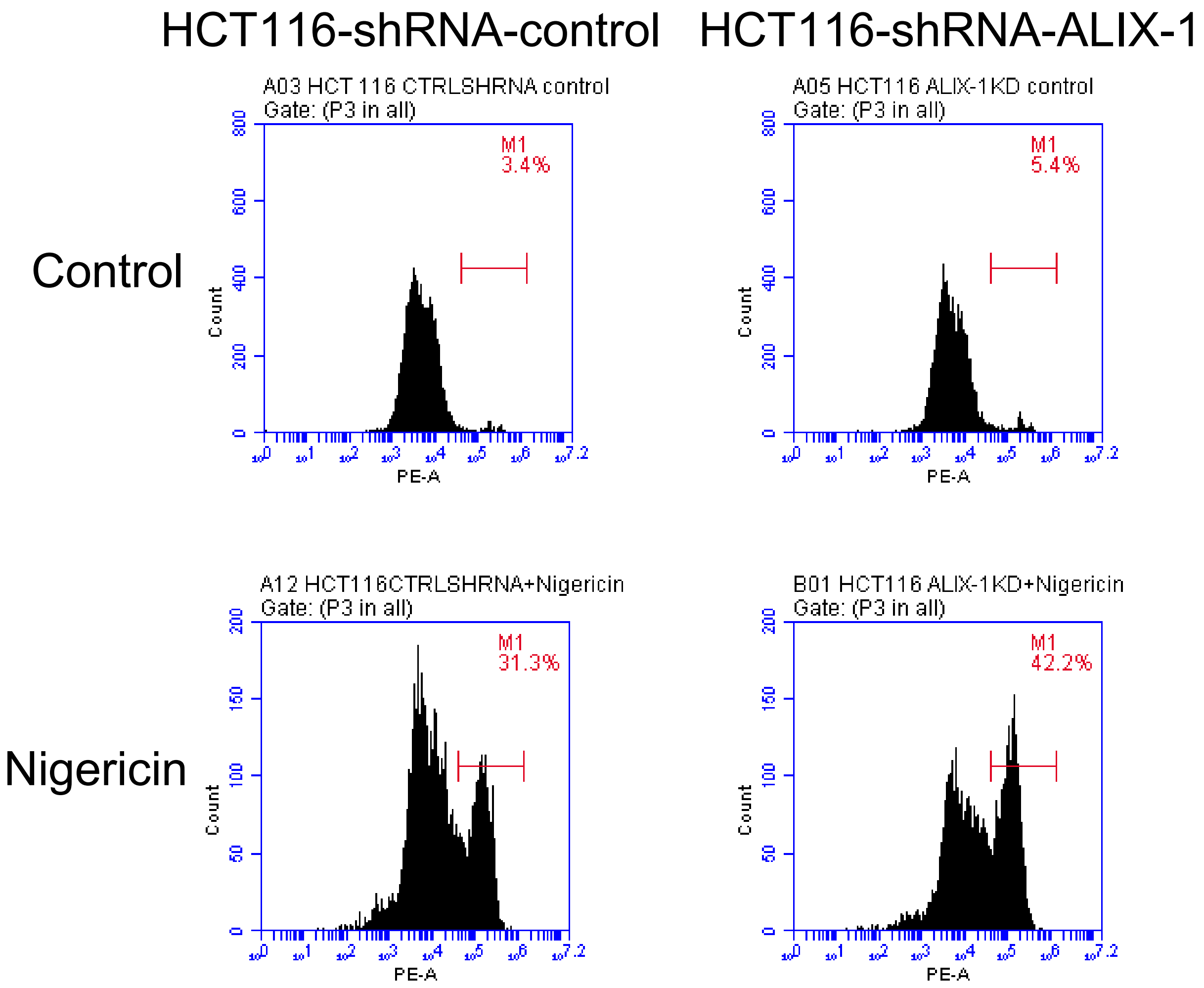

Figure 4E Experiment 1

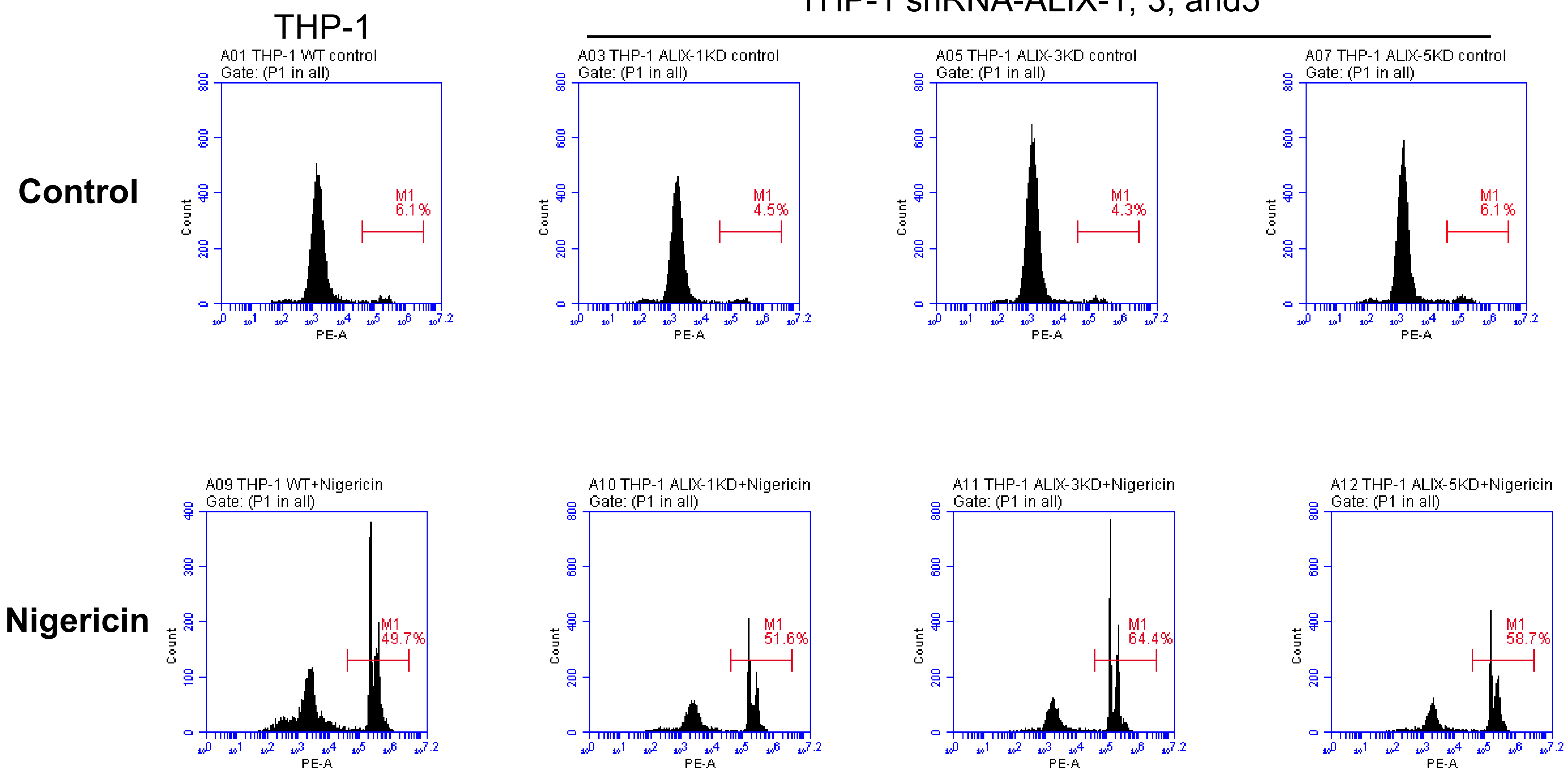

Figure 4E Experiment 2

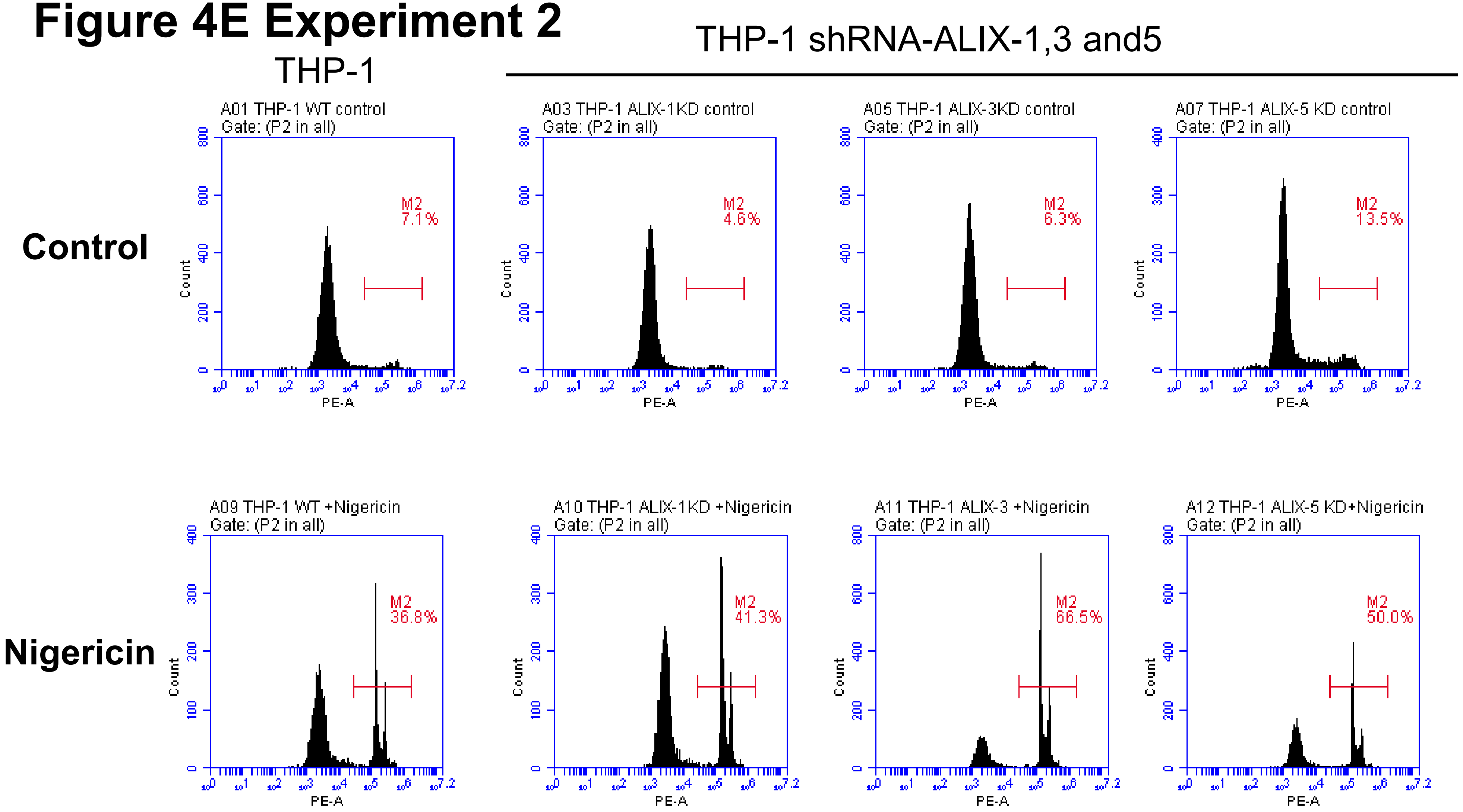

Figure 4E Experiment 3

THP-1

THP-1 shRNA control

THP-1 shRNA-ALIX-1,3 and5

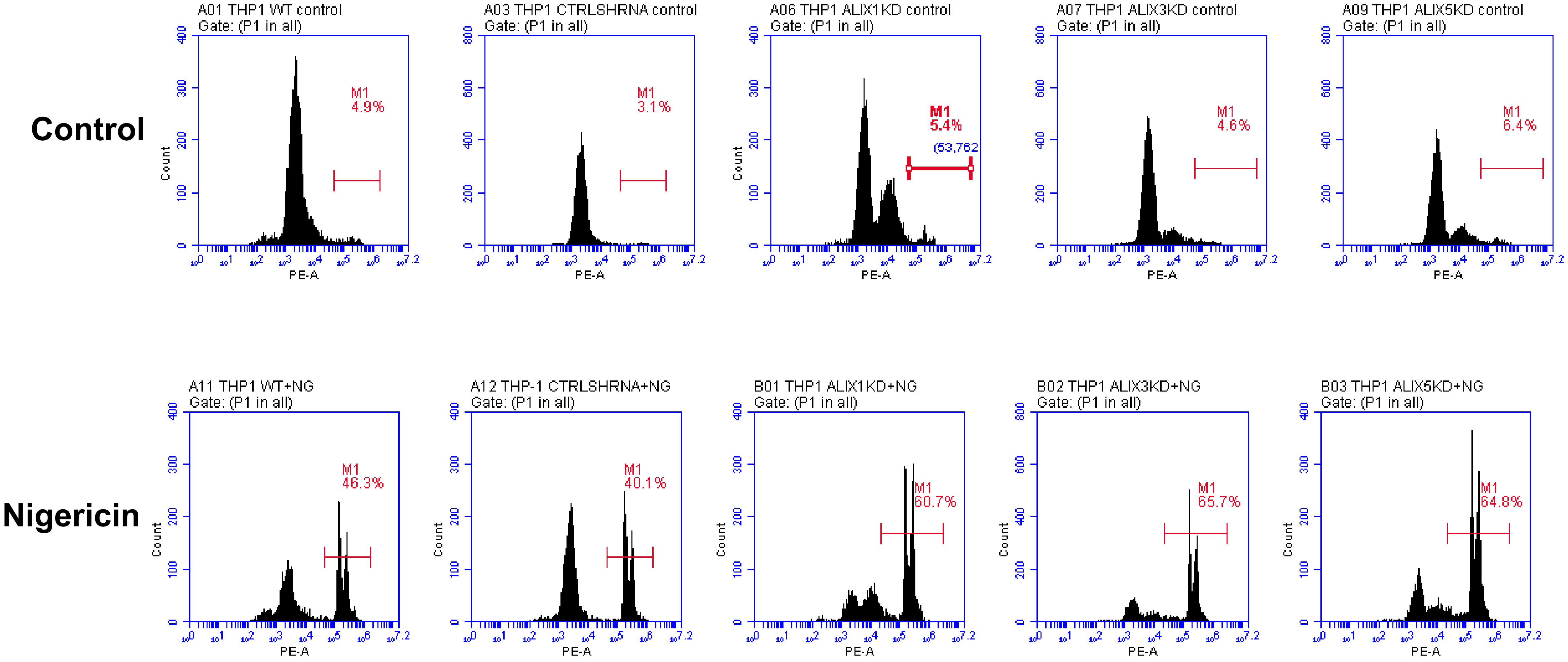

Figure 5C

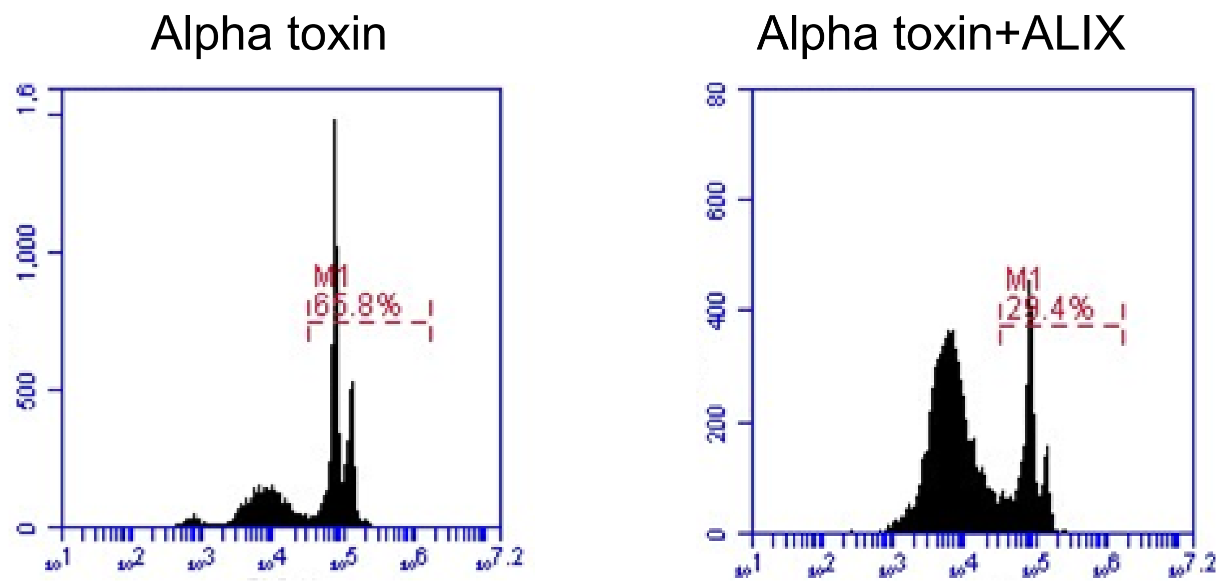

Figure 6B Experiment 1

Control | Nigericin 1h | Nigericin Wash Out 1h/ Detect 21h | Nigericin No Wash/Detect 21h

THP-1

THP-1 shRNA-Tsg101

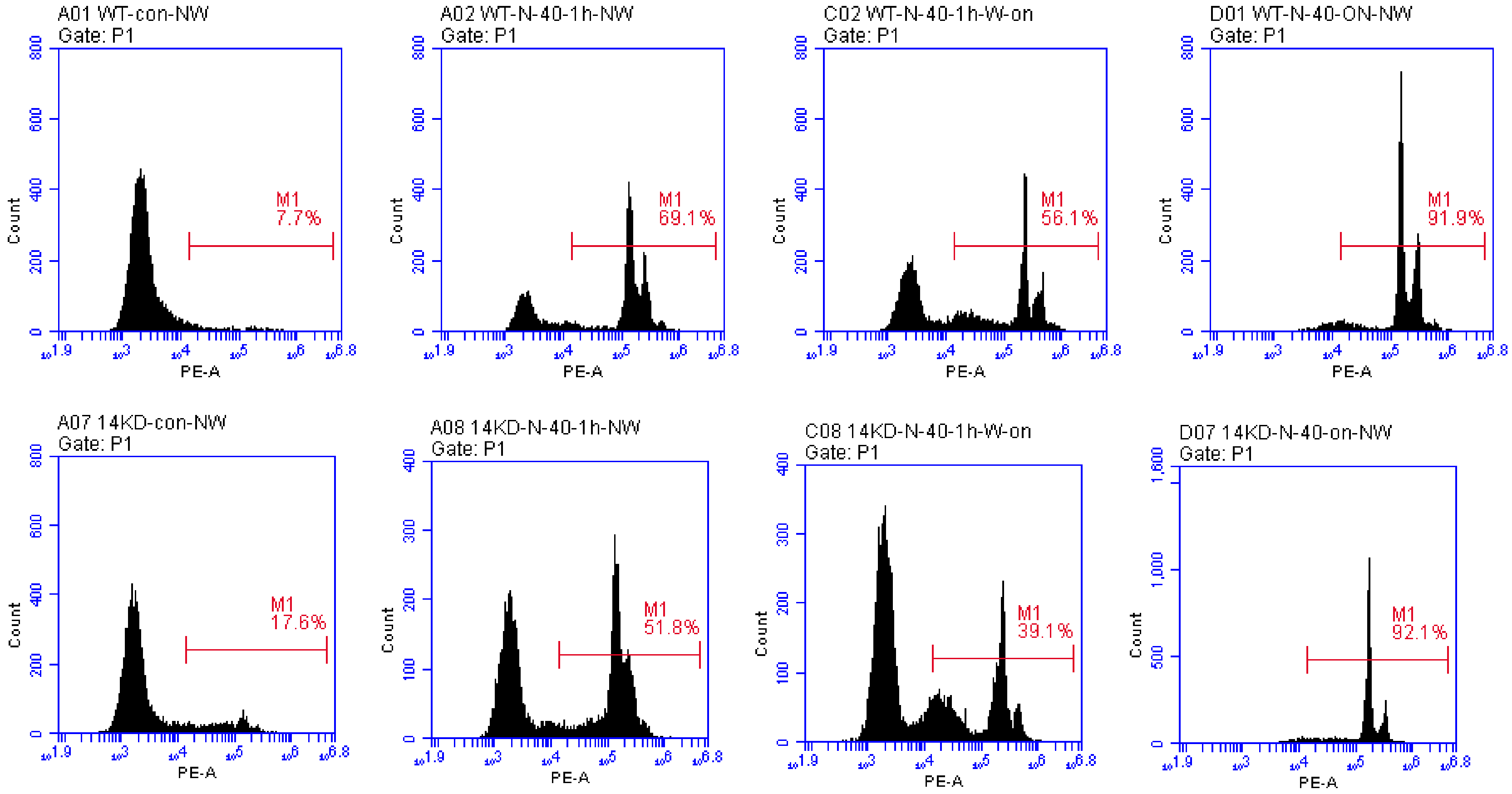

Figure 6B Experiment 2

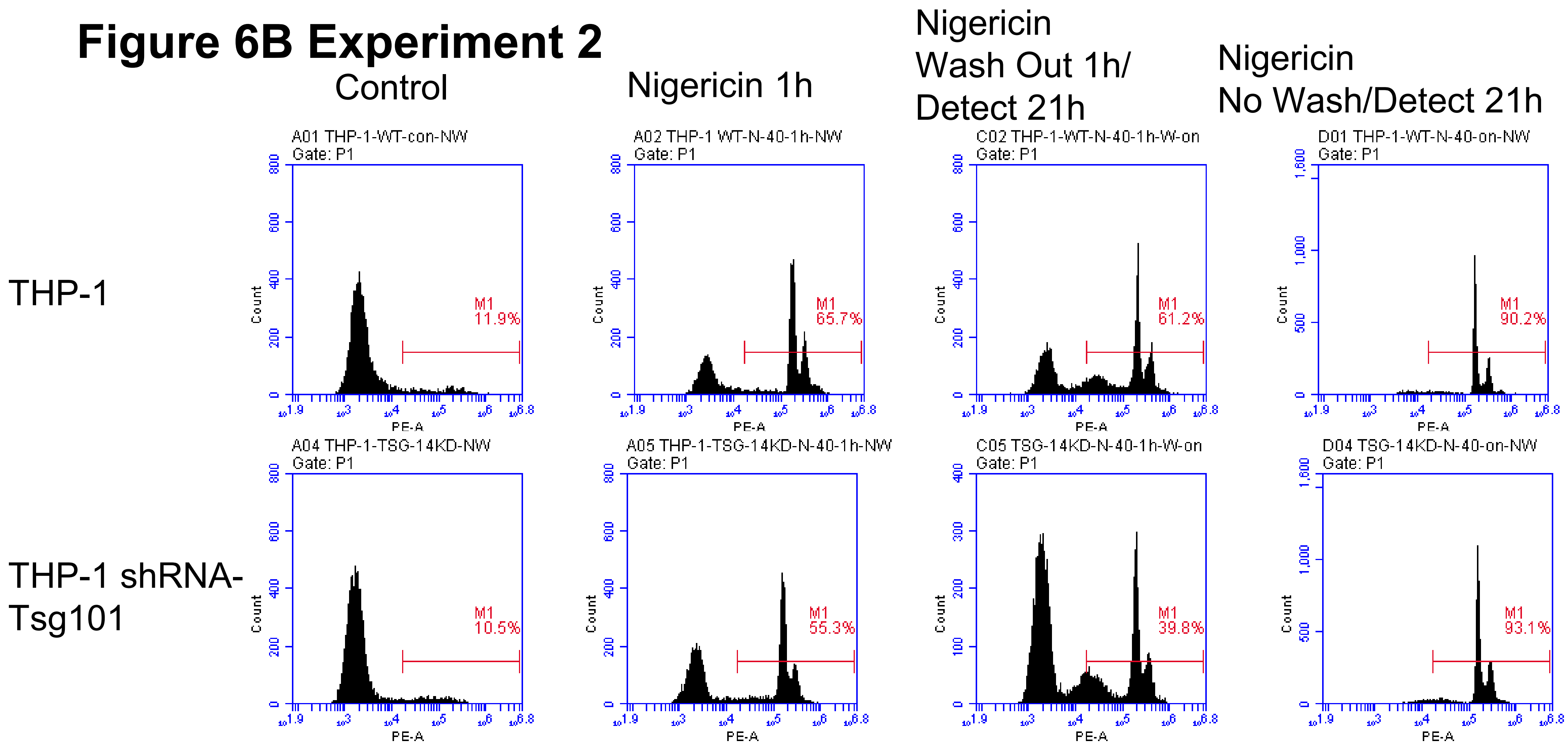

Figure 6B Experiment 3

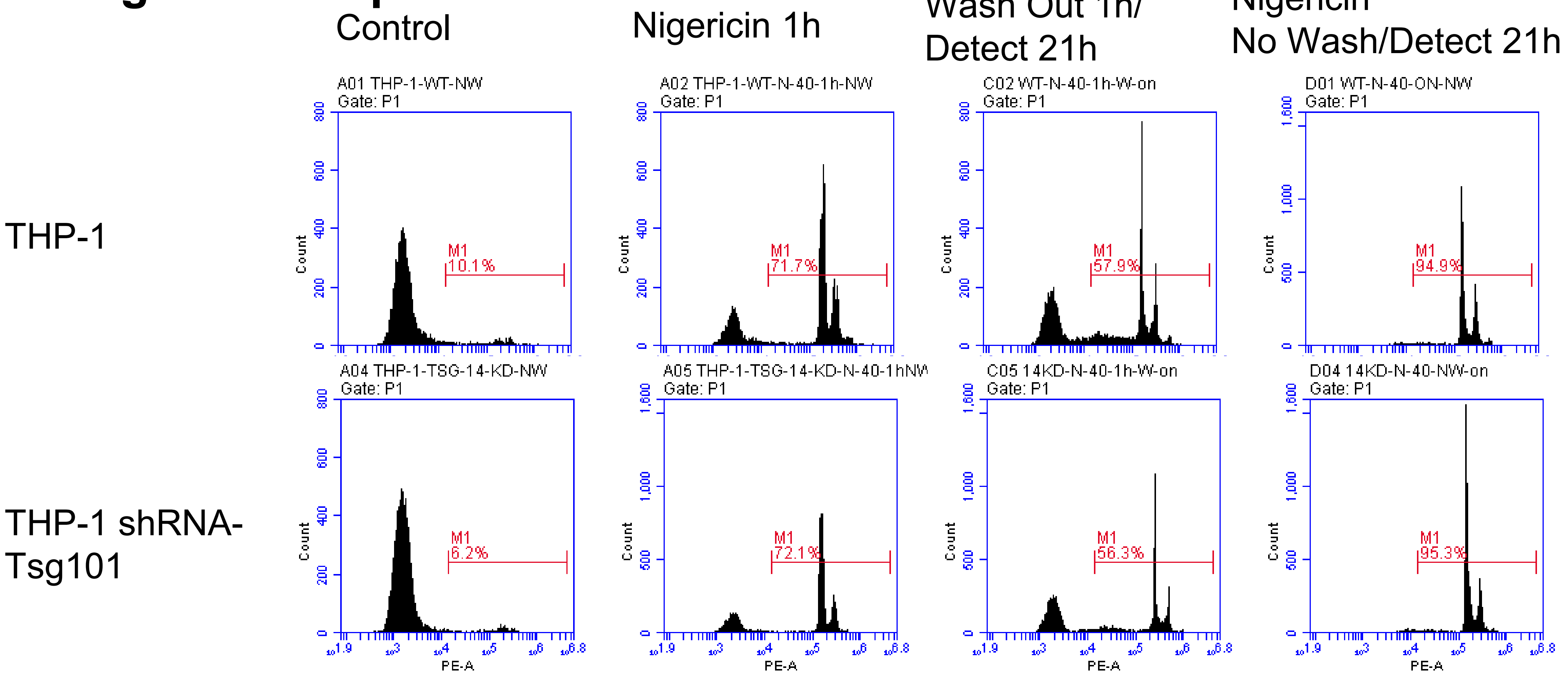

Figure 6B Experiment 4

	Control	Nigericin 1h	Nigericin Wash Out 1h/ Detect 21h	Nigericin No Wash/Detect 21h
THP-1				
THP-1 shRNA-Tsg101				

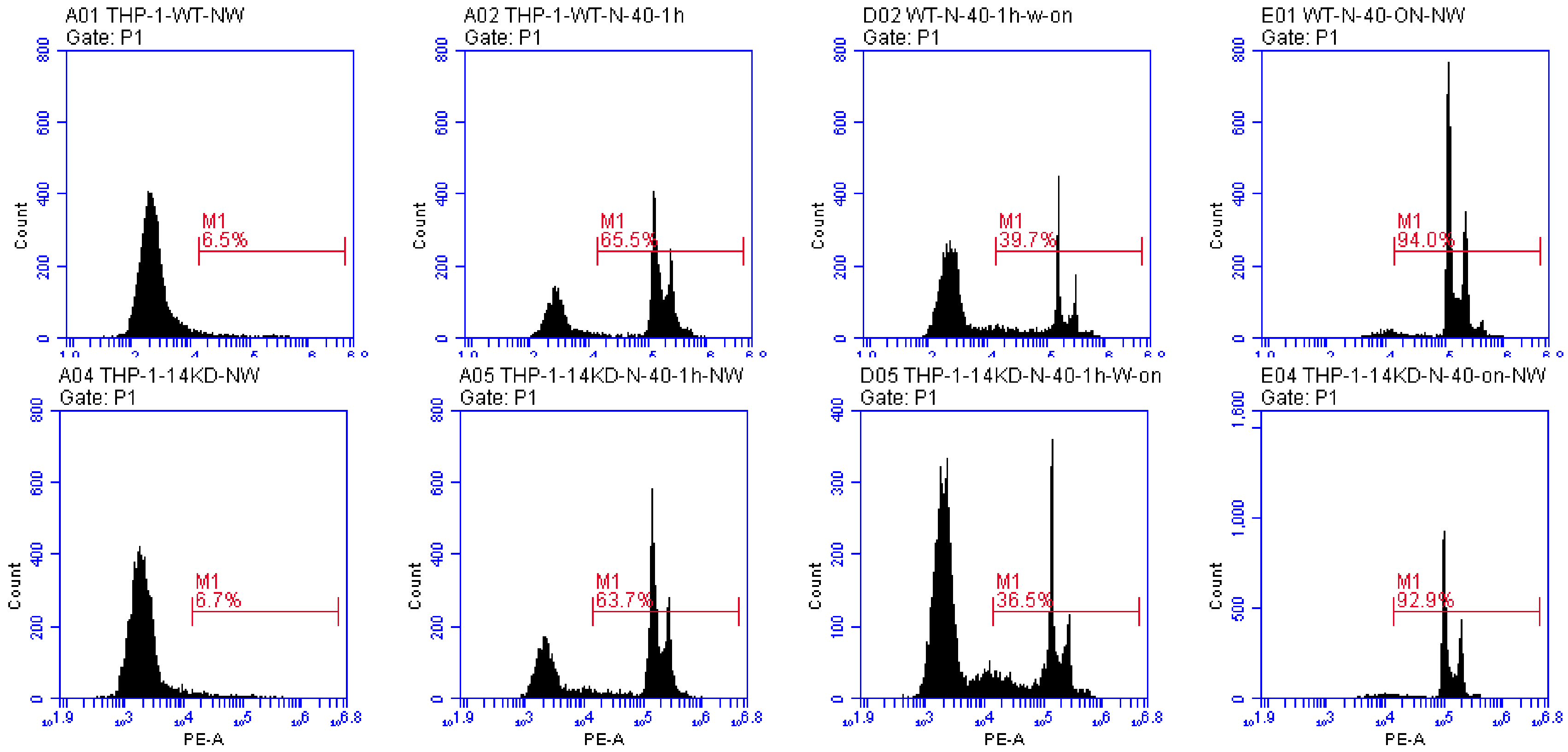

Figure 6C Experiment 1

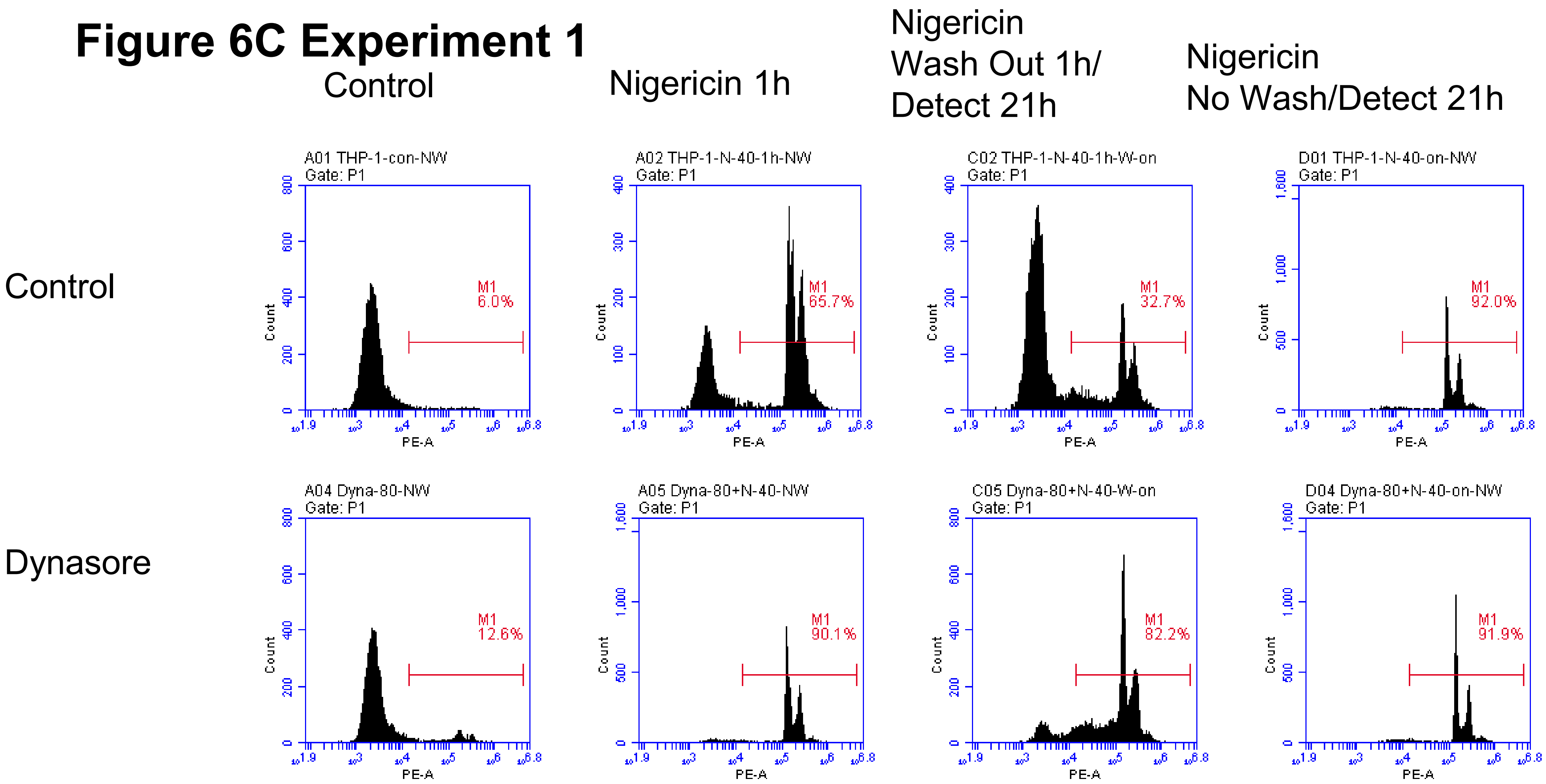

Figure 6C Experiment 2

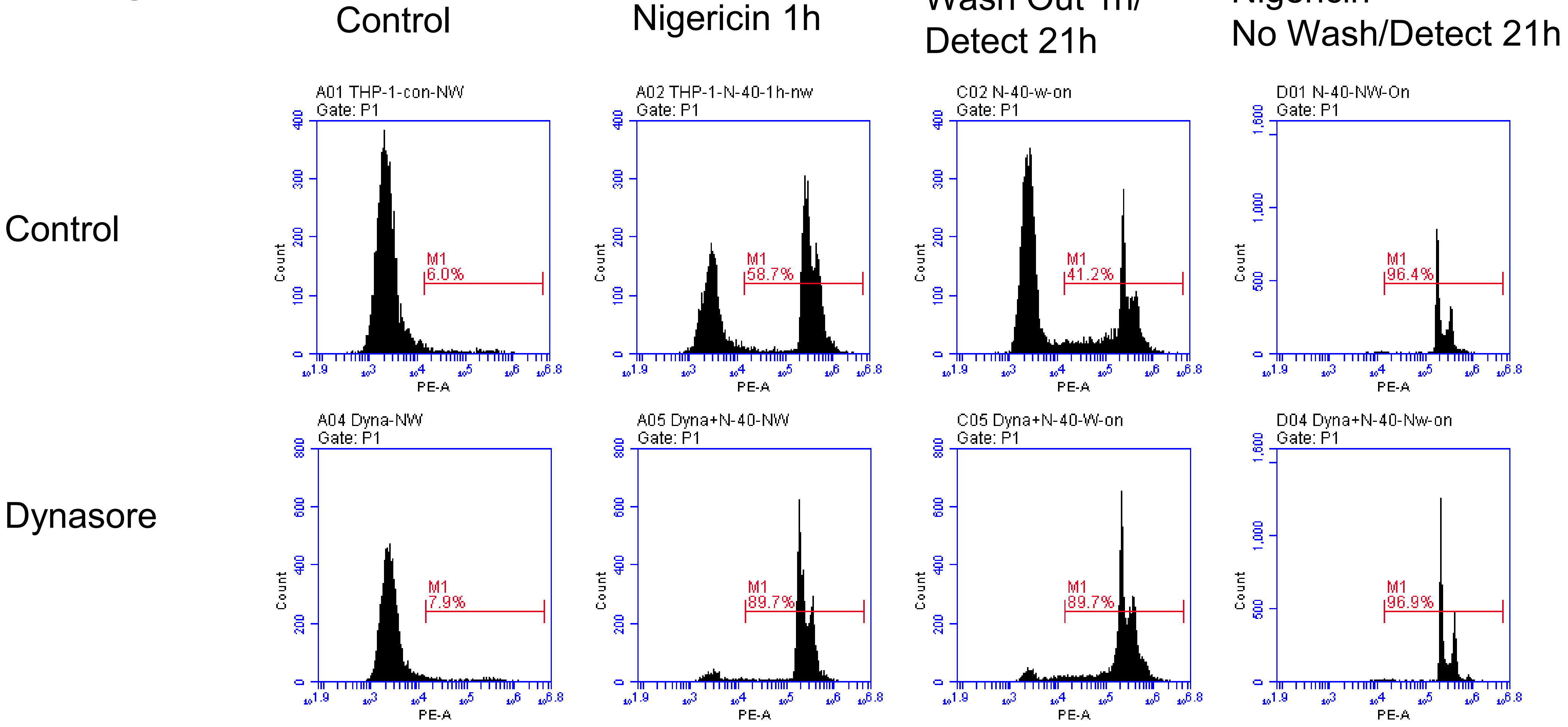

Figure 6C Experiment 3

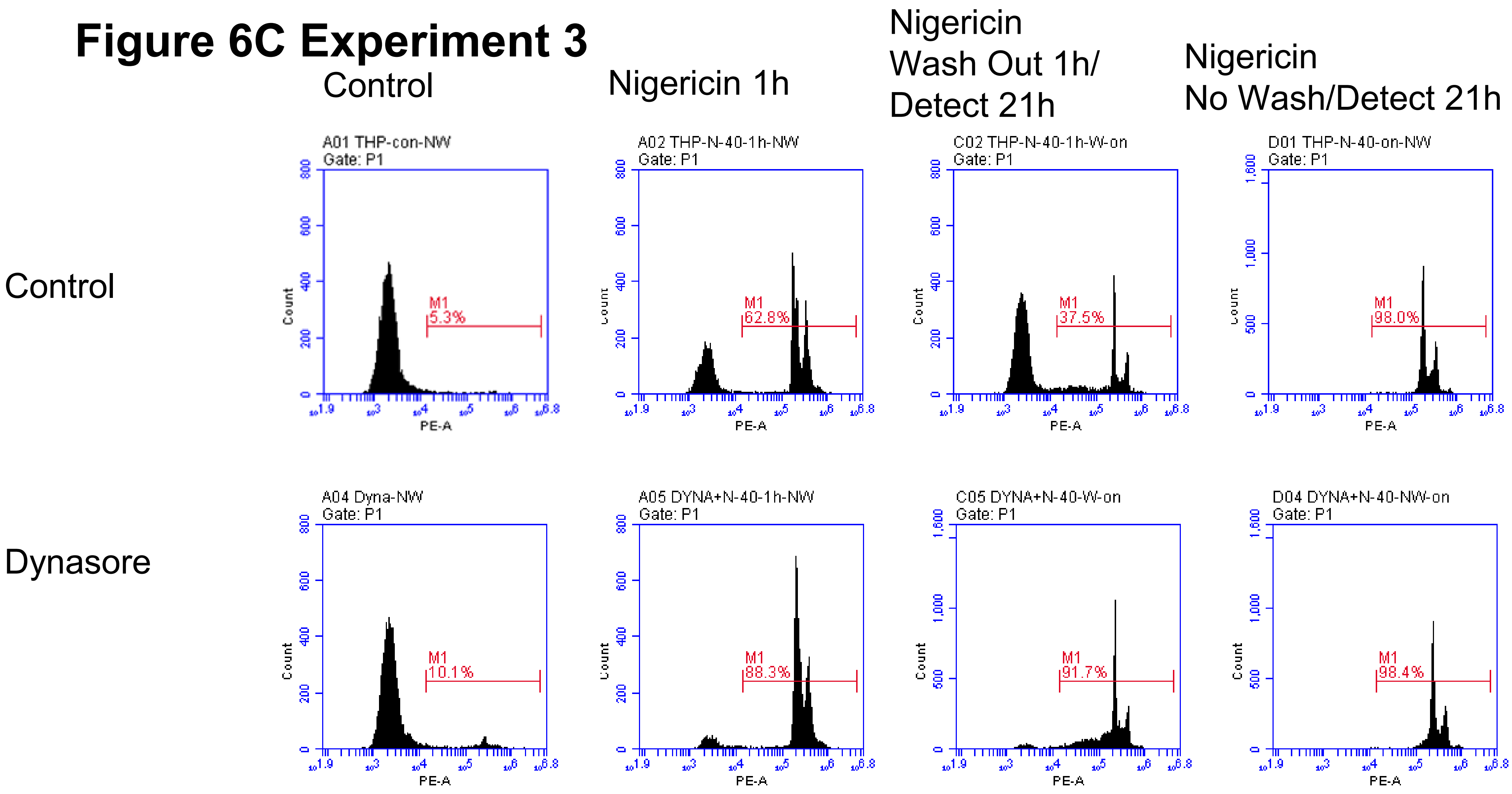

Flow Cytometry Data Supplementary Figures

Experiment 1

Suppl. Figure 2.

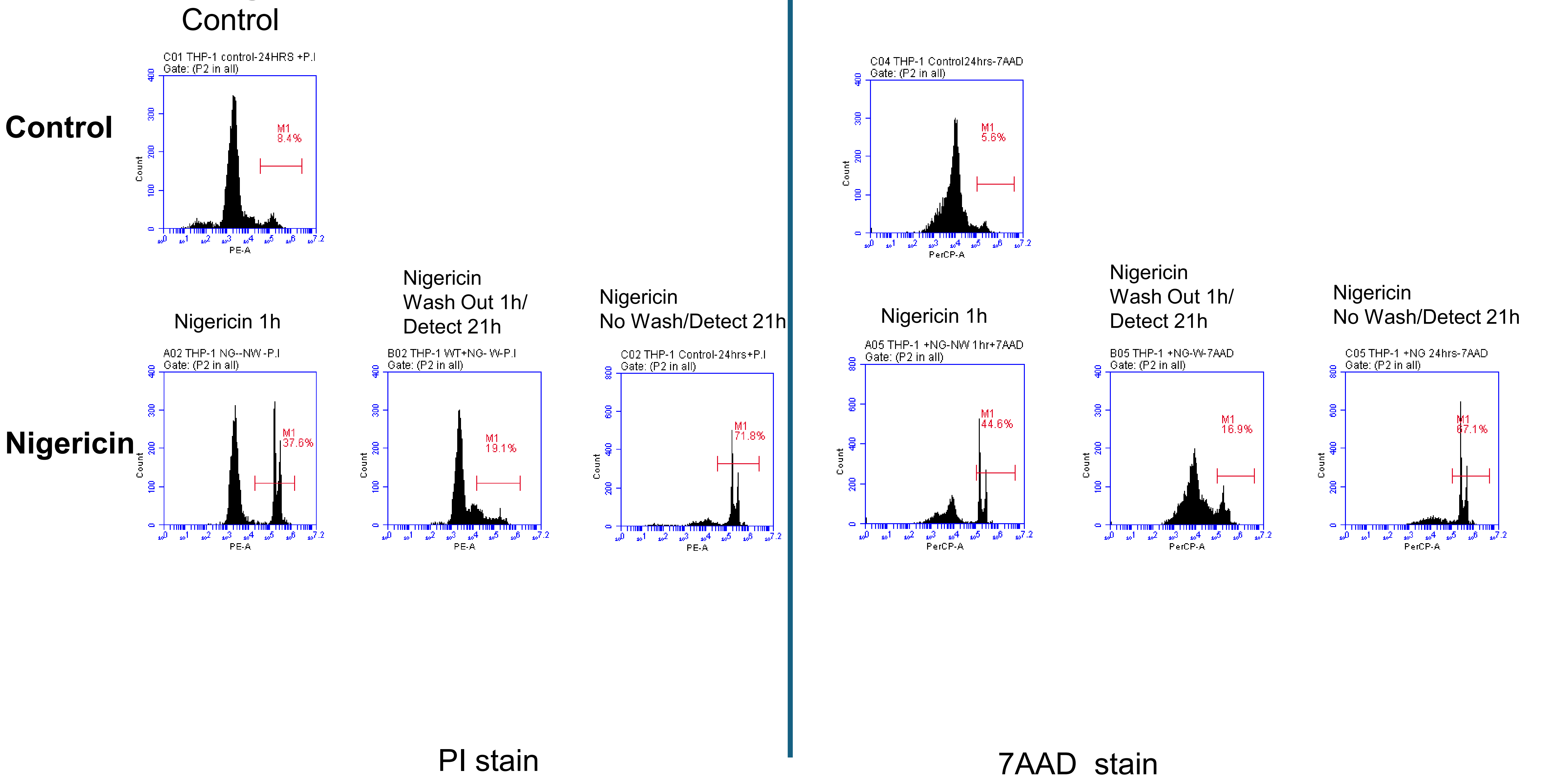

Suppl. Figure 2.

Experiment 2

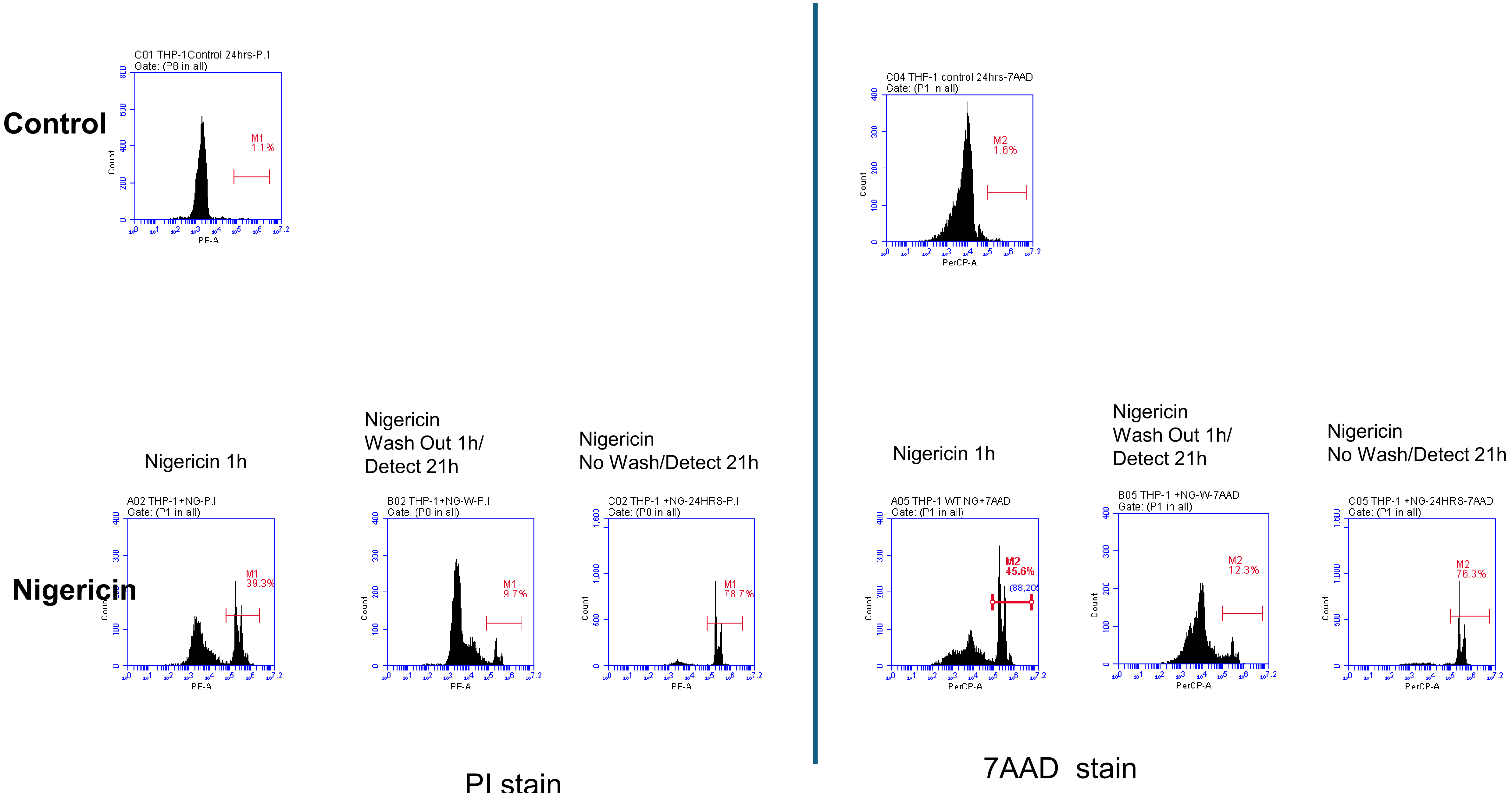

Suppl. Figure 2.

Experiment 3

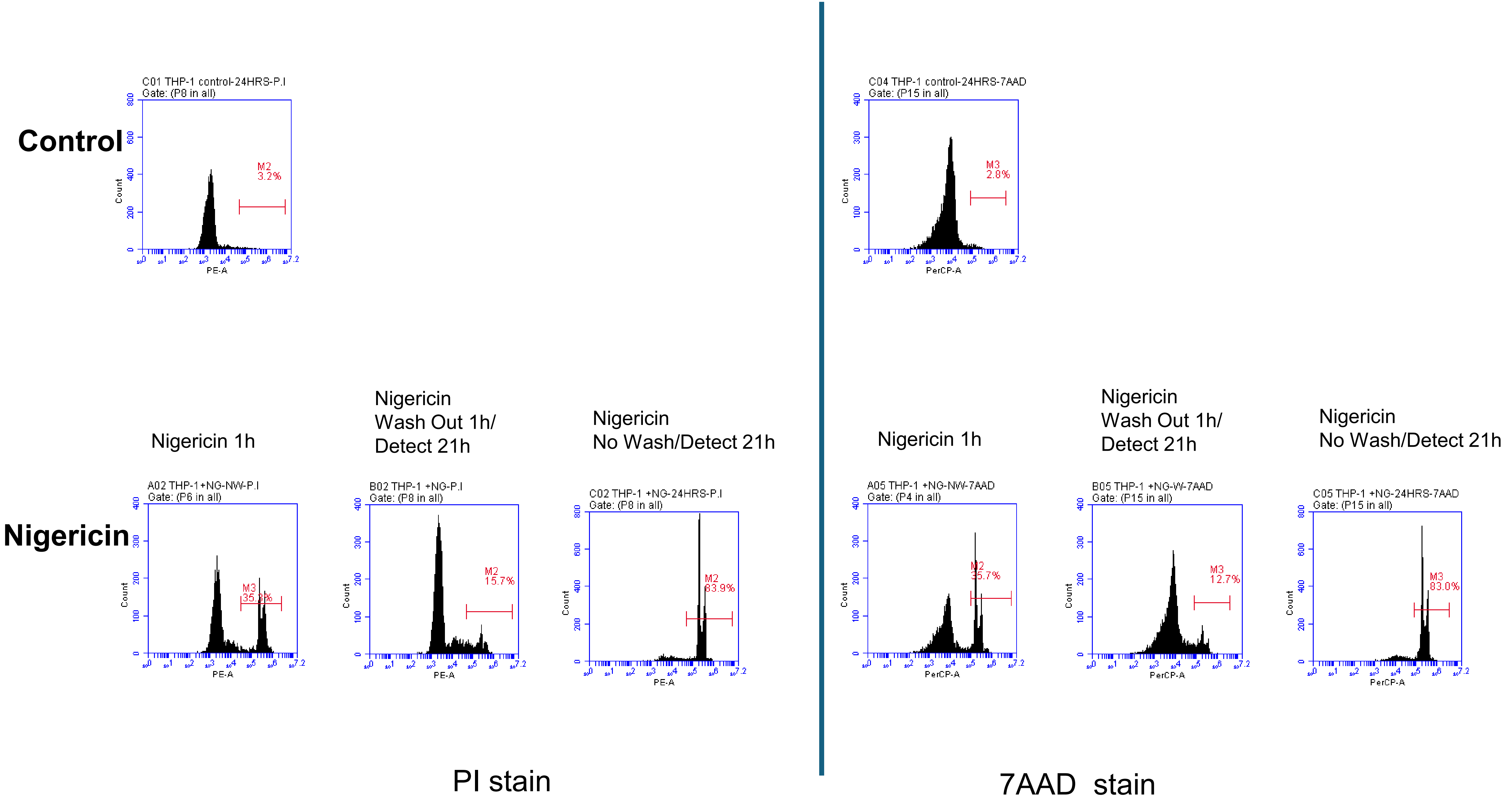

Suppl. Figure 3A

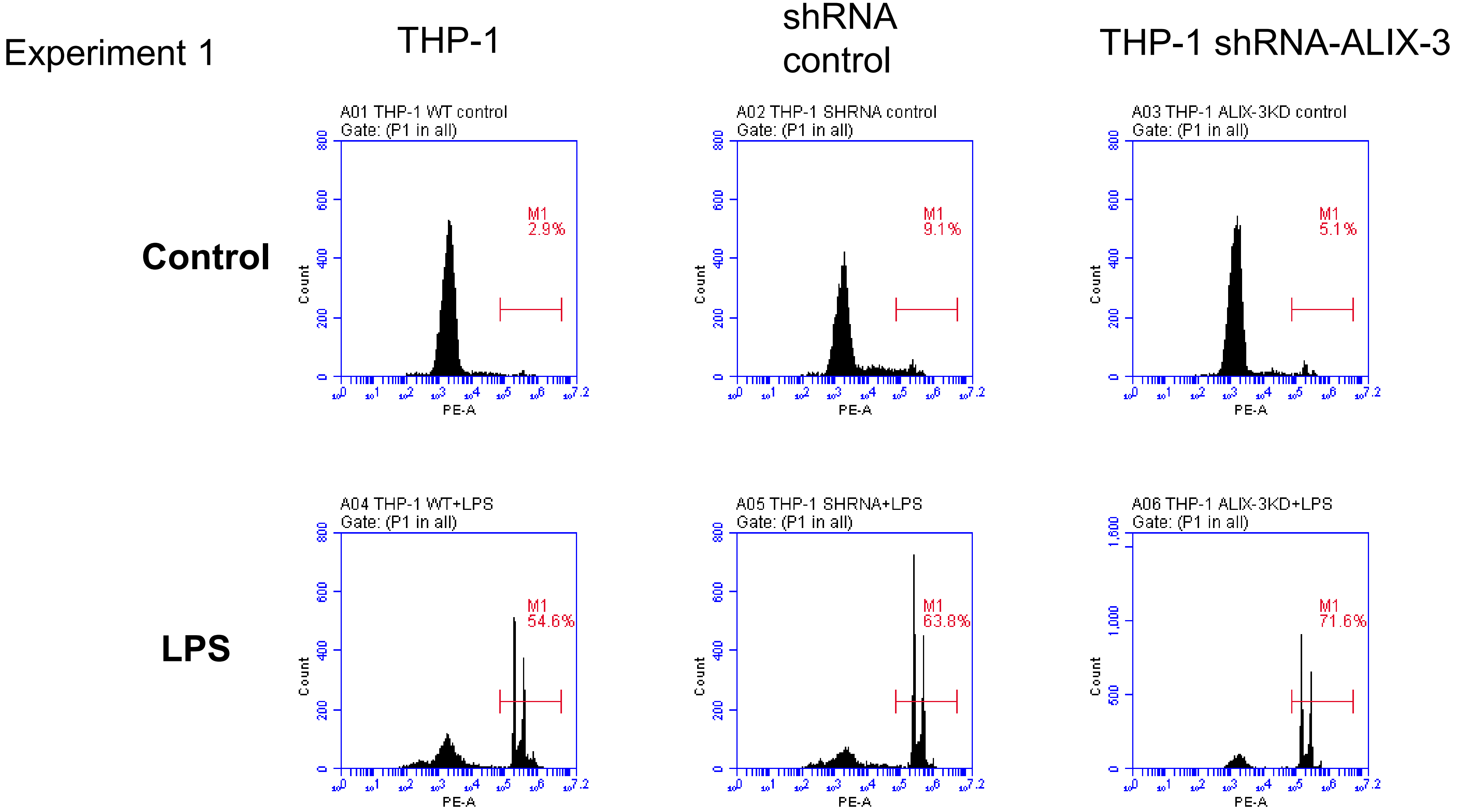

Experiment 2

THP-1 | THP-1 shRNA control | THP-1 shRNA-ALIX-3

Control

LPS

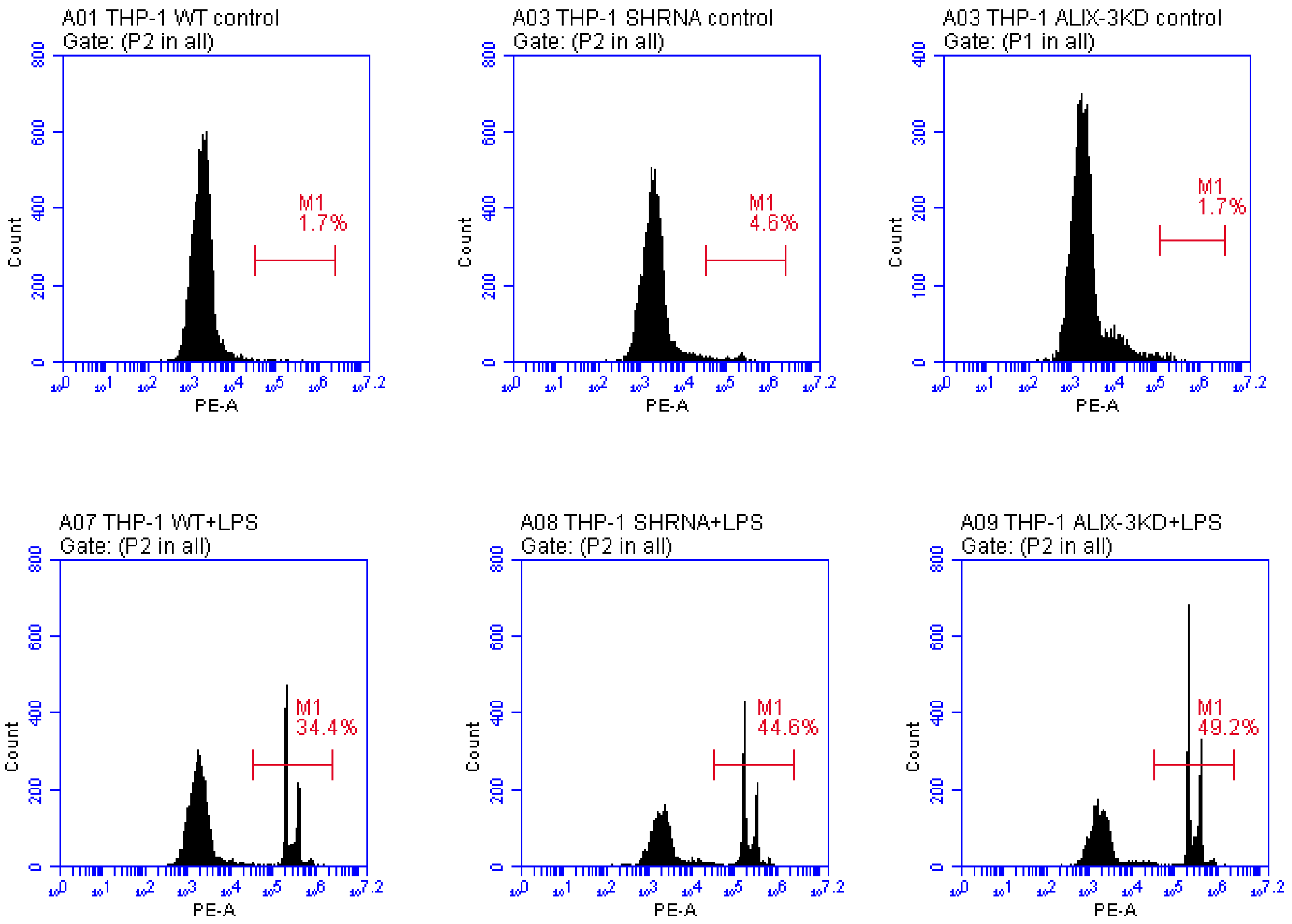

Experiment 3

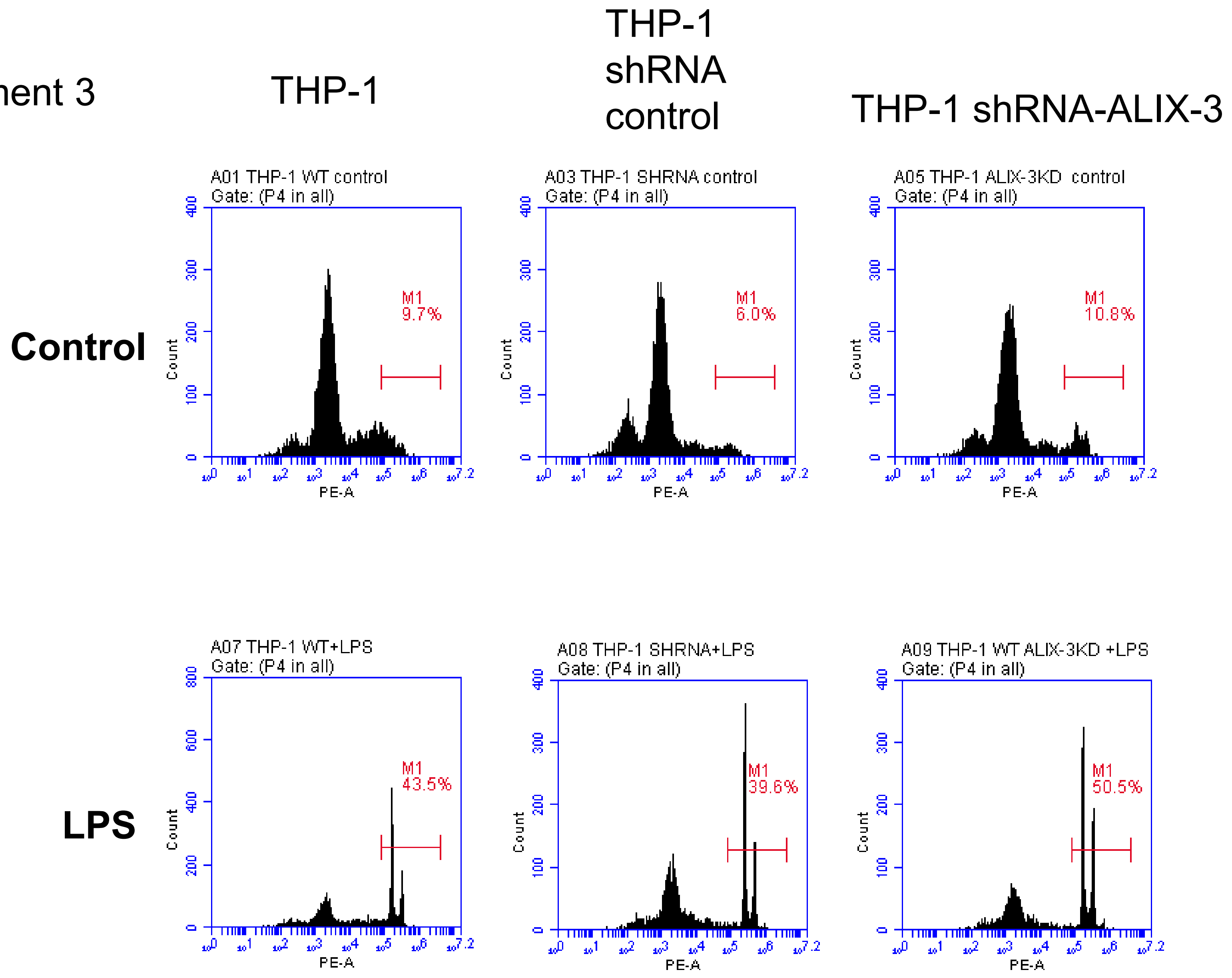

Suppl. Figure 3B

Experiment 1

THP-1 | THP-1 shRNA control | THP-1 shRNA-ALIX-3

Control

Staurosporine

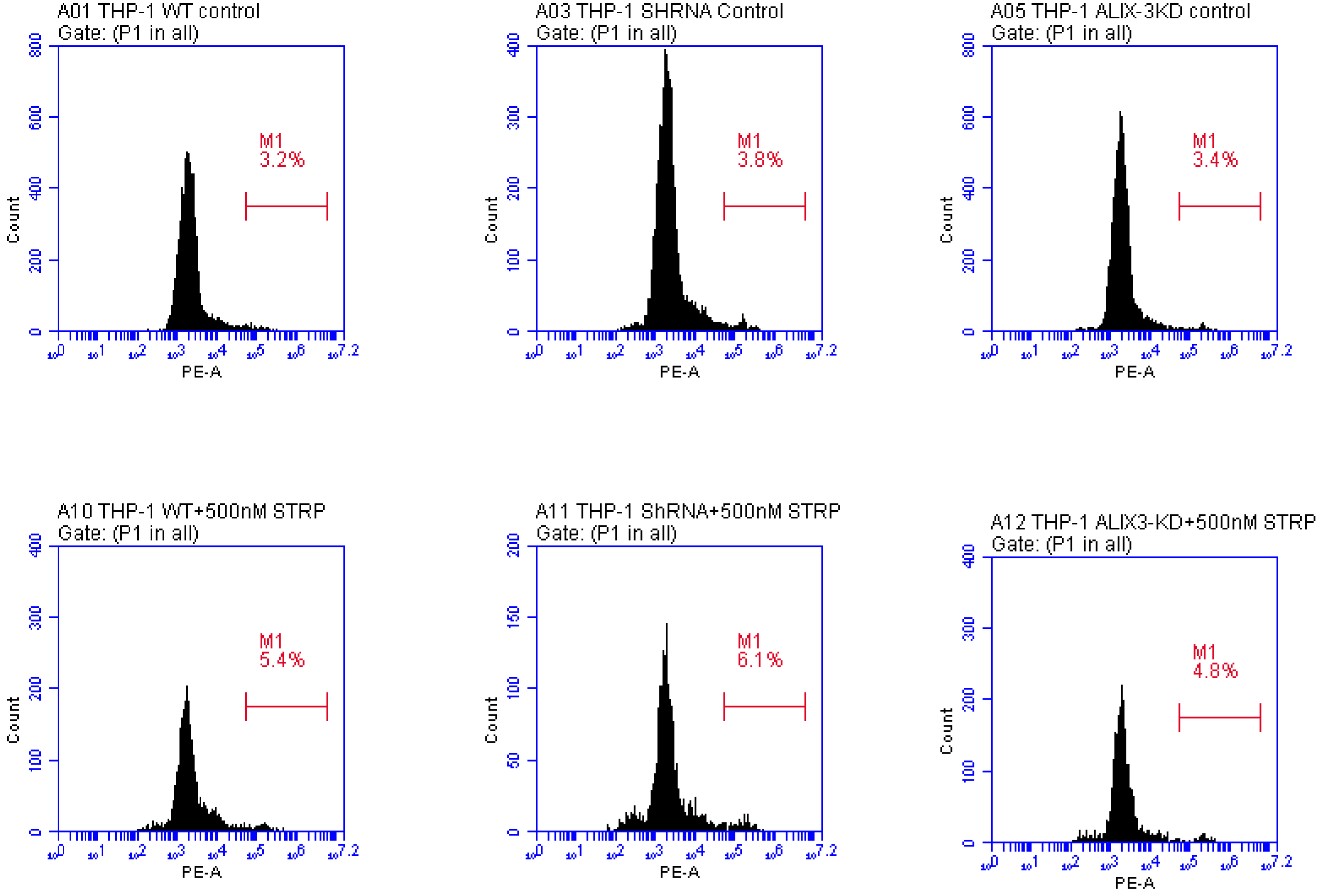

Experiment 2

Suppl. Figure 3B

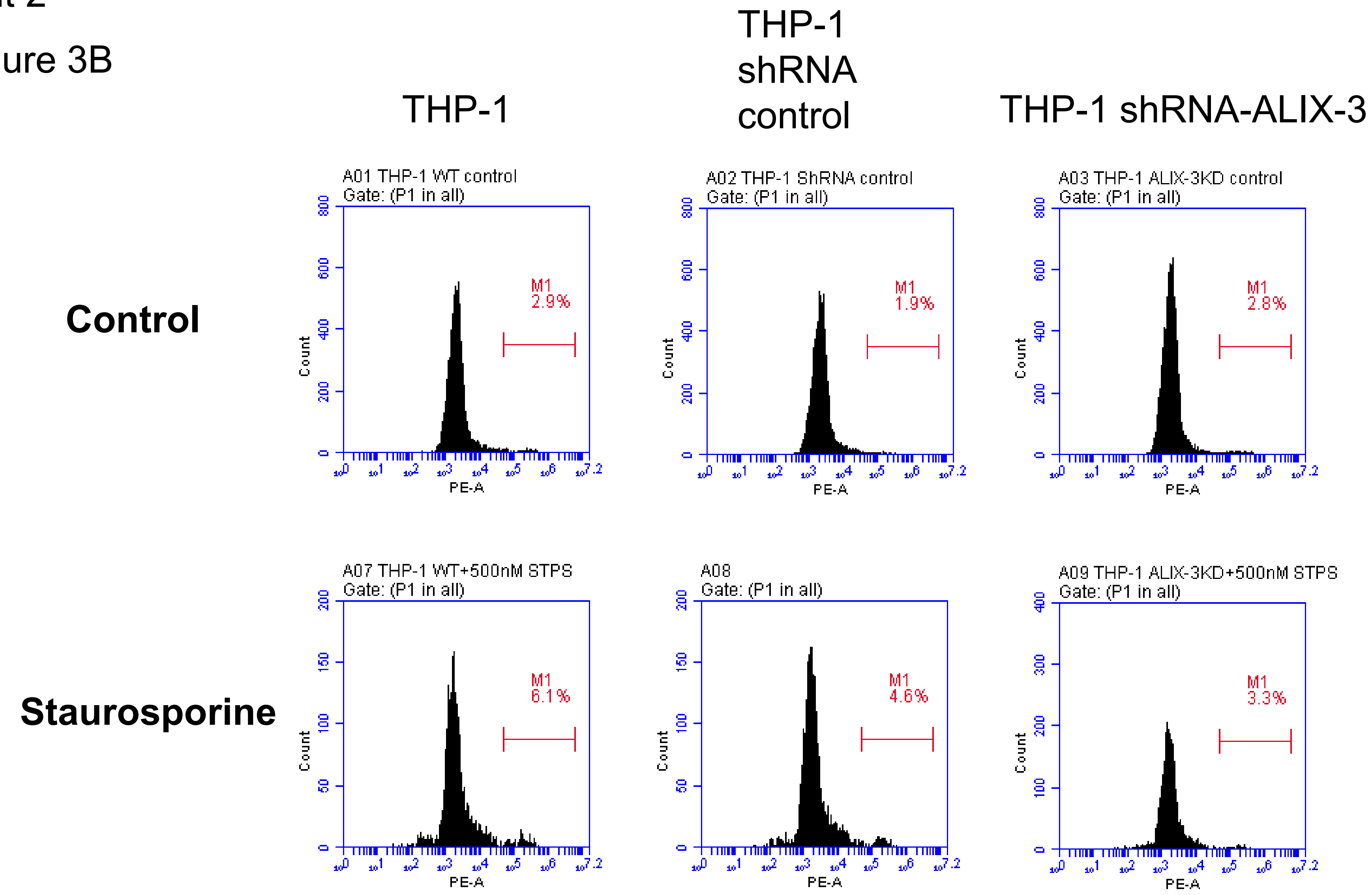

Experiment 3

Suppl. Figure 3B

THP-1 | THP-1 shRNA control | THP-1 shRNA-ALIX-3

Control

Staurosporine

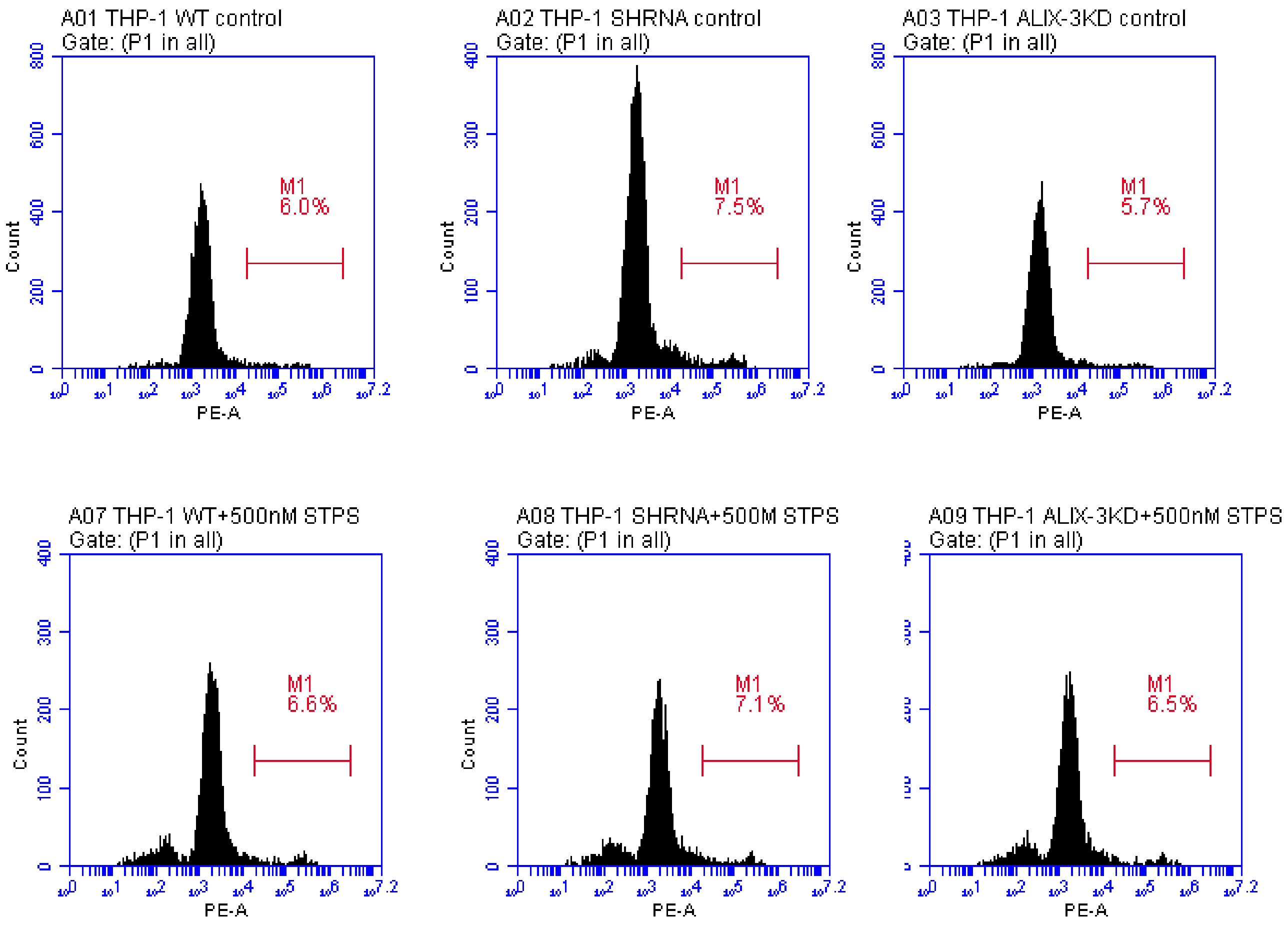

Suppl. Fig 3C

Experiment 1

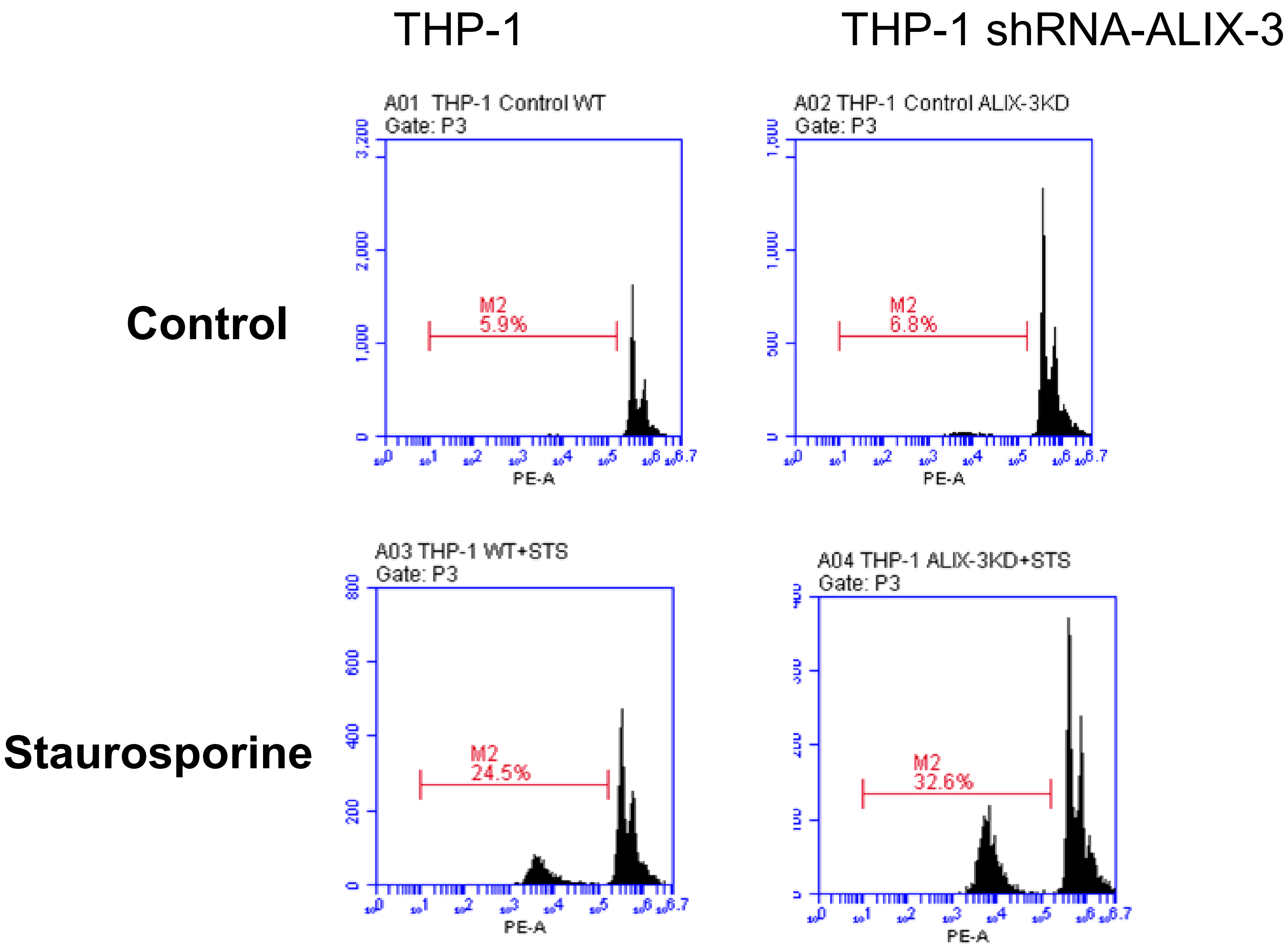

Suppl. Fig 3C

Experiment 2

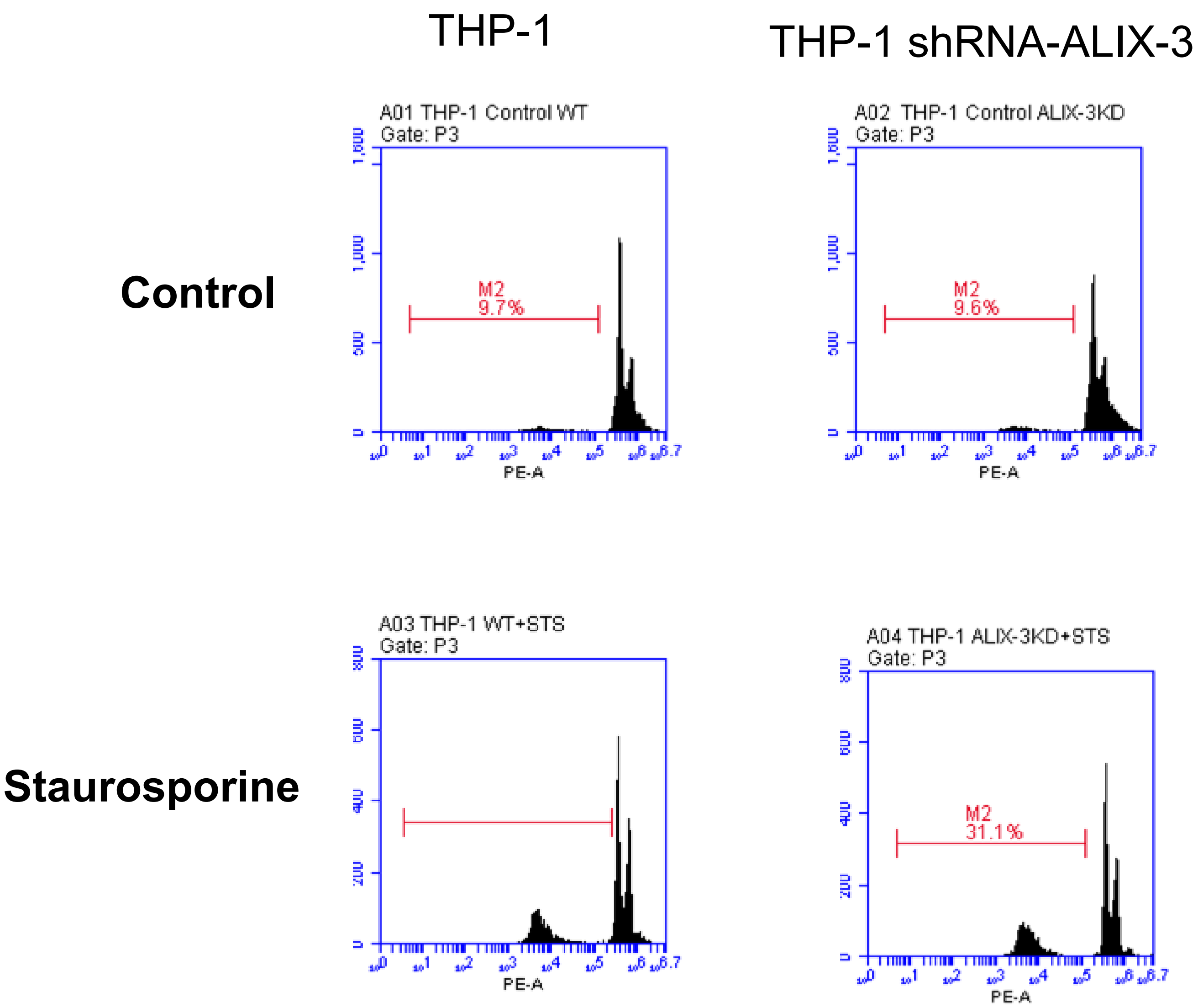

Suppl. Fig 3C

Experiment 3

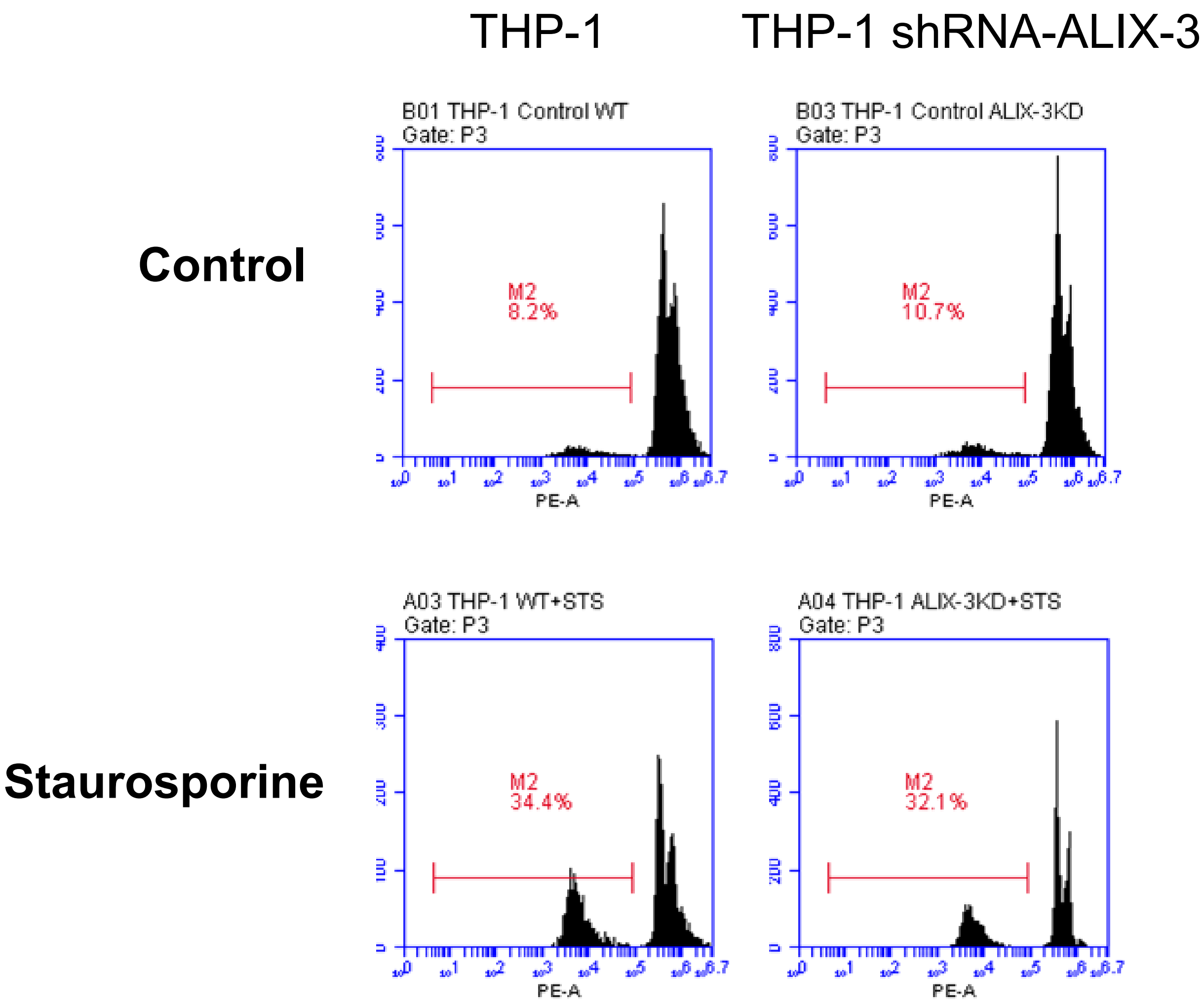

Supplement: Supplementary file 7 — Flow Cytometry Histograms [file 41419_2025_7998_MOESM7_ESM.pdf]
